# Supplementary material for: Excess morbidity and mortality among survivors of childhood acute lymphoblastic leukaemia: 25 years of follow-up from the United Kingdom Childhood Cancer Study (UKCCS) population-based matched cohort
Source: BMJ Open. 2022 Mar 7;12(3):e056216. doi: 10.1136/bmjopen-2021-056216 (PMC8905881; doi:10.1136/bmjopen-2021-056216)

Supplementary Figure 1: Hazard rates per year of activity at paediatrics, haematology and oncology among ALL cases who survived 5 years or more and their matched population controls: UKCCS, ALL diagnosed aged<15 years, 1992-96.

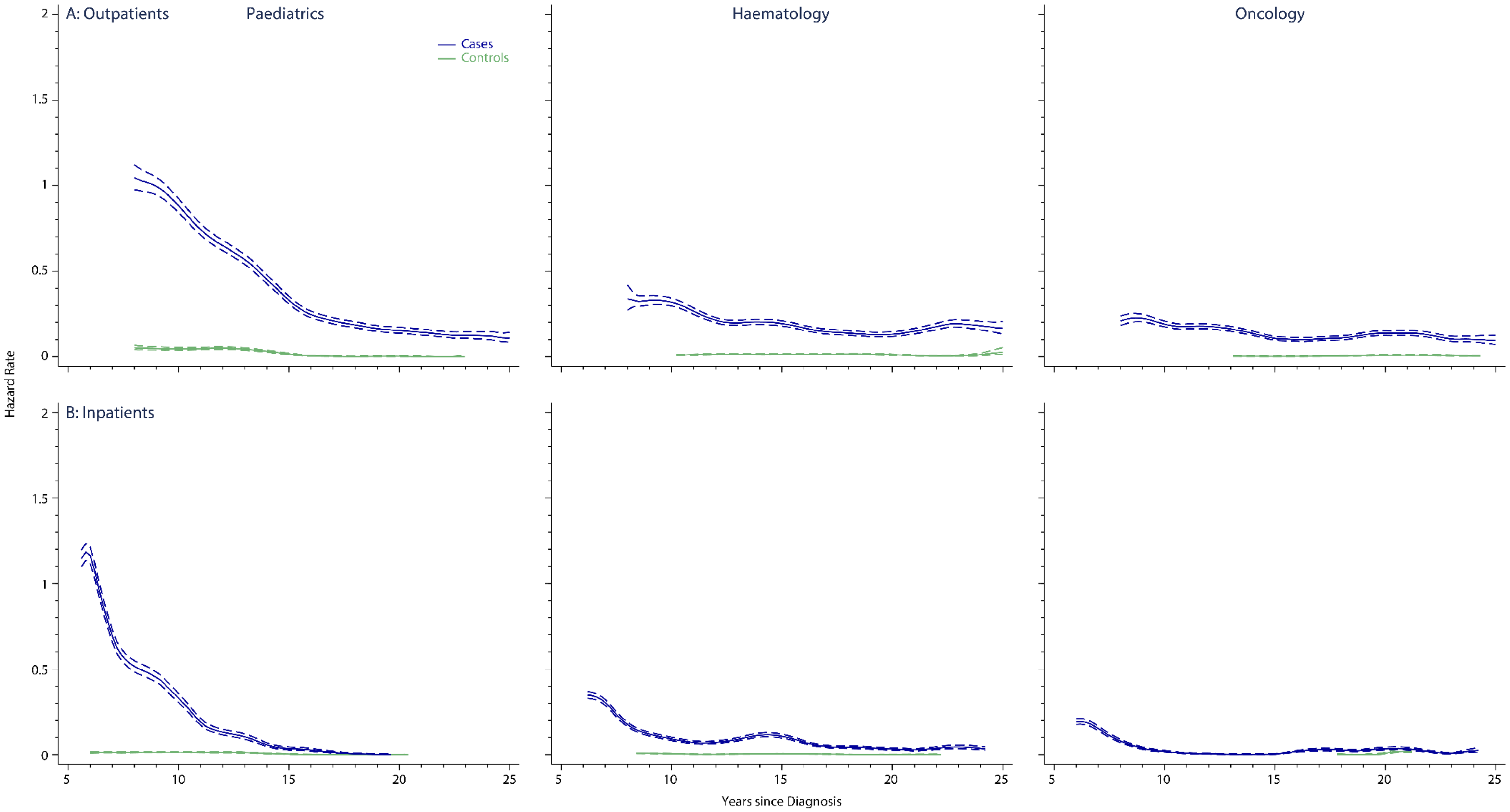

Supplementary Figure 2: Cumulative incidence, rates (per 1000 person-years), attributable risks & incidence rate ratios; top 15 outpatient specialties (excluding paediatrics, haematology & oncology) with ≥2 speciality-specific visits in the 5 to 15 years and 15 to 25 years following diagnosis (cases diagnosed <15 years, 1992-96) and their matched controls

A: Males

£15 Years

	Incidence Rate Cases	Incidence Rate Controls	Attributable Risk (95%CI)	Incidence Rate Ratio (95%CI)
Endocrinology	29.1	0.5	28.6(22.4,34.8)	61.3(20.3,303)
Cardiology/Cardiosurgery	19.0	1.1	17.9(13.0,22.9)	17.1(7.8,44.4)
General medicine	17.5	2.1	15.5(10.7,20.2)	8.5(4.6,16.9)
Ophthalmology	18.6	2.7	15.8(10.8,20.8)	6.8(3.9,12.4)
Respiratory medicine	3.6	0.6	3.0(0.8,5.1)	5.7(1.7,24.1)
Neurology/Neurosurgery	6.4	1.8	4.6(1.7,7.5)	3.6(1.7,8.3)
GP/Nursing/Allied professionals	13.9	4.0	9.8(5.5,14.2)	3.5(2.1,5.9)
Gastroenterology	2.1	0.6	1.4(−0.2,3.1)	3.3(0.8,15.2)
Oral/Dental	21.1	8.6	12.5(6.9,18.2)	2.5(1.7,3.6)
General/Paediatric surgery	8.7	3.6	5.1(1.6,8.7)	2.4(1.3,4.5)
Urology	4.5	2.3	2.3(−0.3,4.9)	2.0(0.9,4.5)
Plastic surgery	4.8	2.4	2.4(−0.3,5.0)	2.0(0.9,4.3)
ENT/Audiology	12.2	6.4	5.8(1.5,10.2)	1.9(1.2,3.1)
Dermatology	11.9	8.2	3.8(−0.6,8.2)	1.5(0.9,2.3)
Trauma & orthopaedics	38.0	38.7	−0.7(−9.6,8.1)	1.0(0.8,1.2)

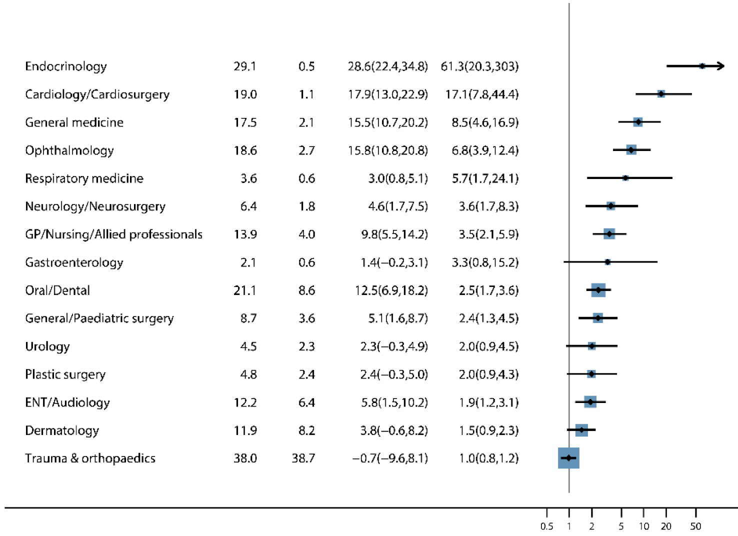

>15–25 Years

	Incidence Rate Cases	Incidence Rate Controls	Attributable Risk (95%CI)	Incidence Rate Ratio (95%CI)
Endocrinology	7.5	0.5	7.0(4.2,9.8)	16.4(5.7,64.5)
General medicine	12.1	1.9	10.2(6.5,13.8)	6.4(3.5,12.1)
Cardiology/Cardiosurgery	12.9	2.2	10.7(6.8,14.7)	5.8(3.3,10.6)
Respiratory medicine	3.5	0.9	2.6(0.8,4.5)	3.9(1.6,10.5)
Neurology/Neurosurgery	4.9	2.2	2.7(0.4,5.0)	2.2(1.1,4.3)
Gastroenterology	4.3	2.3	2.0(−0.1,4.1)	1.9(1.0,3.7)
Urology	5.8	3.2	2.6(0.0,5.1)	1.8(1.0,3.2)
Dermatology	5.3	3.1	2.3(−0.3,4.8)	1.7(0.9,3.2)
ENT/Audiology	7.4	4.5	2.8(−0.2,5.9)	1.6(1.0,2.7)
GP/Nursing/Allied professionals	20.2	12.9	7.3(2.0,12.7)	1.6(1.1,2.1)
General/Paediatric surgery	5.2	3.7	1.5(−1.0,4.1)	1.4(0.8,2.5)
Plastic surgery	3.1	2.7	0.4(−1.6,2.3)	1.1(0.5,2.3)
Ophthalmology	3.7	3.6	0.1(−2.2,2.3)	1.0(0.5,2.0)
Trauma & orthopaedics	15.1	18.0	−2.9(−8.4,2.6)	0.8(0.6,1.2)
Oral/Dental	6.1	7.3	−1.2(−4.3,1.9)	0.8(0.5,1.4)

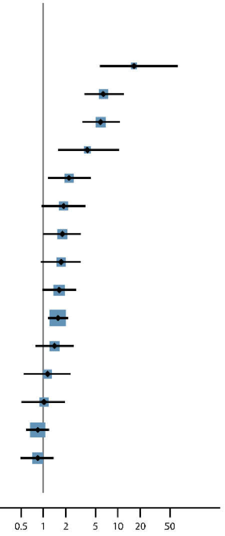

B: Females

£15 Years

	Incidence Rate Cases	Incidence Rate Controls	Attributable Risk (95%CI)	Incidence Rate Ratio (95%CI)
Endocrinology	20.0	0.7	19.2(13.7,24.7)	26.7(9.8,102)
Cardiology/Cardiosurgery	20.3	1.9	18.5(12.9,24.1)	10.8(5.4,23.8)
Respiratory medicine	5.0	0.6	4.5(1.8,7.2)	8.9(2.5,48.5)
General medicine	16.1	2.6	13.5(8.5,18.5)	6.1(3.3,12.1)
Plastic surgery	6.1	2.3	3.8(0.6,7.0)	2.7(1.2,6.1)
Dermatology	17.4	7.4	10.0(4.5,15.6)	2.4(1.5,3.7)
General/Paediatric surgery	12.8	5.7	7.1(2.4,11.8)	2.2(1.3,3.8)
GP/Nursing/Allied professionals	13.4	6.3	7.1(2.3,11.9)	2.1(1.3,3.5)
Neurology/Neurosurgery	5.0	2.6	2.3(−0.6,5.3)	1.9(0.8,4.3)
Ophthalmology	10.8	5.9	4.8(0.4,9.3)	1.8(1.1,3.1)
ENT/Audiology	10.2	6.0	4.2(−0.1,8.5)	1.7(1.0,2.9)
Oral/Dental	22.6	13.9	8.7(2.1,15.4)	1.6(1.1,2.3)
Trauma & orthopaedics	20.0	16.4	3.6(−2.9,10.1)	1.2(0.8,1.7)
Obstetrics & gynaecology	27.9	28.5	−0.6(−8.5,7.3)	1.0(0.7,1.3)
Midwifery	3.1	5.1	−1.9(−4.7,0.9)	0.6(0.3,1.4)

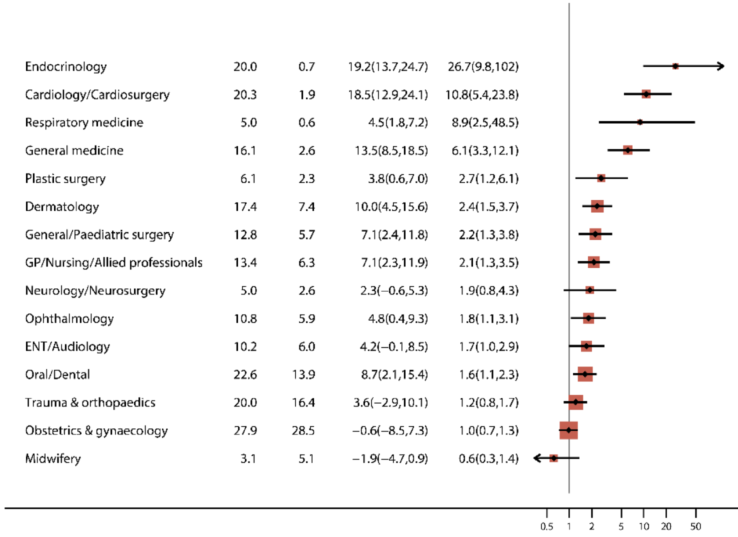

>15–25 Years

	Incidence Rate Cases	Incidence Rate Controls	Attributable Risk (95%CI)	Incidence Rate Ratio (95%CI)
Cardiology/Cardiosurgery	16.1	1.7	14.4(9.7,19.0)	9.3(4.9,18.7)
Endocrinology	5.9	1.3	4.6(1.9,7.2)	4.5(2.0,10.7)
General medicine	12.4	4.0	8.4(4.3,12.4)	3.1(1.9,5.2)
Respiratory medicine	2.5	1.0	1.5(−0.2,3.2)	2.4(0.9,7.0)
Ophthalmology	6.9	3.2	3.7(0.7,6.6)	2.1(1.2,3.9)
Neurology/Neurosurgery	5.4	2.8	2.5(−0.1,5.2)	1.9(1.0,3.7)
Plastic surgery	3.6	2.1	1.5(−0.7,3.6)	1.7(0.8,3.7)
GP/Nursing/Allied professionals	32.6	22.3	10.3(2.9,17.6)	1.5(1.1,1.9)
General/Paediatric surgery	13.5	10.7	2.8(−1.8,7.5)	1.3(0.9,1.9)
Obstetrics & gynaecology	66.6	58.0	8.6(−3.7,21.0)	1.1(0.9,1.4)
ENT/Audiology	6.9	6.2	0.6(−2.6,3.9)	1.1(0.6,1.8)
Oral/Dental	9.8	9.1	0.7(−3.4,4.9)	1.1(0.7,1.7)
Midwifery	25.3	26.6	−1.3(−7.8,5.2)	1.0(0.7,1.2)
Dermatology	7.4	9.1	−1.8(−5.4,1.9)	0.8(0.5,1.3)
Trauma & orthopaedics	9.6	12.1	−2.5(−6.8,1.8)	0.8(0.5,1.2)

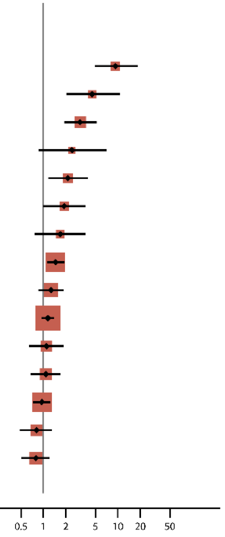

Supplement: Supplementary data [file bmjopen-2021-056216supp001.pdf]
