# Supplementary material for: Excess morbidity and mortality among survivors of childhood acute lymphoblastic leukaemia: 25 years of follow-up from the United Kingdom Childhood Cancer Study (UKCCS) population-based matched cohort
Source: BMJ Open. 2022 Mar 7;12(3):e056216. doi: 10.1136/bmjopen-2021-056216 (PMC8905881; doi:10.1136/bmjopen-2021-056216)
Supplement: Author's manuscript [file bmjopen-2021-056216.draft_revisions.pdf]

BMJ Open

BMJ Open is committed to open peer review. As part of this commitment we make the peer review history of every article we publish publicly available.

When an article is published we post the peer reviewers' comments and the authors' responses online. We also post the versions of the paper that were used during peer review. These are the versions that the peer review comments apply to.

The versions of the paper that follow are the versions that were submitted during the peer review process. They are not the versions of record or the final published versions. They should not be cited or distributed as the published version of this manuscript.

BMJ Open is an open access journal and the full, final, typeset and author-corrected version of record of the manuscript is available on our site with no access controls, subscription charges or pay-per-view fees (http://bmjopen.bmj.com).

If you have any questions on BMJ Open's open peer review process please email info.bmjopen@bmj.com

BMJ Open

Excess morbidity and mortality among survivors of childhood acute lymphoblastic leukaemia: 25 years of follow-up from the United Kingdom Childhood Cancer Study (UKCCS)

Journal:	*BMJ Open*
Manuscript ID	bmjopen-2021-056216
Article Type:	Original research
Date Submitted by the Author:	06-Aug-2021
Complete List of Authors:	Kane, Eleanor; University of York,<br>Kinsey, Sally; Leeds Teaching Hospitals NHS Trust<br>Bonaventure, Audrey; INSERM, Epidemiology of childhood and adolescent cancers team<br>Johnston, Tom; University of York, Department of Health Sciences<br>Simpson, Jill; University of York, Department of Health Sciences<br>Howell, Debra; University of York, Health Sciences<br>Smith, Alexandra; University of York, Health Sciences<br>Roman, Eve; University of York, Health Sciences
Keywords:	Leukaemia < HAEMATOLOGY, Epidemiology < INFECTIOUS DISEASES, Paediatric oncology < ONCOLOGY, PUBLIC HEALTH

SCHOLARONE™
Manuscripts

I, the Submitting Author has the right to grant and does grant on behalf of all authors of the Work (as defined in the below author licence), an exclusive licence and/or a non-exclusive licence for contributions from authors who are: i) UK Crown employees; ii) where BMJ has agreed a CC-BY licence shall apply, and/or iii) in accordance with the terms applicable for US Federal Government officers or employees acting as part of their official duties; on a worldwide, perpetual, irrevocable, royalty-free basis to BMJ Publishing Group Ltd ("BMJ") its licensees and where the relevant Journal is co-owned by BMJ to the co-owners of the Journal, to publish the Work in this journal and any other BMJ products and to exploit all rights, as set out in our licence.

The Submitting Author accepts and understands that any supply made under these terms is made by BMJ to the Submitting Author unless you are acting as an employee on behalf of your employer or a postgraduate student of an affiliated institution which is paying any applicable article publishing charge ("APC") for Open Access articles. Where the Submitting Author wishes to make the Work available on an Open Access basis (and intends to pay the relevant APC), the terms of reuse of such Open Access shall be governed by a Creative Commons licence – details of these licences and which Creative Commons licence will apply to this Work are set out in our licence referred to above.

Other than as permitted in any relevant BMJ Author's Self Archiving Policies, I confirm this Work has not been accepted for publication elsewhere, is not being considered for publication elsewhere and does not duplicate material already published. I confirm all authors consent to publication of this Work and authorise the granting of this licence.

For peer review only

Excess morbidity and mortality among survivors of childhood acute lymphoblastic leukaemia: 25 years of follow-up from the United Kingdom Childhood Cancer Study (UKCCS)

Eleanor Kane[1], Sally Kinsey [1,2], Audrey Bonaventure[3], Tom Johnston[1], Jill Simpson[1], Debra Howell [1] Alexandra Smith[1], Eve Roman [1,4]

[1] Department of Health Science, University of York, UK

[2] Department of paediatric Haematology, Leeds Teaching Hospital's NHS Trust, Leeds, UK

[3] CRESS, Université de Paris, INSERM UMR-1153, Epidemiology of childhood and adolescent cancers team, Villejuif, France

[4] Correspondence to: eve.roman@york. ac.uk, Epidemiology and Cancer Statistics Group, Department of Health Sciences, University of York, York, YO10 5DD, UK

Authors' names and positions

Eleanor Kane (0000-0002-7438-9982), Senior research fellow

Sally Kinsey, (0000-0002-5777-3333), Professor of paediatric haematology

Audrey Bonaventure (0000-0001-6665-8145), Cancer Researcher/Senior Visiting Research Fellow

Tom Johnston (0000-0002-5204-0966), Research fellow

Jill Simpson, (0000-0003-3156-8822), Visiting Research fellow

Debra Howell (0000-0002-7521-7402), Senior research fellow

Alexandra Smith (0000-0002-1111-966X), Reader in cancer epidemiology

Eve Roman (0000-0001-7603-3704), Professor of epidemiology

Word Count (main text) = 2492

For peer review only - http://bmjopen.bmj.com/site/about/guidelines.xhtml

Abstract

Objectives

To examine morbidity and mortality in survivors of childhood acute lymphoblastic leukaemia (ALL) across their teenage and young adult (TYA) years; comparing the patterns observed with individually matched general population controls.

Design

Case-control study with follow-up linkage to administrative healthcare databases for up to 25 years.

Setting

The study population comprises all children (0-14 years) registered for primary care with the National Health Service (NHS) in England 1992-1996.

Participants

1082 five-year survivors of ALL diagnosed <15 years of age, and 2018 age- and sex-matched population-based controls; followed to 15 March 2020.

Main outcome measures

Associations with hospital activity, cancer, and mortality were assessed using incidence rate ratios and absolute risk difference.

Results

Mortality 5-25 years after diagnosis was 20 times higher in cases than controls (Rate Ratio 21.3, 95% Confidence Interval 11.2-45.6), and cancer incidence 10 time higher (IRR 9.9 95% CI 4.1-29.1). Hospital activity was increased for many clinical specialties, the strongest effects being for endocrinology; outpatient IRR 36.7, 95% CI 17.3-93.4 and inpatient 19.7, 95% CI 1.9-25.5 for males, and 11.0, 95% CI 6.2-21.1 and 6.2 95% CI 3.1-13.5 respectively for females. Notable excesses were also evident for cardiology, neurology, ophthalmology, respiratory medicine and general medicine. Males were also more likely to attend gastroenterology, ENT (ear, nose and throat), urology, and dermatology; while females were more likely to be seen in plastic surgery and less likely in midwifery.

Conclusions

Adding to a large excess risk of death and cancer, survivors of childhood ALL experience excess outpatient and inpatient activity across their TYA years. Involving most clinical specialties, the observed effects are striking, showing no signs of diminishing over the first 25 years of follow-up. These findings underscore the need to take prior ALL drug and/or radiation treatment into account when interpreting seemingly unrelated symptoms in later life.

For peer review only - http://bmjopen.bmj.com/site/about/guidelines.xhtml

ARTICLE SUMMARY

Strengths and limitations of this study

- With minimal selection bias and loss to follow-up, findings from this national study are based on linked hospital data, not self-report
- Ensuring homogeneity of treatment, diagnostic dates are contemporaneous with the national UKALL XI RCT, within which 90.5% of those who survived were enrolled
- The individually sex- and age-matched general population controls enable robust baseline effect measures to be calculated across the 25-year follow-up period
- Analyses are constrained by the fact that hospital episodes statistics are primarily collected for administrative and clinical purposes, and not for research

Funding

This work was supported by Blood Cancer UK grant number 15037. EK and DH are supported by Cancer Research UK (grant number 29685), and AB by a fellowship from the Fondation ARC pour la Recherche sur le Cancer (PDF20190508759). The funders had no role in considering the study design or in the collection, analysis, interpretation of data, writing of the manuscript, or the decision to submit the article for publication.

Competing interests

None of the authors have any competing interests

Data sharing agreement

No additional data available

For peer review only

Introduction

Comprising around 40% of all cancers diagnosed in children before 15 years of age, acute lymphoblastic leukaemia (ALL) is the commonest paediatric malignancy in high income countries. Epitomizing one of the major therapeutic success stories of the last 50 years, five-year overall survival for childhood ALL is now around 90% in high-income settings. (1) As a result, the global prevalence of survivors is increasing year-on-year; and will continue to do so as treatments improve, populations age, costs fall, and new clinical collaborations between high- and low-income countries are forged. (2)

In contrast to many other childhood cancers, ALL treatment is prolonged, with current protocols typically administering chemotherapy in phases extending over a 2- to 3-year period. (3) That children who survive ALL tend to have higher mortality and morbidity in later life, including subsequent cancers and a wide range of other therapy-related conditions (Table 1), is established. (4–6) With respect to the latter, however, much of the information on late effects among survivors has come either from analyses of hospitalizations or from questionnaires completed by consenting individuals.(7–16) As is evident from the studies summarized in Table 1 (which includes those that examined all leukaemias combined and those that also included young adults), less attention has been paid to chronic conditions that do not require hospital admission, (17,18) and to morbidity patterns that change over time. (11,19)

The longitudinal data examined in this report contain information on hospital outpatient attendances, as well as inpatient episodes, cancer registrations, and deaths. Adding significantly to the existing knowledge base, a large cohort of children (<15 years) diagnosed with ALL in England during 1992-6 and their individually sex- and age-matched population controls have been tracked for up to 25 years through their teenage and young adult (TYA) years. Increasing the power to detect effects should they exist, this diagnostic time period (1992-6) coincides with the UKALL XI randomised controlled trial (RCT)(20); the last very intensive UK RCT prior to the era of clinical investigations enabling reduction in therapy for patients with rapid early response to treatment. (21)

Methods

Data are from the United Kingdom Childhood Cancer Study (UKCCS), a national population-based case-control study established in the 1990s to investigate potential causes of childhood cancer. Full details of the UKCCS's original methods and ethical permissions have been published. (22–24) Briefly, the study population comprised all children (0-14 years) registered for primary care with the National Health Service (NHS). All children newly diagnosed with cancer (cases) were ascertained via proactive notification systems established in all treatment centres across England, Scotland and Wales; and each child whose parents agreed to be interviewed (87%; all cancers combined) was individually matched on sex, date of birth and region of residence to up to 10 randomly selected controls. The general practitioners (GPs) of the first two controls identified ("first-choice controls") were approached and, with their permission, the parents of the children were contacted and asked to participate in the study. Overall, 72% of first-choice control families participated; but if the GP refused permission to contact the parents, or the parents themselves declined, the next control on the list was selected, and so on until two control families agreed. (22–24) For methodological

For peer review only - http://bmjopen.bmj.com/site/about/guidelines.xhtml

purposes (23,24), details of all non-participating cases and controls were retained in the UKCCS registration database.

With a view to examining childhood cancer sequelae, the UKCCS in England now operates on a legal basis that permits information to be obtained from NHS administrative healthcare records without explicit consent; and all registered cases and first-choice controls, along with participating replacement controls, are now being prospectively tracked by NHS Digital (https://digital.nhs.uk/) via linkage to nationwide (England) information on deaths, cancer registrations, and Hospital Episode Statistics (HES; inpatient admissions and outpatient attendances).

Data and statistical analysis

The present report focuses on the health of children who survived for 5-years or more following a diagnosis of ALL. The number of ALL cases diagnosed in England in the original UKCCS study and their corresponding first-choice controls, as well as deaths and losses to follow-up occurring within 5-years of diagnosis, or pseudo-diagnosis (matched controls), are shown in Figure 1. In total, 1372 children were diagnosed with ALL in England during the study period (1992 to 1996), and the parents of 92% (1262/1372) agreed to participate, each of whom had two sex-and age-matched first-choice controls selected (n=2524). Of the 1104 ALL cases (80.5%) who survived for five years or more, 1004 (90.9%) had B-ALL and 70 (6.3%) T-ALL. The cell lineage of the remaining eight ALL survivors is unknown; they were not entered into a clinical trial and the parents did not participate in the original study (hence no controls). Finally, because Down syndrome is a well-established risk factor for ALL, and is also associated with a range of other morbidities, the 22 surviving case children with Down syndrome (no first-choice control had Down syndrome) and their matched controls were excluded from the analysis.

For the 1082 cases and 2018 controls included in this report, linked healthcare data were available on all cancer registrations and deaths up to March 2020, inpatient HES from April 1997 to March 2019, and outpatient HES from April 2003 to March 2019. Data were analysed using non-parametric time-to-event analyses and exact methods to estimate hospital visit hazard rates and risks associated with specific clinical specialties (two or more outpatient visits, or at least one hospital admission). Follow-up began five years post-diagnosis and ended either at the date of death, 25 years post-diagnosis, or end date for hospital data. For controls follow-up could also end when their matched case died. Migrations out of England, any subsequent returns, as well as time spent in hospital were also accounted for. All analyses were conducted using Stata 16.1.

Patient Public Involvement and Engagement (PPIE)

The original case-control study was established over 30 years ago, and did not benefit from formal links with patient/user groups. Members of the CCLG (Children's Cancer and Leukaemia Group, previously the UK Children's Cancer Study Group) were, however, fully involved throughout. More recently with respect to the present research, the acceptability of using patient data without explicit consent (as is the case in this report) has been positively discussed with patient groups and clinicians; and the study website (www.UKCCS.org) contains information about ongoing research activities and fair processing. It is our intention to establish a PPIE group to input into future research when the COVID pandemic permits.

For peer review only - http://bmjopen.bmj.com/site/about/guidelines.xhtml

Results

The characteristics of individuals in the original case-control study and in the 5-year survivor cohorts are shown in Table 2. As expected, the 290 (21%) children with ALL who died within five years of diagnosis were more likely to have had T-cell disease (χ^2=44.0, p<0.001), have been infants or older children (χ^2=100.9, p<0.001), and were less likely to have participated in a clinical trial (χ^2=11.6, p=0.001). Tracking children from around their 9th year of age through to their 29th, this reports primary focus is teenage and young adult (TYA) survivors who, again as expected, continued to experience more deaths (cumulative mortality 10.7%, 95% Confidence Interval 9.0-12.8) than their general population counterparts (cumulative mortality 0.6%, 95% CI 0.3-1.1); yielding a mortality rate ratio of 21.3 (95% CI 11.2-45.6). Likewise, 5-year ALL survivors were also more likely to have a subsequent cancer registration (non-ALL malignancy Incidence Rate Ratio 9.9, 95% CI 4.1-29.1), although the numbers are small (32 *versus* 6).

Childhood ALL survivors are regularly monitored throughout their TYA years either in paediatrics, haematology or oncology, as is evidenced in Figure 2A which shows the outpatient activity of 5-year ALL survivors and their corresponding controls for these three clinical specialties combined (specialty-specific outpatient and inpatient data are shown in Supplementary Figure 1). At around one visit in 20 years, hospital activity among population controls is very low, and varies little over time. By contrast, for ALL survivors, 8 years after diagnosis outpatient activity is around 35 times higher than the background rate, falling 10 years later to around 0.4 visits per case per year (Figure 2A). In all other clinical specialties combined, outpatient activity among childhood ALL survivors are also consistently higher than expected; hazard rates in both cohorts increasing over time at the same steady rate (Figure 2B).

More information on hospital activity is presented for the top 15 specialties (excluding paediatrics, haematology and oncology) for males and females separately in Figure 3 (outpatients) and Figure 4 (inpatients). In all figures, cumulative incidence frequencies (outpatient = two or more face-to-face visits to that specialty; inpatient = any admission) are on the left and incidence rates on the right; with the ordering of specialties determined by the magnitude of the incidence rate ratio (IRR). Most specialties are associated with high relative and attributable risks (inpatient and outpatient). Furthermore, although the magnitude of the effect often varies between males and females, the overall pattern is broadly similar. In both sexes, for example, the strongest effects are seen for endocrinology; the IRRs for outpatients and inpatients being 36.7 (95% CI 17.3-93.4) and 19.7 (95% CI 7.9-63.2) respectively for males, and 11.0 (95% CI 6.2-21.1) and 6.2 (95% CI 3.1-13.5) for females. Notable excesses (relative and attributable) are also evident for cardiology, neurology, ophthalmology, respiratory medicine and general medicine, for both genders. Male survivors were also more likely than their peers to attend gastroenterology, ENT (ear, nose and throat), urology, and dermatology; while female survivors were twice as likely as their controls to be referred to plastic surgery. Interestingly, no case-control differences are evident for trauma and orthopaedics, which has the highest background cumulative incidence in males. Likewise, among females, no differences were detected for obstetrics and gynaecology; however, it is notable that activity is lower than expected in midwifery (outpatient IRR 0.9, 95% CI 0.7-1.2; inpatient IRR 0.6, 95% CI 0.4-0.9).

Detailed inspection of the HES data revealed no evidence of specialty-specific changes in either relative or attributable risks over the 20-year time frame (Supplementary Figure 2); possible exceptions being falls in ranking for ophthalmology among males and dermatology among females.

For peer review only - http://bmjopen.bmj.com/site/about/guidelines.xhtml

Furthermore, no differences between B- and T-cell survivors with respect to any of the outcomes were evident, although the number with T-cell disease was comparatively small (n=70).

Discussion

Including data on more than 1000 survivors of childhood ALL and twice as many age- and sex-matched general population controls, this national record-linkage study found that survivors continued to experience large excesses in mortality and morbidity throughout their teenage and young adult years. With hospital activity being higher than expected in specialties covering most organ and tissue systems, survivors were more than twice as likely to fall under the care of endocrinology, cardiology, respiratory medicine, ophthalmology, neurology and/or gastroenterology specialists. Moreover, aside from a gradual decline in visits to clinical specialties involved in follow-up monitoring (paediatrics, haematology and oncology), there was little indication of activity returning to general population levels up to 25 years after the initial ALL diagnosis. Among females, it is also notable that midwifery outpatient attendances were not increased, with admissions being lower than expected.

Our study, which uses linked rather than self-reported data and population controls, is one of only two (17) to separate secondary care activity associated with cancer follow-up from activity associated with other morbidities; finding that survivors not only experienced higher rates of admission but also higher rates of outpatient visits, neither of which abated over their TYA years. Reported less often are the types of conditions underlying these associations, with four studies assessing risks by organ system (8,12,14,17). Survivors in these studies were diagnosed over a wider period than the study reported here, and had median follow-up times that were shorter; 9 to 17 years from diagnosis, compared to 25 years here. These studies found that survivors were at increased risk of being hospitalised with endocrine or infectious diseases, as well as diseases involving the nervous, circulatory, skin, digestive, genitourinary, respiratory or musculoskeletal systems. With the information source mostly being inpatient records, reports using data from other healthcare settings are sparse (17) and not specific to survivors of ALL (18). Importantly, while our findings are broadly consistent with these studies, our methods also highlight excesses in ophthalmology, ENT (ear, nose and throat), and oral/dental specialties, as well as decreases in midwifery.

Ensuring homogeneity of treatment, the original study's diagnostic dates were contemporaneous with the national UKALL XI randomized controlled trial (RCT), within which 90.5% of UKCCS 5-year survivors were enrolled (20,25). This comparatively high-intensity RCT was the last UK childhood ALL trial prior to the stratification of patient treatment based on early bone marrow response to induction treatment (measured by percentage of leukaemia cells in the marrow) (21,26). Information on UKALL XI's scheduling of drugs is provided in the report by Hann and colleagues (20), several of which have been implicated, either singly or combined, in therapy-related effects (Table 1). It is, however, important to note that several of the UKALL XI drugs are no longer used (e.g. etoposide, 6-thioguanine) or are used more sparingly (e.g. daunorubicin, cyclophosphamide, corticosteroids), and that cranial irradiation is no longer routinely administered, being reserved for infrequent particular indications (27). Hence, the nature of the UKCCS cohort means that it is well placed to provide baseline data against which to evaluate the impact of treatment de-escalation and other protocol modifications. Other major strengths include its national coverage, completeness of case ascertainment, and large in-built general population comparator (22–24); the latter obviating the need to rely on published national rates, as has been the case in the UK thus far (9,14,28–30).

For peer review only - http://bmjopen.bmj.com/site/about/guidelines.xhtml

For peer review only

With regard to weaknesses, the lack of data from other parts of the NHS, including primary care and adolescent psychosocial services, is an obvious deficiency that currently affects all UK record-linkage studies of the type described here. The relative paucity of information on psychological morbidities is particularly relevant to childhood cancer survivors, who are known to be at increased risk of a number of such disorders (4,31,32). Missing information within the HES datasets is also an issue; diagnostic data, for example, is largely absent from outpatient HES. Furthermore, although steps to mitigate surveillance bias were taken by requiring at least two face-to-face visits to a given specialty, we cannot rule out the possibility that childhood cancer survivors were more likely to be referred to secondary care than their peers.

Adding to a large excess risk for death and cancer, survivors of childhood ALL also experience excess outpatient and inpatient activity across their teenage and young adult years. Involving most clinical specialties, the observed effects are striking; showing no signs of diminishing over the first 25 years of follow-up. The main exception is the comparatively low midwifery contact seen among female ALL survivors. These findings underscore the important need to take prior ALL drug and or radiation treatments into account when interpreting seemingly unrelated symptoms in later life. With virtually complete linkage to existing national databases established, our future research will continue to monitor the health of cancer survivors across their life-span.

Ethical approval: The United Kingdom Childhood Cancer Study (UKCCS) has ethics approval from the Yorkshire & The Humber-Leeds West Research Ethics Committee (reference 18/YH/0135) and exemption from Section 251 of the Health & Social Care Act (reference 18/CAG/0066).

Contributors: ER, SK, and JS contributed to the design of the original study; and ER and EK instigated the present investigation and prepared the report. EK conducted the analyses, and all authors (ER, EK, SK, AB, TJ, JS, DH, AS) were involved in the development, reviewing, and approving the final manuscript. ER is the principal investigator and guarantor. The corresponding author (ER) attests that all listed authors meet the authorship criteria and that no others meeting the criteria have been omitted.

For peer review only - http://bmjopen.bmj.com/site/about/guidelines.xhtml

References

1. Bonaventure A, Harewood R, Stiller CA, Gatta G, Clavel J, Stefan DC, et al. Worldwide comparison of survival from childhood leukaemia for 1995–2009, by subtype, age, and sex (CONCORD-2): a population-based study of individual data for 89 828 children from 198 registries in 53 countries. The Lancet Haematology. 2017 May 1;4(5):e202–17.

2. The European Society for Paediatric Oncology. The SIOPE Strategic Plan. A European Cancer Plan for Children and Adolescents. [Internet]. SIOP Europe; 2015 Sep. Available from: https://siope.eu/european-strategic-plan/

3. Teachey DT, Hunger SP, Loh ML. Optimizing therapy in the modern age: differences in length of maintenance therapy in acute lymphoblastic leukemia. Blood. 2021 Jan 14;137(2):168–77.

4. Chow EJ, Ness KK, Armstrong GT, Bhakta N, Yeh JM, Bhatia S, et al. Current and coming challenges in the management of the survivorship population. Semin Oncol. 2020;47(1):23–39.

5. Landier W, Skinner R, Wallace WH, Hjorth L, Mulder RL, Wong FL, et al. Surveillance for Late Effects in Childhood Cancer Survivors. J Clin Oncol. 2018 Jul 20;36(21):2216–22.

6. Norsker FN, Pedersen C, Armstrong GT, Robison LL, McBride ML, Hawkins M, et al. Late Effects in Childhood Cancer Survivors: Early Studies, Survivor Cohorts, and Significant Contributions to the Field of Late Effects. Pediatric Clinics of North America. 2020 Dec 1;67(6):1033–49.

7. Dixon SB, Chen Y, Yasui Y, Pui C-H, Hunger SP, Silverman LB, et al. Reduced Morbidity and Mortality in Survivors of Childhood Acute Lymphoblastic Leukemia: A Report From the Childhood Cancer Survivor Study. JCO. 2020 Jul 24;JCO.20.00493.

8. Sørensen GV, Winther JF, de Fine Licht S, Andersen KK, Holmqvist AS, Madanat-Harjuoja L, et al. Long-term risk of hospitalization among five-year survivors of childhood leukemia in the Nordic countries. J Natl Cancer Inst [Internet]. 2019 Feb 11; Available from: https://www.ncbi.nlm.nih.gov/pmc/articles/PMC6748786/pdf/djz016.pdf

9. Reulen RC, Guha J, Bright CJ, Henson KE, Feltbower RG, Hall M, et al. Risk of cerebrovascular disease among 13 457 five-year survivors of childhood cancer: A population-based cohort study. International Journal of Cancer. 2021;148(3):572–83.

10. Streefkerk N, Tissing WJE, Korevaar JC, van Dulmen-den Broeder E, Bresters D, van der Heiden-van der Loo M, et al. A detailed insight in the high risks of hospitalizations in long-term childhood cancer survivors-A Dutch LATER linkage study. PLoS ONE. 2020;15(5):e0232708.

11. Mulrooney DA, Hyun G, Ness KK, Bhakta N, Pui C-H, Ehrhardt MJ, et al. The changing burden of long-term health outcomes in survivors of childhood acute lymphoblastic leukaemia: a retrospective analysis of the St Jude Lifetime Cohort Study. Lancet Haematol. 2019 Jun;6(6):e306–16.

12. Mueller BA, Doody DR, Weiss NS, Chow EJ. Hospitalization and mortality among pediatric cancer survivors: a population-based study. Cancer Causes Control. 2018 Nov;29(11):1047–57.

13. Ou JY, Smits-Seemann RR, Kaul S, Fluchel MN, Sweeney C, Kirchhoff AC. Risk of hospitalization among survivors of childhood and adolescent acute lymphoblastic leukemia compared to siblings and a general population sample. Cancer Epidemiol. 2017;49:216–24.

For peer review only - http://bmjopen.bmj.com/site/about/guidelines.xhtml

14. Brewster DH, Clark D, Hopkins L, Bauer J, Wild SH, Edgar AB, et al. Subsequent hospitalisation experience of 5-year survivors of childhood, adolescent, and young adult cancer in Scotland: a population based, retrospective cohort study. Br J Cancer. 2014 Mar 4;110(5):1342–50.

15. Oudin C, Berbis J, Bertrand Y, Vercasson C, Thomas F, Chastagner P, et al. Prevalence and characteristics of metabolic syndrome in adults from the French childhood leukemia survivors' cohort: a comparison with controls from the French population. Haematologica. 2018 Apr;103(4):645–54.

16. Hau EM, Caccia JN, Kasteler R, Spycher B, Suter T, Ammann RA, et al. Cardiovascular disease after childhood acute lymphoblastic leukaemia: a cohort study. Swiss Medical Weekly [Internet]. 2019 Mar 10 [cited 2020 Oct 17];149(0910). Available from: https://smw.ch/article/doi/smw.2019.20012

17. Holmqvist AS, Moëll C, Hjorth L, Lindgren A, Garwicz S, Wiebe T, et al. Increased health care utilization by survivors of childhood lymphoblastic leukemia is confined to those treated with cranial or total body irradiation: a case cohort study. BMC Cancer. 2014 Jun 10;14:419.

18. McBride ML, Lorenzi MF, Page J, Broemeling A-M, Spinelli JJ, Goddard K, et al. Patterns of physician follow-up among young cancer survivors: report of the Childhood, Adolescent, and Young Adult Cancer Survivors (CAYACS) research program. Can Fam Physician. 2011 Dec;57(12):e482-490.

19. Gibson TM, Mostoufi-Moab S, Stratton KL, Leisenring WM, Barnea D, Chow EJ, et al. Temporal patterns in the risk of chronic health conditions in survivors of childhood cancer diagnosed 1970-99: a report from the Childhood Cancer Survivor Study cohort. Lancet Oncol. 2018;19(12):1590–601.

20. Hann I, Vora A, Richards S, Hill F, Gibson B, Lilleyman J, et al. Benefit of intensified treatment for all children with acute lymphoblastic leukaemia: results from MRC UKALL XI and MRC ALL97 randomised trials. UK Medical Research Council's Working Party on Childhood Leukaemia. Leukemia. 2000 Mar;14(3):356–63.

21. Mitchell C, Payne J, Wade R, Vora A, Kinsey S, Richards S, et al. The impact of risk stratification by early bone-marrow response in childhood lymphoblastic leukaemia: results from the United Kingdom Medical Research Council trial ALL97 and ALL97/99. British Journal of Haematology. 2009;146(4):424–36.

22. Johnston WT, Lightfoot TJ, Simpson J, Roman E. Childhood cancer survival: a report from the United Kingdom Childhood Cancer Study. Cancer Epidemiol. 2010 Dec;34(6):659–66.

23. UK Childhood Cancer Study Investigators. The United Kingdom Childhood Cancer Study: objectives, materials and methods. Br J Cancer. 2000 Feb 11;82(5):1073–102.

24. Smith A, Roman E, Simpson J, Ansell P, Fear NT, Eden T. Childhood leukaemia and socioeconomic status: fact or artefact? A report from the United Kingdom childhood cancer study (UKCCS). International Journal of Epidemiology. 2006 Dec 1;35(6):1504–13.

25. Chessells JM, Harrison CJ, Watson SL, Vora AJ, Richards SM. Treatment of infants with lymphoblastic leukaemia: results of the UK Infant Protocols 1987–1999. British Journal of Haematology. 2002;117(2):306–14.

For peer review only - http://bmjopen.bmj.com/site/about/guidelines.xhtml

26. Inaba H, Pui C-H. Advances in the Diagnosis and Treatment of Pediatric Acute Lymphoblastic Leukemia. Journal of Clinical Medicine. 2021 Jan;10(9):1926.

27. Brown P, Inaba H, Annesley C, Beck J, Colace S, Dallas M, et al. Pediatric Acute Lymphoblastic Leukemia, Version 2.2020, NCCN Clinical Practice Guidelines in Oncology. J Natl Compr Canc Netw. 2020 Jan;18(1):81–112.

28. Smith L, Glaser AW, Peckham D, Greenwood DC, Feltbower RG. Respiratory morbidity in young people surviving cancer: Population-based study of hospital admissions, treatment-related risk factors and subsequent mortality. Int J Cancer. 2019 01;145(1):20–8.

29. Reulen RC, Frobisher C, Winter DL, Kelly J, Lancashire ER, Stiller CA, et al. Long-term risks of subsequent primary neoplasms among survivors of childhood cancer. JAMA. 2011 Jun 8;305(22):2311–9.

30. Fidler MM, Reulen RC, Winter DL, Kelly J, Jenkinson HC, Skinner R, et al. Long term cause specific mortality among 34 489 five year survivors of childhood cancer in Great Britain: population based cohort study. BMJ. 2016 Sep 1;354:i4351.

31. Andrés-Jensen L, Skipper MT, Mielke Christensen K, Hedegaard Johnsen P, Aagaard Myhr K, Kaj Fridh M, et al. National, clinical cohort study of late effects among survivors of acute lymphoblastic leukaemia: the ALL-STAR study protocol. BMJ Open [Internet]. 2021 Feb 9 [cited 2021 May 4];11(2). Available from: https://www.ncbi.nlm.nih.gov/pmc/articles/PMC7875271/

32. van Erp LME, Maurice-Stam H, Kremer LCM, Tissing WJE, van der Pal HJH, de Vries ACH, et al. A vulnerable age group: the impact of cancer on the psychosocial well-being of young adult childhood cancer survivors. Support Care Cancer. 2021 Aug;29(8):4751–61.

For peer review only

For peer review only - http://bmjopen.bmj.com/site/about/guidelines.xhtml

Legends

Table 1: Studies reporting morbidity among 5-year survivors of leukaemia diagnosed in children, teenagers, and young adults; both acute lymphoblastic leukaemia (ALL) alone, and all leukaemias combined

Table 2: Characteristics of acute lymphoblastic leukaemia (ALL) cases diagnosed <15 years in England 1992-96 and their age- and sex-matched first-choice controls[1]: total registered in the original case-control study, and 5-year survivors included in the present study

Figure 1: Flowchart showing subjects targeted in the original United Kingdom Childhood Cancer case-control study and those included in the present cohort

Figure 2: Outpatient activity hazard rates per year and 95% confidence intervals (dotted lines) for childhood (< 15 years) ALL 5-year survivors and their matched controls: A) paediatrics, haematology and oncology, B) all other clinical specialties

Figure 3: Cumulative incidence (%), incidence rates (per 1000 person-years), attributable risks, and incidence rate ratios for the top 15 outpatient specialties (excluding paediatrics, haematology and oncology) with two of more face-to-face speciality-specific visits in the 5 to 25 years following diagnosis (cases diagnosed <15 years, 1992-96) and their matched population controls

Figure 4: Cumulative incidence (%), incidence rates (per 1000 person-years) and incidence rate ratios for the top 15 inpatient specialties (excluding paediatrics, haematology and oncology) in the 5 to 25 years following diagnosis (cases diagnosed aged<15 years, 1992-96) and their matched population controls.

Supplementary Figure 1: Hazard rates per year of activity at paediatrics, haematology and oncology among ALL cases who survived 5 years or more and their matched population controls: UKCCS, ALL diagnosed aged<15 years, 1992-96.

Supplementary Figure 2: Cumulative incidence (%), incidence rates (per 1000 person-years), attributable risks and incidence rate ratios for the top 15 outpatient specialties (excluding paediatrics, haematology and oncology) with two of more face-to-face speciality-specific visits in the 5 to 15 years and 15 to 25 years following diagnosis (cases diagnosed <15 years, 1992-96) and their matched population controls.

For peer review only

Table 1: Studies reporting morbidity among 5-year survivors of leukaemia diagnosed in children, teenagers, and young adults; both acute lymphoblastic leukaemia (ALL) alone, and all leukaemias combined

Study	5-year survivors: Setting, Timeframe	5-year survivors: Numbers, Completeness	Comparator Source	Follow-up[1] Source, Timing post-diagnosis	Outcome Measures	Main Findings
**Acute lymphoblastic leukaemia**						
Childhood Cancer Survivor Study, CCSS (7)	USA & Canada: 31 centres<br><br>1970-1999	6148 <21yrs<br><br>~72% consented at baseline	5051 controls<br><br>Closest aged siblings in random sample of all childhood cancer survivors	Biannual questionnaires 2000-2017<br><br>5-46yrs post-diagnosis (median 22yrs)	Conditions considered severe by the respondents, distributed by treatment era and treatment group	11% of survivors diagnosed in the 1990s receiving standard treatment reported at least one severe chronic condition in the 20yr after diagnosis; Rate Ratio 1.9 (95%CI 1.5-2.3) compared to controls. RRs>2 for stroke, major joint replacement, and diabetes
Adult Life after Childhood Cancer in Scandinavia, ALiCCS[2] (8)	Denmark, Sweden, Iceland & Finland: National cancer registries<br><br>1970-2008[2]	3391 <20yrs<br><br>100% traced	129 828 controls matched on sex, age, and country<br><br>National population registers	National hospital registers 1975-2012<br><br>5-42yrs post-diagnosis (median 16yrs)	First inpatient admission for 120 conditions, total and by organ system	Survivors were nearly twice (Rate Ratio=1.95, 95%CI 1.83-2.07) as likely to be hospitalised for ≥1 of 120 conditions than controls. RRs>2 for diseases of the endocrine, nervous, and circulatory systems; infections, blood/blood organs, second cancers, and benign neoplasms
St Jude Lifetime Cohort, SJLIFE (11)	USA: St Jude Children's Research Hospital<br><br>1963-2003	980 <18yrs alive 10 years after diagnosis<br><br>~67% consented	272 controls<br><br>Friends, non-first-degree relatives of hospital patients, hospital employees and volunteers	In-person interview 2015<br><br>12-52yrs post-diagnosis (median 25yrs)	Conditions considered by respondents to be moderate/severe, overall, and by organ system	By age 30, survivors reported 3.2 (95%CI 2.9-3.4) chronic conditions compared with 1.2 (95%CI 1.0-1.4) among controls. Conditions varied by treatment era, with endocrine and musculoskeletal conditions contributing more to the cumulative burden in the 1990s and 2000s than in earlier decades
Swiss Childhood Cancer Survivor Study, SCCSS (16)	Switzerland: Swiss Childhood Cancer Registry<br><br>1976-2005	511 <16yrs, alive in 2007 and aged≥16yrs at questionnaire<br><br>72% participated	702 siblings	Questionnaire 2007-2012<br><br>5- 36yrs post-diagnosis	Self-reported cardiovascular diseases	14% of survivors reported having at least one cardiovascular disease, nearly twice that of their siblings (Odds Ratio=1.9, 95%CI 1.3-2.8)

For peer review only - http://bmjopen.bmj.com/site/about/guidelines.xhtml

Study	5-year survivors		Comparator Source	Follow-up[1] Source, Timing post-diagnosis	Outcome Measures	Main Findings
	Setting, Timeframe	Numbers, Completeness				
Childhood, adolescent and young adult cancer survivors, CAYACS (18)	Canada: British Columbia cancer registry[4]<br><br>1970-1992	242 <20yrs alive at 31 December 2000<br><br>~64% traced	11 570 controls frequency-matched on age and sex<br><br>Health insurance plan	Health insurance claims 1998-2000<br><br>8-20yrs post-diagnosis	Physician visits over three-year period excluding oncology, overall and by specialty	Survivors were nearly twice as likely as controls to visit a doctor at least once (Rate Ratio=1.95, 95%CI 1.8-2.1). RRs≥2 were found for ≥1 visit to neurology, ophthalmology, otolaryngology, internal medicine, surgery, urology, psychiatry, and dermatology
Childhood Cancer Register of Southern Sweden, BORISS (17)	Sweden: Regional cancer registry<br><br>1970-1999	213 <18yrs<br><br>~100% traced	10 650 controls matched on sex, year of birth and county<br><br>National population register	National hospital registers inpatient: 1975-2009, outpatient: 2001-2009<br><br>5-33yrs post-diagnosis (median 16yrs)	Hospital contacts (in/outpatient), overall and for 20 disease categories	Survivors were over twice as likely to have ≥1 hospital contact than controls (Odds Ratio=2.5, 95%CI 1.5-4.3), and were more than twice as likely to consult for disorders of the blood, endocrine system, nervous system, skin, infections or benign neoplasms
University of Utah-Intermountain Healthcare (13)	USA: Primary Children's Hospital, Salt Lake City<br><br>1998-2008	176 <22yrs<br><br>~79% traced	503 controls matched on sex and year of birth<br><br>State population register	State hospital registers 2003-2013<br><br>5-10yrs post-diagnosis (median 9yrs)	Rate of inpatient admissions over a maximum of five years	In the 5 to 10 years after diagnosis, the hospitalisation rate of survivors was 3.76 per 100 person-years (95%CI 2.22-6.36), twice the rate among controls (Relative Hospitalisation Rate=2.01, 95%CI 1.00-4.03)
**All leukaemias combined**						
British Childhood Cancer Survivor Study, BCCSS (9)	UK: National Registry of Childhood Tumours<br><br>1940-1991	3544 <15yrs, alive in April 1997<br><br>~87% being followed	Hospitalisation rates for cerebrovascular disease<br><br>National population and hospital registers	National hospital registers 1997-2012<br><br>5-72yrs post-diagnosis	Cerebrovascular disease recorded in hospital discharge summaries	Survivors were almost five times more likely to have been hospitalised for cerebrovascular disease than expected (Age Standardised Hospitalisation Ratio=4.7, 95%CI 3.6-6.1)
Dutch Childhood Oncology Group-LATER Study (10)	Netherlands: Paediatric oncology or haematology centres<br><br>1963-2001	1900 <18yrs, alive in January 1995<br><br>~92% traced	38 000 controls matched on sex and year of birth<br><br>National population register	National hospital registers 1995-2015<br><br>5-52yrs post-diagnosis	Rate of inpatient admissions over a maximum of ten years	The hospitalisation rate of survivors was almost twice that of controls (Relative Hospitalisation Rate=1.87, 95%CI 1.65-2.12)

For peer review only - http://bmjopen.bmj.com/site/about/guidelines.xhtml

Study	5-year survivors		Comparator Source	Follow-up[1] Source, Timing post-diagnosis	Outcome Measures	Main Findings
	Setting, Timeframe	Numbers, Completeness				
Leucémies de l'Enfant et l'Adolescent Cohort, LEA (15)	France: 16 cancer centres<br><br>1980-2012	1025 <18yrs, alive in 2007 and aged≥18yrs at last LEA medical examination<br><br>72% participated	3203 controls matched on sex and age<br><br>Volunteers for free medical examinations	Medical examinations at 2yr intervals until 10 years post-diagnosis (or age 20), thereafter every 4yrs<br><br>2-34yrs post-diagnosis (mean 16yrs)	Metabolic syndrome found at medical examination	Prevalence of metabolic syndrome among survivors was 10.3%, more than twice that among controls (Odds Ratio=2.49, 95%CI 1.91-2.35)
Scottish Cancer Registry (14)	UK: Scottish cancer registry<br><br>1981-2003	884 <25yrs<br><br>~100% traced	Hospitalisation rates<br><br>National population and hospital registers	National hospital registers 1986-2009<br><br>5-28yrs post-diagnosis (mean 16syrs)	First inpatient admission for particular diseases, overall, and for selected disease categories	Survivors were almost four times more likely to have been hospitalised than expected (Standardised Hospitalisation Ratio=3.9, 95%CI 3.6-4.3). SHR≥2 for diseases of the endocrine, nervous, circulatory, respiratory and digestive systems; infections and neoplasms
Washington State Cancer Registry (12)	USA: Washington State cancer registry<br><br>1982-2008	815 <20yrs<br><br>Not evaluable	8150 controls matched on sex and year of birth and alive at case's diagnosis<br><br>State birth records	State hospital registers 1987-2013<br><br>5-27yrs post-diagnosis (median 9yrs)	Rates of inpatient admissions, and hospitalisation or death for selected disease categories	The survivors' hospitalisation rate was almost three times that of controls (Hazard Ratio=2.8, 95%CI 2.2-3.5). HRs≥2 were observed for hospitalisation or death from endocrine, nervous, circulatory, respiratory, digestive, genitourinary, skin and musculoskeletal systems; blood diseases infections and cancers

[1]Minimum to maximum possible follow-up relative to diagnosis date.[2]Finland 1969-2012; Denmark 1977-2010; Sweden 1987-2009 with some regions starting in 1964-1987; Iceland 1999-2008. [3]Completeness could not be evaluated in the Washington State Cancer Registry Study as study subjects were not linked to a population register. [4]CAYACS reported outcomes for all childhood cancer survivors, of which 20% had AL

For peer review only - http://bmjopen.bmj.com/site/about/guidelines.xhtml

Table 2: Characteristics of acute lymphoblastic leukaemia (ALL) cases diagnosed <15 years in England 1992-96 and their age- and sex-matched first-choice controls[1]: total registered in the original case-control study, and 5-year survivors included in the present study

	**Original study**		**5-year survivors**	
	**Cases** N (%)	**Controls[1]** N (%)	**Cases** N (%)	**Controls[1]** N (%)
**Total**	1372 (100)	2524 (100)	1082 (100)	2018 (100)
**Sex**				
Male	763 (55.6)	1410 (55.9)	589 (54.4)	1099 (54.5)
Female	609 (44.4)	1114 (44.1)	493 (45.6)	919 (45.5)
**Lineage[1]**	-	-		
B-ALL	1234 (89.9)	2282 (90.4)	1004 (92.8)	1878 (93.1)
T-ALL	120 (8.7)	240 (9.5)	70 (6.5)	140 (6.9)
**Age (years) at:**				
Diagnosis				
<1	52 (3.8)	86 (3.4)	22 (2.0)	41(2.0)
1-4	839 (61.2)	1578 (62.5)	703 (65.0)	1331 (66.0)
5-9	267 (19.5)	484 (19.2)	220 (20.3)	402 (19.9)
10-14	214 (15.6)	376 (14.9)	137 (12.7)	244 (12.1)
Median (IQR)	4.5 (3.0-7.6)	4.4 (3.0-7.5)	4.4 (3.0-7.1)	4.3 (3.0-7.0)
Beginning of follow-up				
Median (IQR)	-	-	9.4 (8.0-12.1)	9.3 (8.0-12.0)
End of follow-up[2]				
Median (IQR)	-	-	29.6 (27.3-32.1)	29.5 (27.0-31.9)
**IMD[3] quintile at diagnosis**				
Least deprived (1-3)	834 (60.8)	1533 (60.7)	675 (62.4)	1237 (61.3)
Most deprived (4-5)	520 (37.9)	975 (38.6)	400 (37.0)	766 (38.0)
**Trial entry**				
Yes	1215 (88.6)	-	979 (90.5)	-
No	157 (11.4)	-	103 (9.5)	-
**Deaths in follow-up**	-	-	115	10
Cumulative mortality, % (95%CI)			10.7 (9.0-12.8)	0.6 (0.3-1.1)
Mortality Rate Ratio (95%CI)			21.3 (11.2-45.6)	
**Cancer[4] registrations in follow-up**	-	-		
Total			60	23
Cumulative incidence, % (95%CI)			5.8 (4.5-7.4)	1.4 (0.9-2.0)
Incidence Rate Ratio (95%CI)			4.9 (3.0-8.9)	
Malignancies only			32	6
Cumulative incidence, % (95%CI)			3.1 (2.2-4.3)	0.4 (0.1-0.8)
Incidence Rate Ratio (95%CI)			9.9 (4.1-29.1)	

[1]Lineage was not confirmed for 18 cases, 8 of whom were alive 5 years after diagnosis.
[2]Follow-up time among controls was censored at the matched survivor's death if the survivor died during follow-up. IQR: interquartile range.
[3]IMD: Index of Multiple Deprivation income domain, IMD was missing for 18 cases and 16 controls.
[4]Total cancer, including tumours that were registered as benign, in situ and of uncertain behaviour: ICD-10 codes C00-C90, C92-C97 and D00-D48. Malignancies only: C00-C43, C45-C90, C92-C97 (NB registrations for lymphoid leukaemia (C91) were excluded). Cumulative incidence of cancer was calculated considering death as a competing risk.

For peer review only

For peer review only - http://bmjopen.bmj.com/site/about/guidelines.xhtml

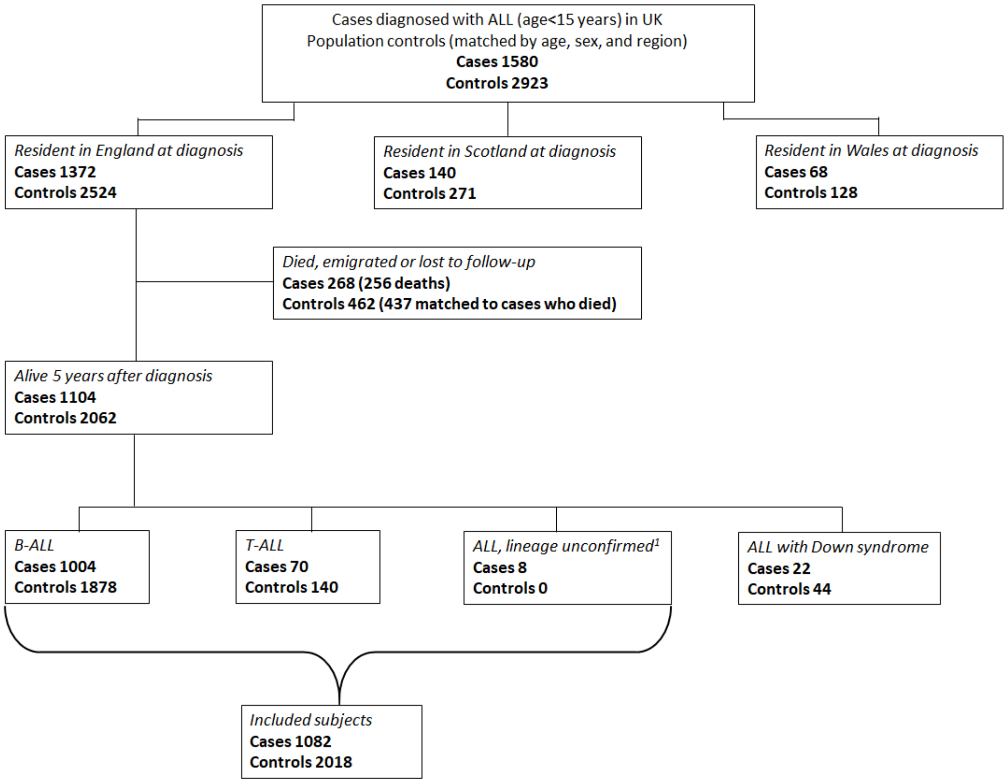

Figure 1: Flowchart showing subjects targeted in the original United Kingdom Childhood Cancer case-control study and those included in the present cohort

715x558mm (38 x 38 DPI)

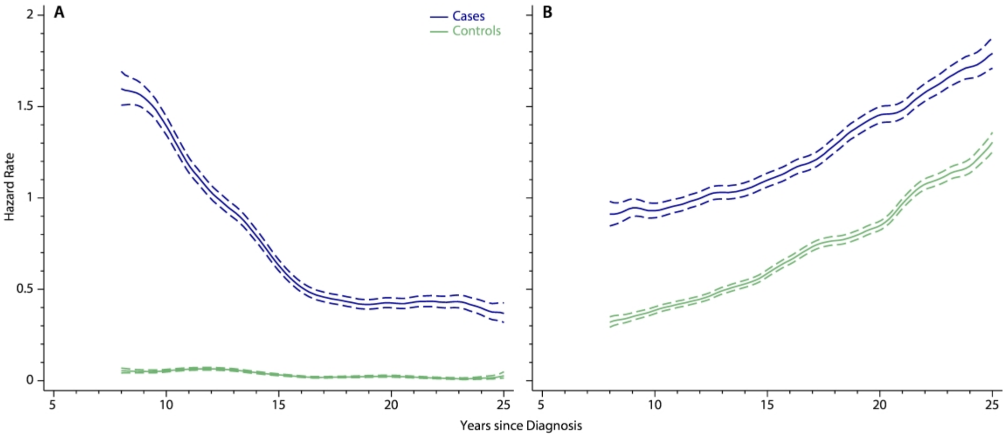

Figure 2: Outpatient activity hazard rates per year and 95% confidence intervals (dotted lines) for childhood (< 15 years) ALL 5-year survivors and their matched controls: A) paediatrics, haematology and oncology, B) all other clinical specialties

715x309mm (236 x 236 DPI)

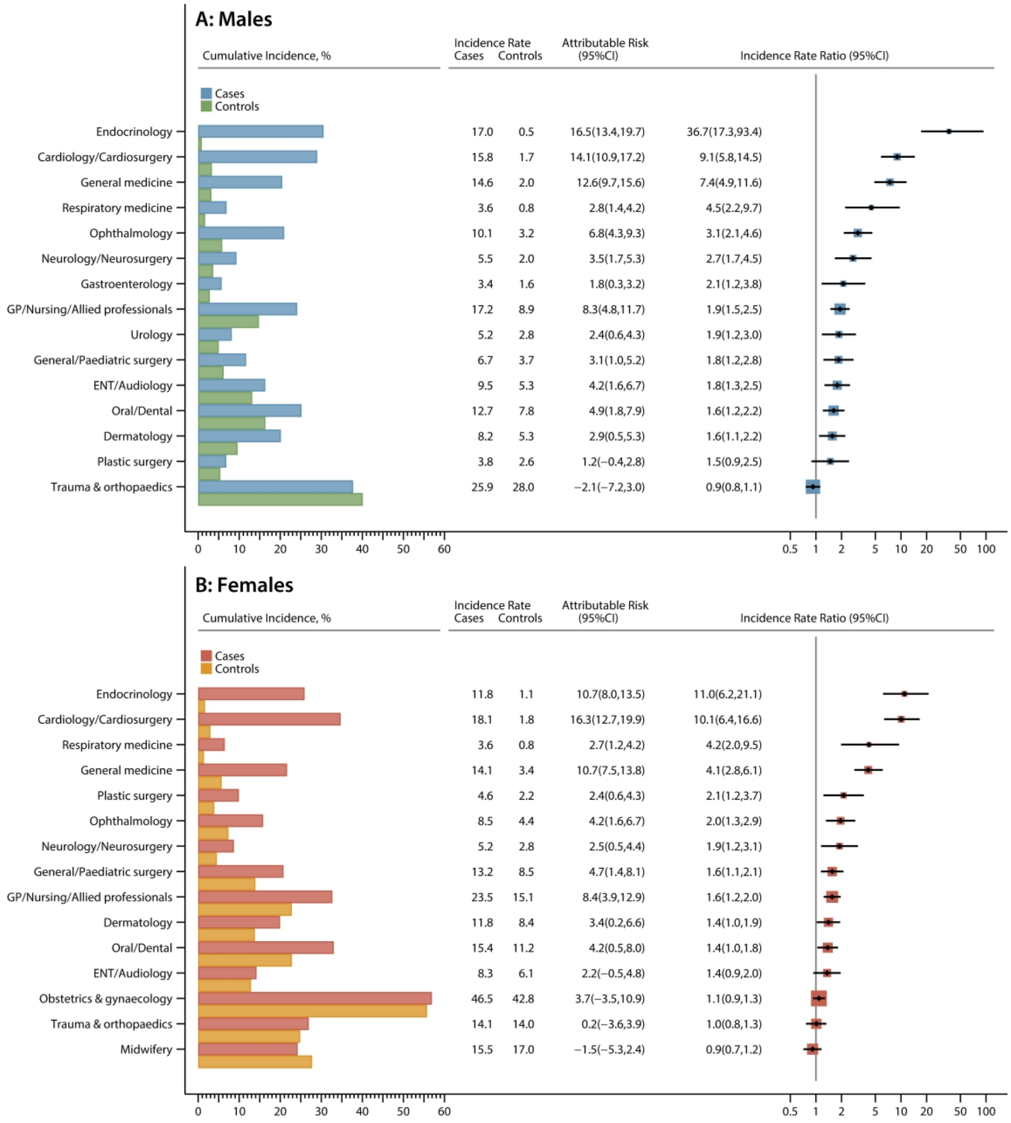

Figure 3: Cumulative incidence (%), incidence rates (per 1000 person-years), attributable risks, and incidence rate ratios for the top 15 outpatient specialties (excluding paediatrics, haematology and oncology) with two of more face-to-face speciality-specific visits in the 5 to 25 years following diagnosis (cases diagnosed <15 years, 1992-96) and their matched population controls

380x423mm (600 x 600 DPI)

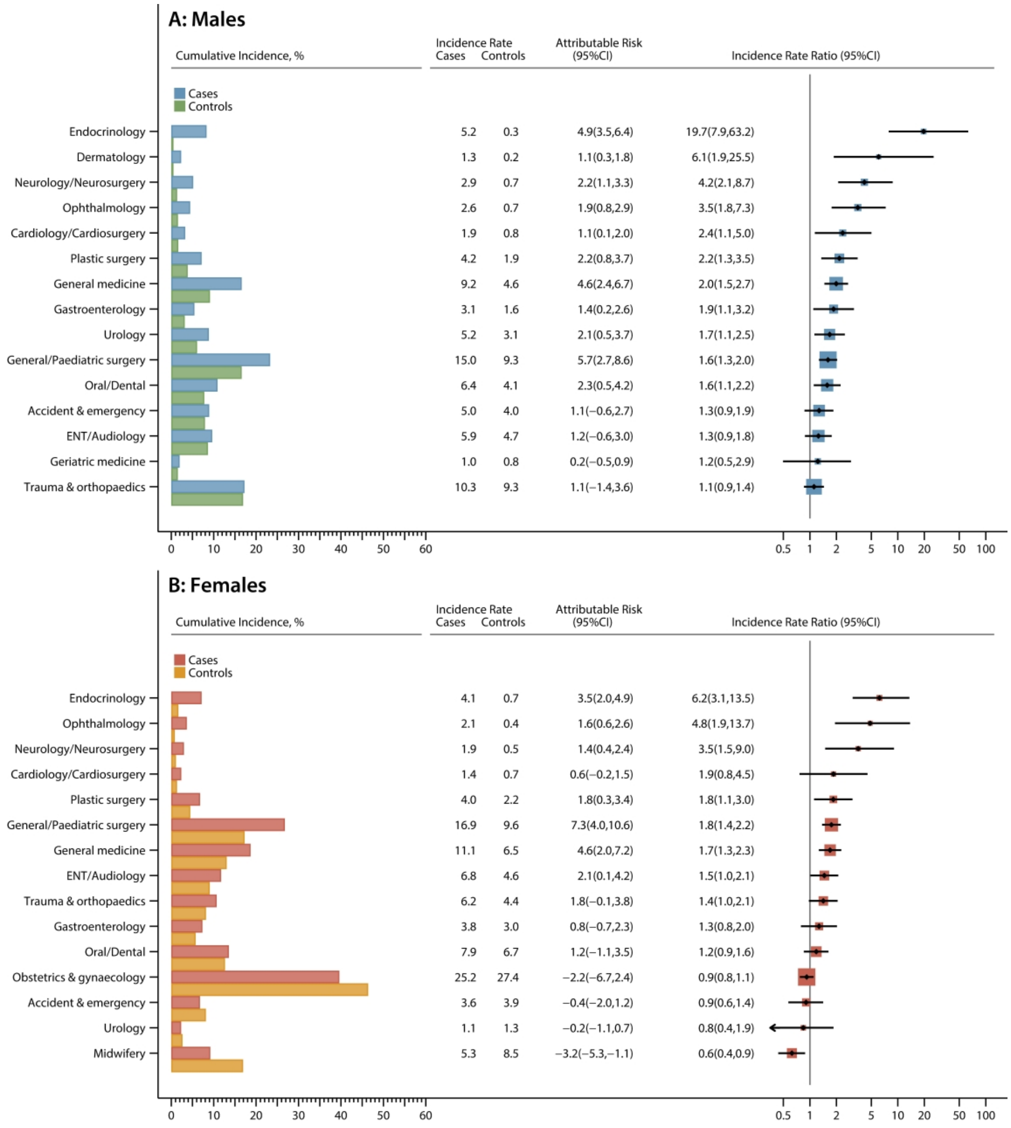

Figure 4: Cumulative incidence (%), incidence rates (per 1000 person-years) and incidence rate ratios for the top 15 inpatient specialties (excluding paediatrics, haematology and oncology) in the 5 to 25 years following diagnosis (cases diagnosed aged<15 years, 1992-96) and their matched population controls.

380x425mm (600 x 600 DPI)

Supplementary Figure 1: Hazard rates per year of activity at paediatrics, haematology and oncology among ALL cases who survived 5 years or more and their matched population controls: UKCCS, ALL diagnosed aged<15 years, 1992-96.

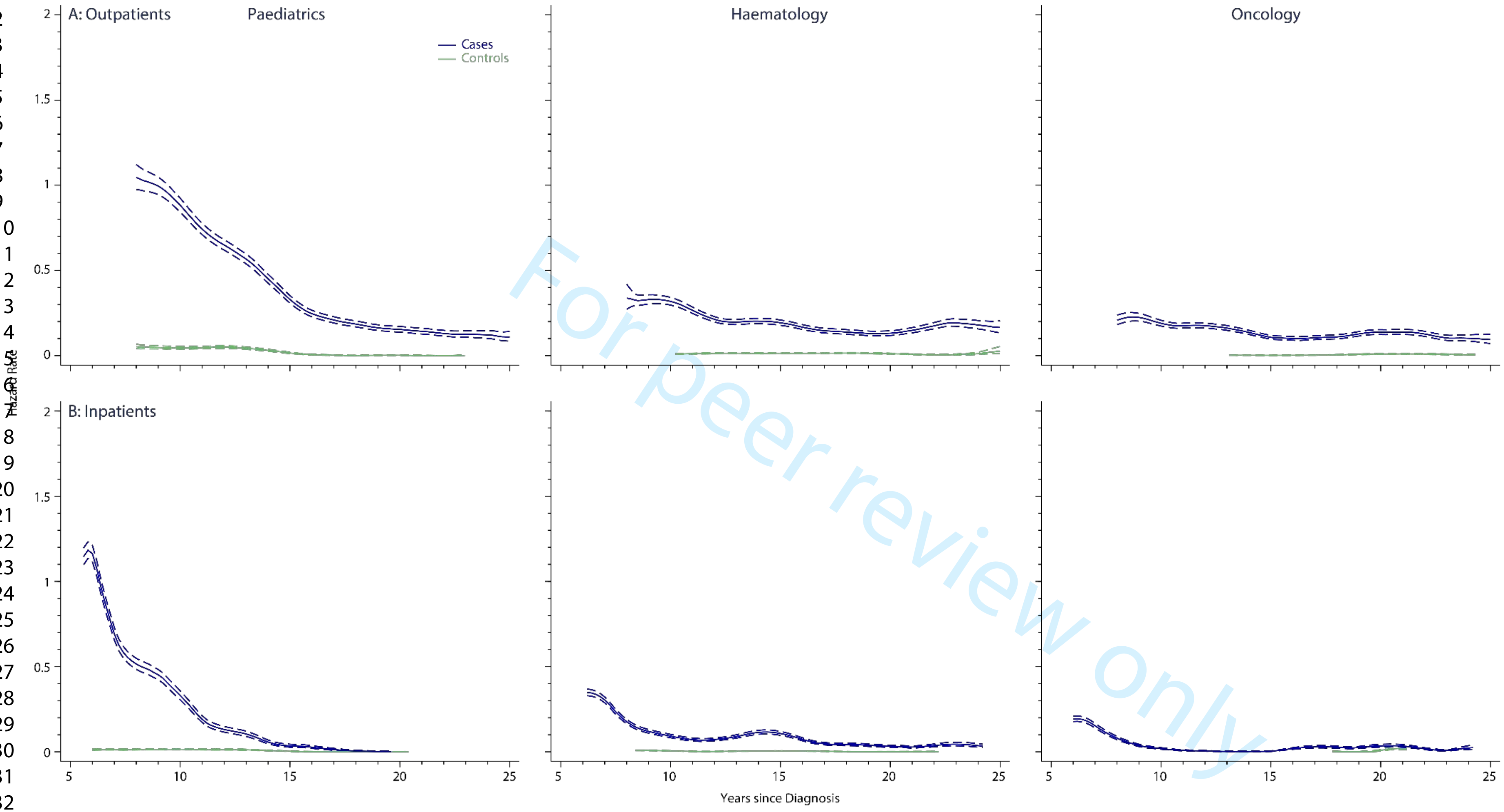

Supplementary Figure 2: Cumulative incidence, rates (per 1000 person-years), attributable risks & incidence rate ratios; top 15 outpatient specialties (excluding paediatrics, haematology & oncology) with ≥2 speciality-specific visits in the 5 to 15 years and 15 to 25 years following diagnosis (cases diagnosed <15 years, 1992-96) and their matched controls

A: Males

£15 Years

	Incidence Rate Cases	Incidence Rate Controls	Attributable Risk (95%CI)	Incidence Rate Ratio (95%CI)
Endocrinology	29.1	0.5	28.6(22.4,34.8)	61.3(20.3,303)
Cardiology/Cardiosurgery	19.0	1.1	17.9(13.0,22.9)	17.1(7.8,44.4)
General medicine	17.5	2.1	15.5(10.7,20.2)	8.5(4.6,16.9)
Ophthalmology	18.6	2.7	15.8(10.8,20.8)	6.8(3.9,12.4)
Respiratory medicine	3.6	0.6	3.0(0.8,5.1)	5.7(1.7,24.1)
Neurology/Neurosurgery	6.4	1.8	4.6(1.7,7.5)	3.6(1.7,8.3)
GP/Nursing/Allied professionals	13.9	4.0	9.8(5.5,14.2)	3.5(2.1,5.9)
Gastroenterology	2.1	0.6	1.4(−0.2,3.1)	3.3(0.8,15.2)
Oral/Dental	21.1	8.6	12.5(6.9,18.2)	2.5(1.7,3.6)
General/Paediatric surgery	8.7	3.6	5.1(1.6,8.7)	2.4(1.3,4.5)
Urology	4.5	2.3	2.3(−0.3,4.9)	2.0(0.9,4.5)
Plastic surgery	4.8	2.4	2.4(−0.3,5.0)	2.0(0.9,4.3)
ENT/Audiology	12.2	6.4	5.8(1.5,10.2)	1.9(1.2,3.1)
Dermatology	11.9	8.2	3.8(−0.6,8.2)	1.5(0.9,2.3)
Trauma & orthopaedics	38.0	38.7	−0.7(−9.6,8.1)	1.0(0.8,1.2)

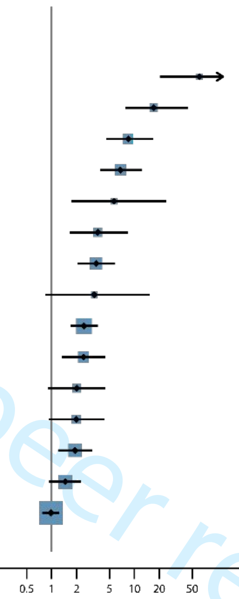

>15–25 Years

	Incidence Rate Cases	Incidence Rate Controls	Attributable Risk (95%CI)	Incidence Rate Ratio (95%CI)
Endocrinology	7.5	0.5	7.0(4.2,9.8)	16.4(5.7,64.5)
General medicine	12.1	1.9	10.2(6.5,13.8)	6.4(3.5,12.1)
Cardiology/Cardiosurgery	12.9	2.2	10.7(6.8,14.7)	5.8(3.3,10.6)
Respiratory medicine	3.5	0.9	2.6(0.8,4.5)	3.9(1.6,10.5)
Neurology/Neurosurgery	4.9	2.2	2.7(0.4,5.0)	2.2(1.1,4.3)
Gastroenterology	4.3	2.3	2.0(−0.1,4.1)	1.9(1.0,3.7)
Urology	5.8	3.2	2.6(0.0,5.1)	1.8(1.0,3.2)
Dermatology	5.3	3.1	2.3(−0.3,4.8)	1.7(0.9,3.2)
ENT/Audiology	7.4	4.5	2.8(−0.2,5.9)	1.6(1.0,2.7)
GP/Nursing/Allied professionals	20.2	12.9	7.3(2.0,12.7)	1.6(1.1,2.1)
General/Paediatric surgery	5.2	3.7	1.5(−1.0,4.1)	1.4(0.8,2.5)
Plastic surgery	3.1	2.7	0.4(−1.6,2.3)	1.1(0.5,2.3)
Ophthalmology	3.7	3.6	0.1(−2.2,2.3)	1.0(0.5,2.0)
Trauma & orthopaedics	15.1	18.0	−2.9(−8.4,2.6)	0.8(0.6,1.2)
Oral/Dental	6.1	7.3	−1.2(−4.3,1.9)	0.8(0.5,1.4)

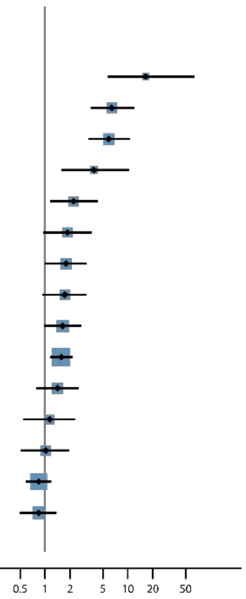

B: Females

£15 Years

	Incidence Rate Cases	Incidence Rate Controls	Attributable Risk (95%CI)	Incidence Rate Ratio (95%CI)
Endocrinology	20.0	0.7	19.2(13.7,24.7)	26.7(9.8,102)
Cardiology/Cardiosurgery	20.3	1.9	18.5(12.9,24.1)	10.8(5.4,23.8)
Respiratory medicine	5.0	0.6	4.5(1.8,7.2)	8.9(2.5,48.5)
General medicine	16.1	2.6	13.5(8.5,18.5)	6.1(3.3,12.1)
Plastic surgery	6.1	2.3	3.8(0.6,7.0)	2.7(1.2,6.1)
Dermatology	17.4	7.4	10.0(4.5,15.6)	2.4(1.5,3.7)
General/Paediatric surgery	12.8	5.7	7.1(2.4,11.8)	2.2(1.3,3.8)
GP/Nursing/Allied professionals	13.4	6.3	7.1(2.3,11.9)	2.1(1.3,3.5)
Neurology/Neurosurgery	5.0	2.6	2.3(−0.6,5.3)	1.9(0.8,4.3)
Ophthalmology	10.8	5.9	4.8(0.4,9.3)	1.8(1.1,3.1)
ENT/Audiology	10.2	6.0	4.2(−0.1,8.5)	1.7(1.0,2.9)
Oral/Dental	22.6	13.9	8.7(2.1,15.4)	1.6(1.1,2.3)
Trauma & orthopaedics	20.0	16.4	3.6(−2.9,10.1)	1.2(0.8,1.7)
Obstetrics & gynaecology	27.9	28.5	−0.6(−8.5,7.3)	1.0(0.7,1.3)
Midwifery	3.1	5.1	−1.9(−4.7,0.9)	0.6(0.3,1.4)

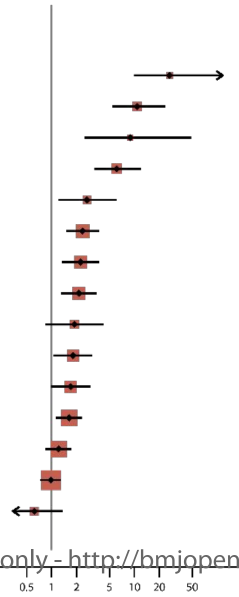

>15–25 Years

	Incidence Rate Cases	Incidence Rate Controls	Attributable Risk (95%CI)	Incidence Rate Ratio (95%CI)
Cardiology/Cardiosurgery	16.1	1.7	14.4(9.7,19.0)	9.3(4.9,18.7)
Endocrinology	5.9	1.3	4.6(1.9,7.2)	4.5(2.0,10.7)
General medicine	12.4	4.0	8.4(4.3,12.4)	3.1(1.9,5.2)
Respiratory medicine	2.5	1.0	1.5(−0.2,3.2)	2.4(0.9,7.0)
Ophthalmology	6.9	3.2	3.7(0.7,6.6)	2.1(1.2,3.9)
Neurology/Neurosurgery	5.4	2.8	2.5(−0.1,5.2)	1.9(1.0,3.7)
Plastic surgery	3.6	2.1	1.5(−0.7,3.6)	1.7(0.8,3.7)
GP/Nursing/Allied professionals	32.6	22.3	10.3(2.9,17.6)	1.5(1.1,1.9)
General/Paediatric surgery	13.5	10.7	2.8(−1.8,7.5)	1.3(0.9,1.9)
Obstetrics & gynaecology	66.6	58.0	8.6(−3.7,21.0)	1.1(0.9,1.4)
ENT/Audiology	6.9	6.2	0.6(−2.6,3.9)	1.1(0.6,1.8)
Oral/Dental	9.8	9.1	0.7(−3.4,4.9)	1.1(0.7,1.7)
Midwifery	25.3	26.6	−1.3(−7.8,5.2)	1.0(0.7,1.2)
Dermatology	7.4	9.1	−1.8(−5.4,1.9)	0.8(0.5,1.3)
Trauma & orthopaedics	9.6	12.1	−2.5(−6.8,1.8)	0.8(0.5,1.2)

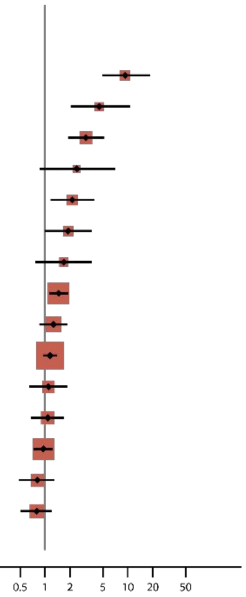

For peer review only

STROBE Statement—Checklist of items that should be included in reports of ***cohort studies***

	Item No	Recommendation	Page No
**Title and abstract**	1	(*a*) Indicate the study's design with a commonly used term in the title or the abstract	2&3
		(*b*) Provide in the abstract an informative and balanced summary of what was done and what was found	2
**Introduction**			
Background/rationale	2	Explain the scientific background and rationale for the investigation being reported	3
Objectives	3	State specific objectives, including any prespecified hypotheses	3
**Methods**			
Study design	4	Present key elements of study design early in the paper	3-4
Setting	5	Describe the setting, locations, and relevant dates, including periods of recruitment, exposure, follow-up, and data collection	3-4
Participants	6	(*a*) Give the eligibility criteria, and the sources and methods of selection of participants. Describe methods of follow-up	3-4
		(*b*) For matched studies, give matching criteria and number of exposed and unexposed	3-4; Fig 1
Variables	7	Clearly define all outcomes, exposures, predictors, potential confounders, and effect modifiers. Give diagnostic criteria, if applicable	3-5
Data sources/ measurement	8*	For each variable of interest, give sources of data and details of methods of assessment (measurement). Describe comparability of assessment methods if there is more than one group	4-5
Bias	9	Describe any efforts to address potential sources of bias	3-4
Study size	10	Explain how the study size was arrived at	3-4
Quantitative variables	11	Explain how quantitative variables were handled in the analyses. If applicable, describe which groupings were chosen and why	3-4
Statistical methods	12	(*a*) Describe all statistical methods, including those used to control for confounding	3-4
		(*b*) Describe any methods used to examine subgroups and interactions	NA
		(*c*) Explain how missing data were addressed	Fig 1
		(*d*) If applicable, explain how loss to follow-up was addressed	Fig 1
		(*e*) Describe any sensitivity analyses	NA
**Results**			
Participants	13*	(a) Report numbers of individuals at each stage of study—eg numbers potentially eligible, examined for eligibility, confirmed eligible, included in the study, completing follow-up, and analysed	Tab 1
		(b) Give reasons for non-participation at each stage	3
		(c) Consider use of a flow diagram	Fig 1
Descriptive data	14*	(a) Give characteristics of study participants (eg demographic, clinical, social) and information on exposures and potential confounders	Tab 1
		(b) Indicate number of participants with missing data for each variable of interest	3-4; Fig 1
		(c) Summarise follow-up time (eg, average and total amount)	Tab 1
Outcome data	15*	Report numbers of outcome events or summary measures over time	5-6; Tab 2 Fig 1

For peer review only - http://bmjopen.bmj.com/site/about/guidelines.xhtml

Main results	16	(*a*) Give unadjusted estimates and, if applicable, confounder-adjusted estimates and their precision (eg, 95% confidence interval). Make clear which confounders were adjusted for and why they were included	5-6
		(*b*) Report category boundaries when continuous variables were categorized	5-6
		(*c*) If relevant, consider translating estimates of relative risk into absolute risk for a meaningful time period	Figs 3-4
Other analyses	17	Report other analyses done—eg analyses of subgroups and interactions, and sensitivity analyses	6
**Discussion**			
Key results	18	Summarise key results with reference to study objectives	6-7
Limitations	19	Discuss limitations of the study, taking into account sources of potential bias or imprecision. Discuss both direction and magnitude of any potential bias	6-7
Interpretation	20	Give a cautious overall interpretation of results considering objectives, limitations, multiplicity of analyses, results from similar studies, and other relevant evidence	2; 6-7
Generalisability	21	Discuss the generalisability (external validity) of the study results	7
**Other information**			
Funding	22	Give the source of funding and the role of the funders for the present study and, if applicable, for the original study on which the present article is based	7

*Give information separately for exposed and unexposed groups.

Note: An Explanation and Elaboration article discusses each checklist item and gives methodological background and published examples of transparent reporting. The STROBE checklist is best used in conjunction with this article (freely available on the Web sites of PLoS Medicine at http://www.plosmedicine.org/, Annals of Internal Medicine at http://www.annals.org/, and Epidemiology at http://www.epidem.com/). Information on the STROBE Initiative is available at http://www.strobe-statement.org.

For peer review only

For peer review only - http://bmjopen.bmj.com/site/about/guidelines.xhtml

BMJ Open

Excess morbidity and mortality among survivors of childhood acute lymphoblastic leukaemia: 25 years of follow-up from the United Kingdom Childhood Cancer Study (UKCCS) population-based matched cohort

Journal:	*BMJ Open*
Manuscript ID	bmjopen-2021-056216.R1
Article Type:	Original research
Date Submitted by the Author:	09-Jan-2022
Complete List of Authors:	Kane, Eleanor; University of York,<br>Kinsey, Sally; Leeds Teaching Hospitals NHS Trust<br>Bonaventure, Audrey; INSERM, Epidemiology of childhood and adolescent cancers team<br>Johnston, Tom; University of York, Department of Health Sciences<br>Simpson, Jill; University of York, Department of Health Sciences<br>Howell, Debra; University of York, Health Sciences<br>Smith, Alexandra; University of York, Health Sciences<br>Roman, Eve; University of York, Health Sciences
**Primary Subject Heading**:	Epidemiology
Secondary Subject Heading:	Public health
Keywords:	Leukaemia < HAEMATOLOGY, Paediatric oncology < ONCOLOGY, Epidemiology < ONCOLOGY, PUBLIC HEALTH

SCHOLARONE™
Manuscripts

I, the Submitting Author has the right to grant and does grant on behalf of all authors of the Work (as defined in the below author licence), an exclusive licence and/or a non-exclusive licence for contributions from authors who are: i) UK Crown employees; ii) where BMJ has agreed a CC-BY licence shall apply, and/or iii) in accordance with the terms applicable for US Federal Government officers or employees acting as part of their official duties; on a worldwide, perpetual, irrevocable, royalty-free basis to BMJ Publishing Group Ltd ("BMJ") its licensees and where the relevant Journal is co-owned by BMJ to the co-owners of the Journal, to publish the Work in this journal and any other BMJ products and to exploit all rights, as set out in our licence.

The Submitting Author accepts and understands that any supply made under these terms is made by BMJ to the Submitting Author unless you are acting as an employee on behalf of your employer or a postgraduate student of an affiliated institution which is paying any applicable article publishing charge ("APC") for Open Access articles. Where the Submitting Author wishes to make the Work available on an Open Access basis (and intends to pay the relevant APC), the terms of reuse of such Open Access shall be governed by a Creative Commons licence – details of these licences and which Creative Commons licence will apply to this Work are set out in our licence referred to above.

Other than as permitted in any relevant BMJ Author's Self Archiving Policies, I confirm this Work has not been accepted for publication elsewhere, is not being considered for publication elsewhere and does not duplicate material already published. I confirm all authors consent to publication of this Work and authorise the granting of this licence.

For peer review only

Excess morbidity and mortality among survivors of childhood acute lymphoblastic leukaemia: 25 years of follow-up from the United Kingdom Childhood Cancer Study (UKCCS) population-based matched cohort

Eleanor Kane[1], Sally Kinsey [1,2], Audrey Bonaventure[3], Tom Johnston[1], Jill Simpson[1], Debra Howell [1] Alexandra Smith[1], Eve Roman [1,4]

[1] Department of Health Sciences, University of York, UK

[2] Department of Paediatric Haematology, Leeds Teaching Hospital's NHS Trust, Leeds, UK

[3] CRESS, Université de Paris, INSERM UMR-1153, Epidemiology of childhood and adolescent cancers team, Villejuif, France

[4] Correspondence to: eve.roman@york. ac.uk, Epidemiology and Cancer Statistics Group, Department of Health Sciences, University of York, York, YO10 5DD, UK

Authors' names and positions

Eleanor Kane (0000-0002-7438-9982), Senior research fellow

Sally Kinsey, (0000-0002-5777-3333), Professor of paediatric haematology

Audrey Bonaventure (0000-0001-6665-8145), Cancer Researcher/Senior Visiting Research Fellow

Tom Johnston (0000-0002-5204-0966), Research fellow

Jill Simpson, (0000-0003-3156-8822), Visiting Research fellow

Debra Howell (0000-0002-7521-7402), Senior research fellow

Alexandra Smith (0000-0002-1111-966X), Professor in cancer epidemiology

Eve Roman (0000-0001-7603-3704), Professor of epidemiology

Word Count (main text) = 2970

For peer review only

For peer review only - http://bmjopen.bmj.com/site/about/guidelines.xhtml

Abstract

Objectives

To examine morbidity and mortality among teenagers and young adults (TYAs) previously diagnosed with acute lymphoblastic leukaemia (ALL) in childhood, and compare to the general TYA population.

Design

National population-based sex- and age-matched case-control study converted into a matched-cohort, with follow-up linkage to administrative healthcare databases.

Setting

The study population comprised all children (0-14 years) registered for primary care with the National Health Service (NHS) in England 1992-1996.

Participants

1082 5-year survivors of ALL diagnosed <15 years (1992-1996) and 2018 unaffected individuals; followed-up to 15/03/2020.

Main outcome measures

Associations with hospital activity, cancer, and mortality were assessed using incidence rate ratios (IRR) and differences.

Results

Mortality in the ALL-cohort was 20 times higher than in the comparison-cohort (Rate Ratio 21.3, 95% Confidence Interval 11.2-45.6), and cancer incidence 10 times higher (IRR 9.9 95% CI 4.1-29.1). Hospital activity was increased for many clinical specialties, the strongest associations being for endocrinology; outpatient IRR 36.7, 95% CI 17.3-93.4 and inpatient 19.7, 95% CI 7.9-63.2 for males, and 11.0, 95% CI 6.2-21.1 and 6.2 95% CI 3.1-13.5 respectively for females. Notable excesses were also evident for cardiology, neurology, ophthalmology, respiratory medicine and general medicine. Males were also more likely to attend gastroenterology, ENT (ear, nose and throat), urology, and dermatology; while females were more likely to be seen in plastic surgery and less likely in midwifery.

Conclusions

Adding to excess risks of death and cancer, survivors of childhood ALL experience excess outpatient and inpatient activity across their TYA years; which is not related to routine follow-up monitoring. Involving most clinical specialties, associations are striking; showing no signs of diminishing over time. Recognising that all survivors are potentially at risk of late treatment-associated effects, our findings underscore the need to take prior ALL diagnosis into account when interpreting seemingly unrelated symptoms later in life.

For peer review only - http://bmjopen.bmj.com/site/about/guidelines.xhtml

For peer review only

ARTICLE SUMMARY

Strengths and limitations of this study

- With minimal selection bias and loss to follow-up, findings from this national study are based on linked hospital data, not self-report
- Ensuring homogeneity of treatment, diagnostic dates are contemporaneous with the national UKALL XI RCT, within which 90.5% of those who survived were enrolled
- The individually sex- and age-matched general population controls enable robust baseline effect measures to be calculated across the 25-year follow-up period
- Analyses are constrained by the fact that hospital episodes statistics are primarily collected for administrative and clinical purposes, and not for research

Funding

This work was supported by Blood Cancer UK (grant number 15037) and Cancer Research UK (grant number 29685). AB is supported by a fellowship from the Fondation ARC pour la Recherche sur le Cancer (PDF20190508759). The funders had no role in considering the study design or in the collection, analysis, interpretation of data, writing of the manuscript, or the decision to submit the article for publication.

Competing interests

None of the authors have any competing interests

Data sharing agreement

No additional data available

For peer review only

For peer review only - http://bmjopen.bmj.com/site/about/guidelines.xhtml

Introduction

Comprising around 40% of all cancers diagnosed in children before 15 years of age, acute lymphoblastic leukaemia (ALL) is the commonest paediatric malignancy in high income countries. Epitomizing one of the major therapeutic success stories of the last 50 years, five-year overall survival for childhood ALL is now around 90% in high-income settings. (1) As a result, the global prevalence of survivors is increasing year-on-year; and will continue to do so as treatments improve, populations age, costs fall, and new clinical collaborations between high- and low-income countries are forged. (2)

In contrast to many other childhood cancers, ALL treatment is prolonged, with current protocols typically administering chemotherapy in phases extending over a 2- to 3-year period. (3) That children who survive ALL tend to have higher mortality and morbidity in later life, including subsequent cancers and a wide range of other therapy-related conditions (Table 1), is established. (4–7) With respect to the latter, however, much of the information on late effects among survivors has come either from analyses of hospitalizations or from questionnaires completed by consenting individuals.(8–17) As is evident from the studies summarized in Table 1 (which includes those that examined all leukaemias combined and those that also included young adults), less attention has been paid to chronic conditions that do not require hospital admission, (7,18–20) and to morbidity patterns that change over time. (7,12,21)

The longitudinal data examined in this report contain information on hospital outpatient attendances, as well as inpatient episodes, cancer registrations, and deaths. Adding significantly to the existing knowledge base, a large cohort of children (<15 years) diagnosed with ALL in England during 1992-6 and their individually sex- and age-matched population controls have been tracked for up to 25 years through their teenage and young adult (TYA) years. Increasing the power to detect effects should they exist, this diagnostic time period (1992-6) coincides with the UKALL XI randomised controlled trial (RCT) (22); the last very intensive UK RCT prior to the era of clinical investigations enabling reduction in therapy for patients with rapid early response to treatment. (23)

Methods

Data are from the United Kingdom Childhood Cancer Study (UKCCS); a national population-based case-control study established in the 1990s to investigate potential causes of childhood cancer that has now been converted to a population-based cohort study to examine survivorship. (24) Full details of the UKCCS's original methods and ethical permissions have been published. (25–27) Briefly, the study population comprised all children (0-14 years) registered for primary care with the National Health Service (NHS) 1992-1996 (England and Wales) and 1991-1995 (Scotland). All children newly diagnosed with cancer (cases) were ascertained via proactive notification systems established in all treatment centres across England, Scotland and Wales; and each child whose parents agreed to be interviewed (87%; all cancers combined) was individually matched on sex, date of birth and region of residence to up to 10 randomly selected controls. The general practitioners (GPs) of the first two controls identified ("first-choice controls") were approached and, with their permission, the parents of the children were contacted and asked to participate in the study. Overall, 72% of first-choice control families participated; but if the GP refused permission to contact

For peer review only - http://bmjopen.bmj.com/site/about/guidelines.xhtml

the parents, or the parents themselves declined, the next control on the list was selected, and so on until two control families agreed. (25–27) For methodological purposes (26,27), details of all non-participating cases and controls were retained in the UKCCS registration database.

With a view to examining childhood cancer sequelae, the UKCCS in England now operates on a legal basis that permits information to be obtained from NHS administrative healthcare records without explicit consent; and all registered cases and first-choice controls, along with replacement controls whose parents participated, are now being prospectively tracked by NHS Digital (https://digital.nhs.uk/) via linkage to nationwide (England) information on deaths, cancer registrations, and Hospital Episode Statistics (HES; inpatient day and overnight admissions, and outpatient appointments and visits). Mortality, cancer incidence, and secondary healthcare utilization of children diagnosed with ALL who survived for 5 years or more is compared here to that of individually sex- and age-matched general-population controls using a matched population-based cohort design.

Data and statistical analysis

The number of ALL cases diagnosed in the original study and their corresponding first-choice controls, as well as subsequent deaths and losses to follow-up occurring in England within 5 years of ALL diagnosis/pseudo-diagnosis are shown in Figure 1. Overall, 1574/1580 (99.6%) cases and 2918/2923 (99.8%) of their first-choice controls were successfully traced and linked to national administrative databases. During the study period (1992 to 1996), 1372/1580 children were diagnosed with ALL in England, and the parents of 92% (1262/1372) of these agreed to participate, each of whom had two sex-and age-matched first-choice controls selected (n=2524).

In the original case-control study, each unaffected control child was assigned a pseudo-diagnosis date that corresponded to their matched case's exact age at diagnosis; and the dates of diagnosis/pseudo-diagnosis now mark the start of follow-up for the matched cohorts. Of the 1104 ALL cases (80.5%) diagnosed in England who survived for 5 years or more, 1004 (90.9%) had B-ALL and 70 (6.3%) T-ALL. The cell lineage of the remaining eight ALL survivors is unknown; they were not entered into a clinical trial and the parents did not participate in the original study (hence no controls were selected). The 22 surviving case children with Down syndrome (no first-choice control had Down syndrome) and their matched controls were excluded from the survivorship analysis because Down syndrome is a well-established risk factor for ALL, and it is also associated with a range of other morbidities.

For all of the 1082 cases and 2018 controls included in this report (Figure 1), linked healthcare data were available on cancer registrations and deaths up to March 2020, inpatient HES from April 1997 to March 2019, and outpatient HES from April 2003 to March 2019. Follow-up ended either at the date of death, 25 years post-diagnosis, or end date for hospital data. For controls, follow-up could also end when their matched case died. Migrations out of England and any subsequent returns were accounted for, and since there is no risk of a hospital visit during an admission, days spent in hospital were also excluded from the follow-up time.

Although the International Classification of Diseases (ICD) is used to code deaths and cancers, it is not routinely used in outpatient HES, and the ICD codes in inpatient HES are not easily assigned to specific events. Each HES episode (inpatient and outpatient) is, however, assigned to an individual consultant whose clinical specialty categorization is recorded; and these categorizations were used in the HES analyses presented here. Firstly, since childhood ALL survivors are routinely monitored via outpatient visits to paediatrics, haematology and/or oncology in the UK, HES attendances to these clinical specialties were separated from those to "other specialties" at the outset (Figure 2).

For peer review only - http://bmjopen.bmj.com/site/about/guidelines.xhtml

All subsequent specialty-specific analyses presented in this report were restricted to the "other specialties" dataset. As in previous reports (28,29), a single inpatient admission and/or two or more specialty-specific face-to-face outpatient attendances were used to indicate potential health issues. Exact methods were used to plot estimates of the hospital visit hazard rates before calculating cumulative incidence (considering death as a competing risk), and risks associated with specific clinical specialties. All specialties were analysed, and findings for the 15 specialties with the highest cumulative incidences among the cases are presented. All analyses were conducted using Stata 16.1.

Patient Public Involvement and Engagement (PPIE)

The original case-control study was established over 30 years ago, and did not benefit from formal links with patient/user groups. Members of the CCLG (Children's Cancer and Leukaemia Group, previously the UK Children's Cancer Study Group) were, however, fully involved throughout. More recently with respect to the present research, the acceptability of using patient data without explicit consent (as is the case in this report) has been positively discussed with patient groups and clinicians; and the study website (www.UKCCS.org) contains information about ongoing research activities and fair processing. It is our intention to establish a PPIE group to input into future research when the COVID pandemic permits.

Results

The characteristics of individuals in the original case-control study and in the 5-year survivor cohorts are shown in Table 2. As expected, the 290 (21%) children with ALL who died within five years of diagnosis were more likely to have had T-cell disease (χ^2=44.0, p<0.001), have been infants or older children (χ^2=100.9, p<0.001), and were less likely to have participated in a clinical trial (χ^2=11.6, p=0.001). Tracking children from 5 years after ALL diagnosis to death or 25 years from diagnosis, this report's primary focus is teenage and young adult (TYA) survivors; as they were aged around 9 years at the start of follow-up (median age 9.4 years, InterQuartile Range 8.0-12.1 years) and almost 30 by the end (median attained age 29.6 years, IQR 27.3-32.1). Between these ages, again as expected, the 5-year ALL survivors continued to experience more deaths (cumulative mortality 10.7%, 95% Confidence Interval 9.0-12.8) than their general population counterparts (cumulative mortality 0.6%, 95% CI 0.3-1.1), yielding a mortality rate ratio of 21.3 (95% CI 11.2-45.6); and were more likely to have a subsequent cancer registration (non-ALL malignancy Incidence Rate Ratio 9.9, 95% CI 4.1-29.1), although the numbers are small (32 *versus* 6).

Childhood ALL survivors are regularly monitored throughout their TYA years either in paediatrics, haematology or oncology, as is evidenced in Figure 2A which shows the outpatient activity of 5-year ALL survivors and their corresponding controls for these three clinical specialties combined (specialty-specific outpatient and inpatient data are shown in Supplementary Figure 1). At around one visit in 20 years, hospital activity among population controls is very low, and varies little over time. By contrast, for ALL survivors, 8 years after diagnosis outpatient activity is around 35 times higher than the background rate, falling 10 years later to around 0.4 visits per case per year (Figure 2A). In all other clinical specialties combined, outpatient activity among childhood ALL survivors are also consistently higher than expected; hazard rates in both cohorts increasing over time at the same steady rate (Figure 2B).

More information on hospital activity is presented for the top 15 specialties (excluding paediatrics, haematology and oncology) for males and females separately in Figure 3 (outpatients) and Figure 4 (inpatients). In all figures, cumulative incidence frequencies are on the left and incidence rates on the right; with the ordering of specialties determined by the magnitude of the incidence rate ratio

For peer review only - http://bmjopen.bmj.com/site/about/guidelines.xhtml

(IRR). Most specialties are associated with high relative and attributable risks (inpatient and outpatient). Furthermore, although the magnitude of the estimates often varies between males and females, the overall pattern is broadly similar. In both sexes, for example, the strongest effects are seen for endocrinology; the IRRs for outpatients and inpatients being 36.7 (95% CI 17.3-93.4) and 19.7 (95% CI 7.9-63.2) respectively for males, and 11.0 (95% CI 6.2-21.1) and 6.2 (95% CI 3.1-13.5) for females. Notable excesses (relative and attributable) are also evident for cardiology, neurology, ophthalmology, respiratory medicine and general medicine, for both genders. Male survivors were also more likely than their peers to attend gastroenterology, ENT (ear, nose and throat), urology, and dermatology; while female survivors were twice as likely as their controls to be referred to plastic surgery. Interestingly, no case-control differences are evident for trauma and orthopaedics, which has the highest background cumulative incidence in males. Likewise, among females, no differences were detected for obstetrics and gynaecology; however, it is notable that activity is lower than expected in midwifery (outpatient IRR 0.9, 95% CI 0.7-1.2; inpatient IRR 0.6, 95% CI 0.4-0.9).

Detailed inspection of the HES data revealed no evidence of specialty-specific changes in either relative or attributable risks over the 20-year time frame (Supplementary Figure 2); possible exceptions being falls in ranking for ophthalmology among males and dermatology among females. Furthermore, no differences between B- and T-cell survivors with respect to any of the outcomes were evident, although the number with T-cell disease was comparatively small (n=70).

Discussion

Including data on more than 1000 survivors of childhood ALL and twice as many age- and sex-matched general population controls, this national record-linkage study found that survivors continued to experience large excesses in mortality and morbidity throughout their teenage and young adult years. With hospital activity being higher than expected in specialties covering most organ and tissue systems, survivors were more than twice as likely to fall under the care of endocrinology, cardiology, respiratory medicine, ophthalmology, neurology and/or gastroenterology specialists. Moreover, aside from a gradual decline in visits to clinical specialties involved in follow-up monitoring (paediatrics, haematology and oncology), there was little indication of activity returning to general population levels up to 25 years after the initial ALL diagnosis. Among females, it is also notable that midwifery outpatient attendances were not increased, with admissions being lower than expected.

Our study, which uses linked rather than self-reported data and population controls, is one of only two (18) to separate secondary care activity associated with cancer follow-up from activity associated with other morbidities; finding that survivors not only experienced higher rates of admission but also higher rates of outpatient visits, neither of which abated over their TYA years. Reported less often are the types of conditions underlying these associations, with four studies assessing risks by organ system (9,13,15,18). Survivors in these studies were diagnosed over a wider period than the study reported here, and had median follow-up times that were shorter; 9 to 17 years from diagnosis, compared to 25 years here. These studies found that survivors were at increased risk of being hospitalised with endocrine or infectious diseases, as well as diseases involving the nervous, circulatory, skin, digestive, genitourinary, respiratory or musculoskeletal systems. With the information source mostly being inpatient records, reports using data from other healthcare settings are sparse (7,18) and not specific to survivors of ALL (19,20). Importantly, while our findings are broadly consistent with these studies, our methods also highlight excesses in

For peer review only - http://bmjopen.bmj.com/site/about/guidelines.xhtml

ophthalmology, ENT (ear, nose and throat), and oral/dental specialties, as well as decreases in midwifery.

Ensuring homogeneity of treatment, the original study's diagnostic dates were contemporaneous with the national UKALL XI randomized controlled trial (RCT), within which 90.5% of UKCCS 5-year survivors were enrolled (22,30). This comparatively high-intensity RCT was the last UK childhood ALL trial prior to the stratification of patient treatment based on early bone marrow response to induction treatment (measured by percentage of leukaemia cells in the marrow) (23,31). Information on UKALL XI's scheduling of drugs is provided in the report by Hann and colleagues (22), several of which have been implicated, either singly or combined, in therapy-related effects (Table 1). It is, however, important to note that several of the UKALL XI drugs are no longer used (e.g. etoposide, 6-thioguanine) or are used more sparingly (e.g. daunorubicin, cyclophosphamide, corticosteroids), and that cranial irradiation is no longer routinely administered, being reserved for infrequent particular indications (32). Hence, the nature of the UKCCS cohort means that it is well placed to provide baseline data against which to evaluate the potential impact of treatment de-escalation and other protocol modifications that occurred in later years. Other major strengths include its national coverage, completeness of case ascertainment, and large in-built sex- and age-matched general population comparator that has a similar regional and deprivation distribution (IMD - Index of Multiple Deprivation) to the case arm (25–27); the latter obviating the need to rely on published national rates, as has mostly been the case in the UK thus far (10,15,33–35).

With regard to weaknesses, the lack of data from other parts of the NHS, including primary care and adolescent psychosocial services, is an obvious deficiency that currently affects all UK record-linkage studies of the type described here. The relative paucity of information on psychological morbidities is particularly relevant to childhood cancer survivors, who are known to be at increased risk of a number of such disorders (4,36,37). Missing information within the HES datasets is also an issue; diagnostic data, for example, is largely absent from outpatient HES. Furthermore, although steps to mitigate surveillance bias were taken by requiring at least two face-to-face outpatient visits to a given specialty, we cannot rule out the possibility that childhood cancer survivors were more likely to be referred to secondary care than their peers. With virtually complete linkage to existing national databases established, our future research will, however, continue to monitor the health of cancer survivors across their life-span. As the cohort matures and the number of events increase, this will include more detailed examination of second malignancies, as well as factors potentially associated with ALL survival and/or outcome, such as socioeconomic status and treatment information (e.g. risk strata, and relapse and/or transplant therapy).

Examining data in the broader context of symptomology based on on-going medical referrals, the present report focuses on the health of individuals who survived five years of more following a diagnosis of ALL as a child in the early 1990s. Adding to a large excess risk for death and cancer, we found that survivors experienced excess outpatient and inpatient activity across their teenage and young adult years, which was not related to routine follow-up monitoring. Involving most clinical specialties, the observed associations are striking; showing no signs of diminishing over the first 25 years of follow-up, the main exception is the comparatively low midwifery contact seen among female ALL survivors. All survivors of ALL are at increased risk of late effects from treatment, and our findings underscore the need to take prior ALL diagnosis into account when interpreting seemingly unrelated symptoms in later life, which currently might not be recognized by specialties outside haemato-oncology. As importantly, there is also a need for provision of information to survivors themselves to encourage them to declare previous treatment for ALL as a child when they access healthcare.

For peer review only - http://bmjopen.bmj.com/site/about/guidelines.xhtml

Ethical approval: The United Kingdom Childhood Cancer Study (UKCCS) has ethics approval from the Yorkshire & The Humber-Leeds West Research Ethics Committee (reference 18/YH/0135) and exemption from Section 251 of the Health & Social Care Act (reference 18/CAG/0066).

Contributors: ER, SK, and JS contributed to the design of the original study; and ER and EK instigated the present investigation and prepared the report. EK conducted the analyses, and all authors (ER, EK, SK, AB, TJ, JS, DH, AS) were involved in the development, reviewing, and approving the final manuscript. ER is the principal investigator and guarantor. The corresponding author (ER) attests that all listed authors meet the authorship criteria and that no others meeting the criteria have been omitted.

For peer review only

For peer review only - http://bmjopen.bmj.com/site/about/guidelines.xhtml

References

1. Bonaventure A, Harewood R, Stiller CA, Gatta G, Clavel J, Stefan DC, et al. Worldwide comparison of survival from childhood leukaemia for 1995–2009, by subtype, age, and sex (CONCORD-2): a population-based study of individual data for 89 828 children from 198 registries in 53 countries. The Lancet Haematology. 2017 May 1;4(5):e202–17.

2. The European Society for Paediatric Oncology. The SIOPE Strategic Plan. A European Cancer Plan for Children and Adolescents. [Internet]. SIOP Europe; 2015 Sep. Available from: https://siope.eu/european-strategic-plan/

3. Teachey DT, Hunger SP, Loh ML. Optimizing therapy in the modern age: differences in length of maintenance therapy in acute lymphoblastic leukemia. Blood. 2021 Jan 14;137(2):168–77.

4. Chow EJ, Ness KK, Armstrong GT, Bhakta N, Yeh JM, Bhatia S, et al. Current and coming challenges in the management of the survivorship population. Semin Oncol. 2020;47(1):23–39.

5. Landier W, Skinner R, Wallace WH, Hjorth L, Mulder RL, Wong FL, et al. Surveillance for Late Effects in Childhood Cancer Survivors. J Clin Oncol. 2018 Jul 20;36(21):2216–22.

6. Norsker FN, Pedersen C, Armstrong GT, Robison LL, McBride ML, Hawkins M, et al. Late Effects in Childhood Cancer Survivors: Early Studies, Survivor Cohorts, and Significant Contributions to the Field of Late Effects. Pediatric Clinics of North America. 2020 Dec 1;67(6):1033–49.

7. Jensen KS, Klug Albertsen B, Schrøder H, Zalounina Falborg A, Schmiegelow K, Rosthøj S, et al. Health care utilisation following childhood acute lymphoblastic leukaemia: a population-based matched cohort study. BMJ Open. 2021 Nov 22;11(11):e049847.

8. Dixon SB, Chen Y, Yasui Y, Pui C-H, Hunger SP, Silverman LB, et al. Reduced Morbidity and Mortality in Survivors of Childhood Acute Lymphoblastic Leukemia: A Report From the Childhood Cancer Survivor Study. JCO. 2020 Jul 24;JCO.20.00493.

9. Sørensen GV, Winther JF, de Fine Licht S, Andersen KK, Holmqvist AS, Madanat-Harjuoja L, et al. Long-term risk of hospitalization among five-year survivors of childhood leukemia in the Nordic countries. J Natl Cancer Inst [Internet]. 2019 Feb 11; Available from: https://www.ncbi.nlm.nih.gov/pmc/articles/PMC6748786/pdf/djz016.pdf

10. Reulen RC, Guha J, Bright CJ, Henson KE, Feltbower RG, Hall M, et al. Risk of cerebrovascular disease among 13 457 five-year survivors of childhood cancer: A population-based cohort study. International Journal of Cancer. 2021;148(3):572–83.

11. Streefkerk N, Tissing WJE, Korevaar JC, van Dulmen-den Broeder E, Bresters D, van der Heiden-van der Loo M, et al. A detailed insight in the high risks of hospitalizations in long-term childhood cancer survivors-A Dutch LATER linkage study. PLoS ONE. 2020;15(5):e0232708.

12. Mulrooney DA, Hyun G, Ness KK, Bhakta N, Pui C-H, Ehrhardt MJ, et al. The changing burden of long-term health outcomes in survivors of childhood acute lymphoblastic leukaemia: a retrospective analysis of the St Jude Lifetime Cohort Study. Lancet Haematol. 2019 Jun;6(6):e306–16.

13. Mueller BA, Doody DR, Weiss NS, Chow EJ. Hospitalization and mortality among pediatric cancer survivors: a population-based study. Cancer Causes Control. 2018 Nov;29(11):1047–57.

For peer review only - http://bmjopen.bmj.com/site/about/guidelines.xhtml

14. Ou JY, Smits-Seemann RR, Kaul S, Fluchel MN, Sweeney C, Kirchhoff AC. Risk of hospitalization among survivors of childhood and adolescent acute lymphoblastic leukemia compared to siblings and a general population sample. Cancer Epidemiol. 2017;49:216–24.

15. Brewster DH, Clark D, Hopkins L, Bauer J, Wild SH, Edgar AB, et al. Subsequent hospitalisation experience of 5-year survivors of childhood, adolescent, and young adult cancer in Scotland: a population based, retrospective cohort study. Br J Cancer. 2014 Mar 4;110(5):1342–50.

16. Oudin C, Berbis J, Bertrand Y, Vercasson C, Thomas F, Chastagner P, et al. Prevalence and characteristics of metabolic syndrome in adults from the French childhood leukemia survivors' cohort: a comparison with controls from the French population. Haematologica. 2018 Apr;103(4):645–54.

17. Hau EM, Caccia JN, Kasteler R, Spycher B, Suter T, Ammann RA, et al. Cardiovascular disease after childhood acute lymphoblastic leukaemia: a cohort study. Swiss Medical Weekly [Internet]. 2019 Mar 10 [cited 2020 Oct 17];149(0910). Available from: https://smw.ch/article/doi/smw.2019.20012

18. Holmqvist AS, Moëll C, Hjorth L, Lindgren A, Garwicz S, Wiebe T, et al. Increased health care utilization by survivors of childhood lymphoblastic leukemia is confined to those treated with cranial or total body irradiation: a case cohort study. BMC Cancer. 2014 Jun 10;14:419.

19. McBride ML, Lorenzi MF, Page J, Broemeling A-M, Spinelli JJ, Goddard K, et al. Patterns of physician follow-up among young cancer survivors: report of the Childhood, Adolescent, and Young Adult Cancer Survivors (CAYACS) research program. Can Fam Physician. 2011 Dec;57(12):e482-490.

20. Chang WH, Katsoulis M, Tan YY, Mueller SH, Green K, Lai AG. Late effects of cancer in children, teenagers and young adults: Population-based study on the burden of 183 conditions, in-patient and critical care admissions and years of life lost. The Lancet Regional Health - Europe. 2021 Nov 14;100248.

21. Gibson TM, Mostoufi-Moab S, Stratton KL, Leisenring WM, Barnea D, Chow EJ, et al. Temporal patterns in the risk of chronic health conditions in survivors of childhood cancer diagnosed 1970-99: a report from the Childhood Cancer Survivor Study cohort. Lancet Oncol. 2018;19(12):1590–601.

22. Hann I, Vora A, Richards S, Hill F, Gibson B, Lilleyman J, et al. Benefit of intensified treatment for all children with acute lymphoblastic leukaemia: results from MRC UKALL XI and MRC ALL97 randomised trials. UK Medical Research Council's Working Party on Childhood Leukaemia. Leukemia. 2000 Mar;14(3):356–63.

23. Mitchell C, Payne J, Wade R, Vora A, Kinsey S, Richards S, et al. The impact of risk stratification by early bone-marrow response in childhood lymphoblastic leukaemia: results from the United Kingdom Medical Research Council trial ALL97 and ALL97/99. British Journal of Haematology. 2009;146(4):424–36.

24. Berrington de González A, Morton LM. Converting epidemiologic studies of cancer etiology to survivorship studies: approaches and challenges. Cancer Epidemiol Biomarkers Prev. 2012 Jun;21(6):875–80.

25. Johnston WT, Lightfoot TJ, Simpson J, Roman E. Childhood cancer survival: a report from the United Kingdom Childhood Cancer Study. Cancer Epidemiol. 2010 Dec;34(6):659–66.

For peer review only - http://bmjopen.bmj.com/site/about/guidelines.xhtml

26. UK Childhood Cancer Study Investigators. The United Kingdom Childhood Cancer Study: objectives, materials and methods. Br J Cancer. 2000 Feb 11;82(5):1073–102.

27. Smith A, Roman E, Simpson J, Ansell P, Fear NT, Eden T. Childhood leukaemia and socioeconomic status: fact or artefact? A report from the United Kingdom childhood cancer study (UKCCS). International Journal of Epidemiology. 2006 Dec 1;35(6):1504–13.

28. Kane E, Painter D, Smith A, Crouch S, Oliver S, Patmore R, et al. The impact of rheumatological disorders on lymphomas and myeloma: a report on risk and survival from the UK's population-based Haematological Malignancy Research Network. Cancer Epidemiology. 2019 Apr 1;59:236–43.

29. Lamb MJ, Smith A, Painter D, Kane E, Bagguley T, Newton R, et al. Health impact of monoclonal gammopathy of undetermined significance (MGUS) and monoclonal B-cell lymphocytosis (MBL): findings from a UK population-based cohort. BMJ Open. 2021 Feb 22;11(2):e041296.

30. Chessells JM, Harrison CJ, Watson SL, Vora AJ, Richards SM. Treatment of infants with lymphoblastic leukaemia: results of the UK Infant Protocols 1987–1999. British Journal of Haematology. 2002;117(2):306–14.

31. Inaba H, Pui C-H. Advances in the Diagnosis and Treatment of Pediatric Acute Lymphoblastic Leukemia. Journal of Clinical Medicine. 2021 Jan;10(9):1926.

32. Brown P, Inaba H, Annesley C, Beck J, Colace S, Dallas M, et al. Pediatric Acute Lymphoblastic Leukemia, Version 2.2020, NCCN Clinical Practice Guidelines in Oncology. J Natl Compr Canc Netw. 2020 Jan;18(1):81–112.

33. Smith L, Glaser AW, Peckham D, Greenwood DC, Feltbower RG. Respiratory morbidity in young people surviving cancer: Population-based study of hospital admissions, treatment-related risk factors and subsequent mortality. Int J Cancer. 2019 01;145(1):20–8.

34. Reulen RC, Frobisher C, Winter DL, Kelly J, Lancashire ER, Stiller CA, et al. Long-term risks of subsequent primary neoplasms among survivors of childhood cancer. JAMA. 2011 Jun 8;305(22):2311–9.

35. Fidler MM, Reulen RC, Winter DL, Kelly J, Jenkinson HC, Skinner R, et al. Long term cause specific mortality among 34 489 five year survivors of childhood cancer in Great Britain: population based cohort study. BMJ. 2016 Sep 1;354:i4351.

36. Andrés-Jensen L, Skipper MT, Mielke Christensen K, Hedegaard Johnsen P, Aagaard Myhr K, Kaj Fridh M, et al. National, clinical cohort study of late effects among survivors of acute lymphoblastic leukaemia: the ALL-STAR study protocol. BMJ Open [Internet]. 2021 Feb 9 [cited 2021 May 4];11(2). Available from: https://www.ncbi.nlm.nih.gov/pmc/articles/PMC7875271/

37. van Erp LME, Maurice-Stam H, Kremer LCM, Tissing WJE, van der Pal HJH, de Vries ACH, et al. A vulnerable age group: the impact of cancer on the psychosocial well-being of young adult childhood cancer survivors. Support Care Cancer. 2021 Aug;29(8):4751–61.

For peer review only

Legends

Table 1: Studies reporting morbidity among 5-year survivors of leukaemia diagnosed in children, teenagers, and young adults; both acute lymphoblastic leukaemia (ALL) alone, and all leukaemias combined

Table 2: Characteristics of acute lymphoblastic leukaemia (ALL) cases diagnosed <15 years in England 1992-96 and their age- and sex-matched first-choice controls[1]: total registered in the original case-control study, and 5-year survivors included in the present study

Figure 1: Flowchart showing subjects targeted in the original United Kingdom Childhood Cancer case-control study and those included in the present cohort

Figure 2: Outpatient activity hazard rates per year and 95% confidence intervals (dotted lines) for childhood (< 15 years) ALL 5-year survivors and their matched controls: A) paediatrics, haematology and oncology, B) all other clinical specialties

Figure 3: Cumulative incidence (%), incidence rates (per 1000 person-years), attributable risks, and incidence rate ratios for the top 15 outpatient specialties (excluding paediatrics, haematology and oncology) with two or more face-to-face speciality-specific visits in the 5 to 25 years following diagnosis (cases diagnosed <15 years, 1992-96) and their matched population controls

Figure 4: Cumulative incidence (%), incidence rates (per 1000 person-years), attributable risks, and incidence rate ratios for the top 15 inpatient specialties (excluding paediatrics, haematology and oncology) with one or more specialty-specific admissions in the 5 to 25 years following diagnosis (cases diagnosed aged<15 years, 1992-96) and their matched population controls.

Supplementary Figure 1: Hazard rates per year of activity at paediatrics, haematology and oncology among ALL cases who survived 5 years or more and their matched population controls: UKCCS, ALL diagnosed aged<15 years, 1992-96.

Supplementary Figure 2: Cumulative incidence (%), incidence rates (per 1000 person-years), attributable risks and incidence rate ratios for the top 15 outpatient specialties (excluding paediatrics, haematology and oncology) with two of more face-to-face speciality-specific visits in the 5 to 15 years and 15 to 25 years following diagnosis (cases diagnosed <15 years, 1992-96) and their matched population controls.

For peer review only - http://bmjopen.bmj.com/site/about/guidelines.xhtml

Table 1: Studies reporting morbidity among 5-year survivors of leukaemia diagnosed in children, teenagers, and young adults; both acute lymphoblastic leukaemia (ALL) alone, and all leukaemias combined

Study	5-year survivors		Comparator Source	Follow-up[1] Source, Timing post-diagnosis	Outcome Measures	Main Findings
	Setting, Timeframe	Numbers, Completeness				
**Acute lymphoblastic leukaemia**						
Childhood Cancer Survivor Study, CCSS (8)	USA & Canada: 31 centres<br><br>1970-1999	6148 <21yrs<br><br>~72% consented at baseline	5051 controls<br><br>Closest aged siblings in random sample of all childhood cancer survivors	Biannual questionnaires 2000-2017<br><br>5-46yrs post-diagnosis (median 22yrs)	Conditions considered severe by the respondents, distributed by treatment era and treatment group	11% of survivors diagnosed in the 1990s receiving standard treatment reported at least one severe chronic condition in the 20yr after diagnosis; Rate Ratio 1.9 (95%CI 1.5-2.3) compared to controls. RRs>2 for stroke, major joint replacement, and diabetes
Adult Life after Childhood Cancer in Scandinavia, ALiCCS[2] (9)	Denmark, Sweden, Iceland & Finland: National cancer registries<br><br>1970-2008[2]	3391 <20yrs<br><br>100% traced	129 828 controls matched on sex, age, and country<br><br>National population registers	National hospital registers 1975-2012<br><br>5-42yrs post-diagnosis (median 16yrs)	First inpatient admission for 120 conditions, total and by organ system	Survivors were nearly twice (Rate Ratio=1.95, 95%CI 1.83-2.07) as likely to be hospitalised for ≥1 of 120 conditions than controls. RRs>2 for diseases of the endocrine, nervous, and circulatory systems; infections, blood/blood organs, second cancers, and benign neoplasms
St Jude Lifetime Cohort, SJLIFE (12)	USA: St Jude Children's Research Hospital<br><br>1963-2003	980 <18yrs alive 10 years after diagnosis<br><br>~67% consented	272 controls<br><br>Friends, non-first-degree relatives of hospital patients, hospital employees and volunteers	In-person interview 2015<br><br>12-52yrs post-diagnosis (median 25yrs)	Conditions considered by respondents to be moderate/severe, overall, and by organ system	By age 30, survivors reported 3.2 (95%CI 2.9-3.4) chronic conditions compared with 1.2 (95%CI 1.0-1.4) among controls. Conditions varied by treatment era, with endocrine and musculoskeletal conditions contributing more to the cumulative burden in the 1990s and 2000s than in earlier decades
Nordic Society of Paediatric Haematology and Oncology ALL registry, NOPHO (7)	Denmark: Acute lymphoblastic leukaemia national registry<br><br>1994-2015	675 aged 1-<18yrs alive 2.5 years after diagnosis<br><br>~96.4% traced	6750 controls matched on age, sex and region<br><br>National population register	National primary care register 1997-2018 and national hospital registers 1997-2017<br><br>2.5-24yrs post diagnosis (median 13.5yrs)	Rates of primary care and hospital (in/outpatient) contacts, by year since diagnosis (2.5-17.5yrs post-diagnosis)	At 2-3yrs post diagnosis, survivors had 14.2 hospital contacts (95%CI 13.4-15.1), 30 times more than controls (Incidence Rate Ratio=31.5, 95%CI 28.3-35.1); contacts decreased over time but remained above baseline through to end of follow-up (IRRs≥2). For primary care, survivors started with 4.75 (95%CI 4.41-5.11) daytime contacts, nearly twice that among controls (IRR=1.85, 95%CI 1.71-2.00); contacts remained at 3-5 times/yr but IRRs declined

For peer review only - http://bmjopen.bmj.com/site/about/guidelines.xhtml

Study	5-year survivors		Comparator Source	Follow-up[1] Source, Timing post-diagnosis	Outcome Measures	Main Findings
	Setting, Timeframe	Numbers, Completeness				
						and were not statistically significant >12yrs post-diagnosis.
Swiss Childhood Cancer Survivor Study, SCCSS (17)	Switzerland: Swiss Childhood Cancer Registry<br><br>1976-2005	511 <16yrs, alive in 2007 and aged≥16yrs at questionnaire<br><br>72% participated	702 siblings	Questionnaire 2007-2012<br><br>5- 36yrs post-diagnosis	Self-reported cardiovascular diseases	14% of survivors reported having at least one cardiovascular disease, nearly twice that of their siblings (Odds Ratio=1.9, 95%CI 1.3-2.8)
Childhood, adolescent and young adult cancer survivors, CAYACS (19)	Canada: British Columbia cancer registry[4]<br><br>1970-1992	242 <20yrs alive at 31 December 2000<br><br>~64% traced	11 570 controls frequency-matched on age and sex<br><br>Health insurance plan	Health insurance claims 1998-2000<br><br>8-20yrs post-diagnosis	Physician visits over three-year period excluding oncology, overall and by specialty	Survivors were nearly twice as likely as controls to visit a doctor at least once (Rate Ratio=1.95, 95%CI 1.8-2.1). RRs≥2 were found for ≥1 visit to neurology, ophthalmology, otolaryngology, internal medicine, surgery, urology, psychiatry, and dermatology
Childhood Cancer Register of Southern Sweden, BORISS (18)	Sweden: Regional cancer registry<br><br>1970-1999	213 <18yrs<br><br>~100% traced	10 650 controls matched on sex, year of birth and county<br><br>National population register	National hospital registers inpatient: 1975-2009, outpatient: 2001-2009<br><br>5-33yrs post-diagnosis (median 16yrs)	Hospital contacts (in/outpatient), overall and for 20 disease categories	Survivors were over twice as likely to have ≥1 hospital contact than controls (Odds Ratio=2.5, 95%CI 1.5-4.3), and were more than twice as likely to consult for disorders of the blood, endocrine system, nervous system, skin, infections or benign neoplasms
University of Utah-Intermountain Healthcare (14)	USA: Primary Children's Hospital, Salt Lake City<br><br>1998-2008	176 <22yrs<br><br>~79% traced	503 controls matched on sex and year of birth<br><br>State population register	State hospital registers 2003-2013<br><br>5-10yrs post-diagnosis (median 9yrs)	Rate of inpatient admissions over a maximum of five years	In the 5 to 10 years after diagnosis, the hospitalisation rate of survivors was 3.76 per 100 person-years (95%CI 2.22-6.36), twice the rate among controls (Relative Hospitalisation Rate=2.01, 95%CI 1.00-4.03)
**<u>All leukaemias combined</u>**						
British Childhood Cancer Survivor Study, BCCSS (10)	UK: National Registry of Childhood Tumours<br><br>1940-1991	3544 <15yrs, alive in April 1997<br><br>~87% being followed	Hospitalisation rates for cerebrovascular disease<br><br>National population and hospital registers	National hospital registers 1997-2012<br><br>5-72yrs post-diagnosis	Cerebrovascular disease recorded in hospital discharge summaries	Survivors were almost five times more likely to have been hospitalised for cerebrovascular disease than expected (Age Standardised Hospitalisation Ratio=4.7, 95%CI 3.6-6.1)

For peer review only - http://bmjopen.bmj.com/site/about/guidelines.xhtml

Study	5-year survivors		Comparator Source	Follow-up[1] Source, Timing post-diagnosis	Outcome Measures	Main Findings
	Setting, Timeframe	Numbers, Completeness				
Dutch Childhood Oncology Group-LATER Study (11)	Netherlands: Paediatric oncology or haematology centres<br><br>1963-2001	1900 <18yrs, alive in January 1995<br><br>~92% traced	38 000 controls matched on sex and year of birth<br><br>National population register	National hospital registers 1995-2015<br><br>5-52yrs post-diagnosis	Rate of inpatient admissions over a maximum of ten years	The hospitalisation rate of survivors was almost twice that of controls (Relative Hospitalisation Rate=1.87, 95%CI 1.65-2.12)
Leucémies de l'Enfant et l'Adolescent Cohort, LEA (16)	France: 16 cancer centres<br><br>1980-2012	1025 <18yrs, alive in 2007 and aged≥18yrs at last LEA medical examination<br><br>72% participated	3203 controls matched on sex and age<br><br>Volunteers for free medical examinations	Medical examinations at 2yr intervals until 10 years post-diagnosis (or age 20), thereafter every 4yrs<br><br>2-34yrs post-diagnosis (mean 16yrs)	Metabolic syndrome found at medical examination	Prevalence of metabolic syndrome among survivors was 10.3%, more than twice that among controls (Odds Ratio=2.49, 95%CI 1.91-2.35)
Scottish Cancer Registry (15)	UK: Scottish cancer registry<br><br>1981-2003	884 <25yrs<br><br>~100% traced	Hospitalisation rates<br><br>National population and hospital registers	National hospital registers 1986-2009<br><br>5-28yrs post-diagnosis (mean 16yrs)	First inpatient admission for particular diseases, overall, and for selected disease categories	Survivors were almost four times more likely to have been hospitalised than expected (Standardised Hospitalisation Ratio=3.9, 95%CI 3.6-4.3). SHR≥2 for diseases of the endocrine, nervous, circulatory, respiratory and digestive systems; infections and neoplasms
Washington State Cancer Registry (13)	USA: Washington State cancer registry<br><br>1982-2008	815 <20yrs<br><br>Not evaluable	8150 controls matched on sex and year of birth and alive at case's diagnosis<br><br>State birth records	State hospital registers 1987-2013<br><br>5-27yrs post-diagnosis (median 9yrs)	Rates of inpatient admissions, and hospitalisation or death for selected disease categories	The survivors' hospitalisation rate was almost three times that of controls (Hazard Ratio=2.8, 95%CI 2.2-3.5). HRs≥2 were observed for hospitalisation or death from endocrine, nervous, circulatory, respiratory, digestive, genitourinary, skin and musculoskeletal systems; blood diseases infections and cancers
Clinical Practice Research Datalink (20)	UK: Clinical Practice Research Datalink<br><br>1998-2020	403 ≤25 years<br><br>Not evaluable	13517 controls matched to all cancers on sex, year of birth, socioeconomic status and general practice<br><br>Primary care registers	Primary care electronic health records linked to national hospital registers from age 18yrs to 2020<br><br>5-22yrs post-diagnosis	Mean cumulative counts of primary care visits and hospital admissions for 183 conditions between ages of 18 and 35 or 45, overall and by organ system	Survivors' mean count of primary care visits was higher than among controls between ages of 18-35yrs (23.5, 95%CI 19.8-29.3 compared to 4.0, 95%CI 3.9-4.1), and 18-45yrs (29.8, 95%CI 24.7-36.0 compared to 7.2, 95%CI 7.1-7.4); as were their mean counts of admissions (18-35yrs: 6.0, 95%CI 5.6-6.3 vs 1.1, 95%CI 1.0-1.1; 18-45yrs: 7.9, 95%CI 7.0-8.0 vs 1.8, 95%CI 1.8-1.9). Survivors' cumulative burdens

For peer review only - http://bmjopen.bmj.com/site/about/guidelines.xhtml

Study	5-year survivors		Comparator Source	Follow-up[1] Source, Timing post-diagnosis	Outcome Measures	Main Findings
	Setting, Timeframe	Numbers, Completeness				
						of cardiovascular conditions and endocrinopathies were higher than controls'.

[1]Minimum to maximum possible follow-up relative to diagnosis date.[2]Finland 1969-2012; Denmark 1977-2010; Sweden 1987-2009 with some regions starting in 1964-1987; Iceland 1999-2008. [3]Completeness could not be evaluated in the Washington State Cancer Registry Study as study subjects were not linked to a population register. [4]CAYACS reported outcomes for all childhood cancer survivors, of which 20% had AL

For peer review only

Table 2: Characteristics of acute lymphoblastic leukaemia (ALL) cases diagnosed <15 years in England 1992-96 and their age- and sex-matched first-choice controls[1]: total registered in the original case-control study, and 5-year survivors included in the present study

	**Original study**		**5-year survivors**	
	**Cases** N (%)	**Controls[1]** N (%)	**Cases** N (%)	**Controls[1]** N (%)
**Total**	1372 (100)	2524 (100)	1082 (100)	2018 (100)
**Sex**				
Male	763 (55.6)	1410 (55.9)	589 (54.4)	1099 (54.5)
Female	609 (44.4)	1114 (44.1)	493 (45.6)	919 (45.5)
**Lineage[1]**	-	-		
B-ALL	1234 (89.9)	-	1004 (92.8)	-
T-ALL	120 (8.7)	-	70 (6.5)	-
**Age (years) at:**				
Diagnosis				
<1	52 (3.8)	86 (3.4)	22 (2.0)	41(2.0)
1-4	839 (61.2)	1578 (62.5)	703 (65.0)	1331 (66.0)
5-9	267 (19.5)	484 (19.2)	220 (20.3)	402 (19.9)
10-14	214 (15.6)	376 (14.9)	137 (12.7)	244 (12.1)
Median (IQR)	4.5 (3.0-7.6)	4.4 (3.0-7.5)	4.4 (3.0-7.1)	4.3 (3.0-7.0)
Beginning of follow-up				
Median (IQR)	-	-	9.4 (8.0-12.1)	9.3 (8.0-12.0)
End of follow-up[2]				
Median (IQR)	-	-	29.6 (27.3-32.1)	29.5 (27.0-31.9)
**IMD[3] quintile at diagnosis**				
Least deprived (1-3)	834 (60.8)	1533 (60.7)	675 (62.4)	1237 (61.3)
Most deprived (4-5)	520 (37.9)	975 (38.6)	400 (37.0)	766 (38.0)
**Trial entry**				
Yes	1215 (88.6)	-	979 (90.5)	-
No	157 (11.4)	-	103 (9.5)	-
**Person-years in follow-up[2]**	-	-	19425.6	35966.0
**Deaths in follow-up**	-	-	115	10
Cumulative mortality, % (95%CI)			10.7 (9.0-12.8)	0.6 (0.3-1.1)
Mortality Rate Ratio (95%CI)			21.3 (11.2-45.6)	
**Cancer[4] registrations in follow-up**	-	-		
Total			60	23
Cumulative incidence, % (95%CI)			5.8 (4.5-7.4)	1.4 (0.9-2.0)
Incidence Rate Ratio (95%CI)			4.9 (3.0-8.9)	
Malignancies only			32	6
Cumulative incidence, % (95%CI)			3.1 (2.2-4.3)	0.4 (0.1-0.8)
Incidence Rate Ratio (95%CI)			9.9 (4.1-29.1)	

[1]Lineage was not confirmed for 18 cases, 8 of whom were alive 5 years after diagnosis.
[2]Follow-up time among controls was censored at the matched survivor's death if the survivor died during follow-up. IQR: interquartile range.
[3]IMD: Index of Multiple Deprivation income domain, IMD was missing for 18 cases and 16 controls.
[4]Total cancer, including tumours that were registered as benign, in situ and of uncertain behaviour: ICD-10 codes C00-C90, C92-C97 and D00-D48. Malignancies only: C00-C43, C45-C90, C92-C97 (NB registrations for lymphoid leukaemia (C91) were excluded). Cumulative incidence of cancer was calculated considering death as a competing risk.

For peer review only - http://bmjopen.bmj.com/site/about/guidelines.xhtml

For peer review only

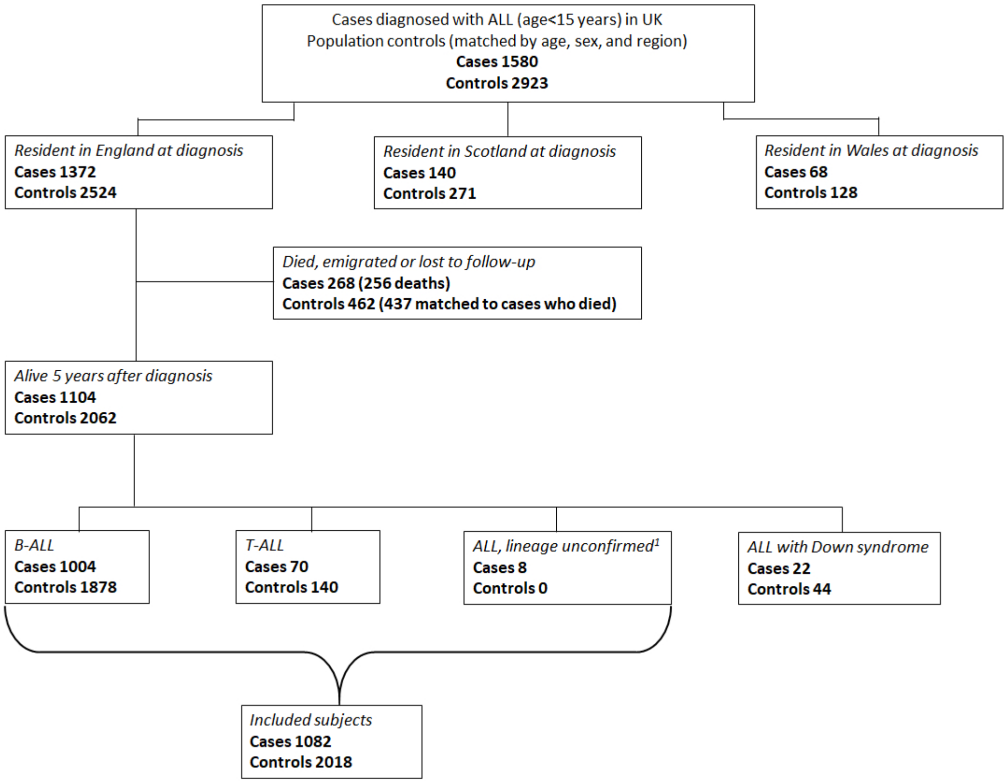

Figure 1: Flowchart showing subjects targeted in the original United Kingdom Childhood Cancer case-control study and those included in the present cohort

715x558mm (38 x 38 DPI)

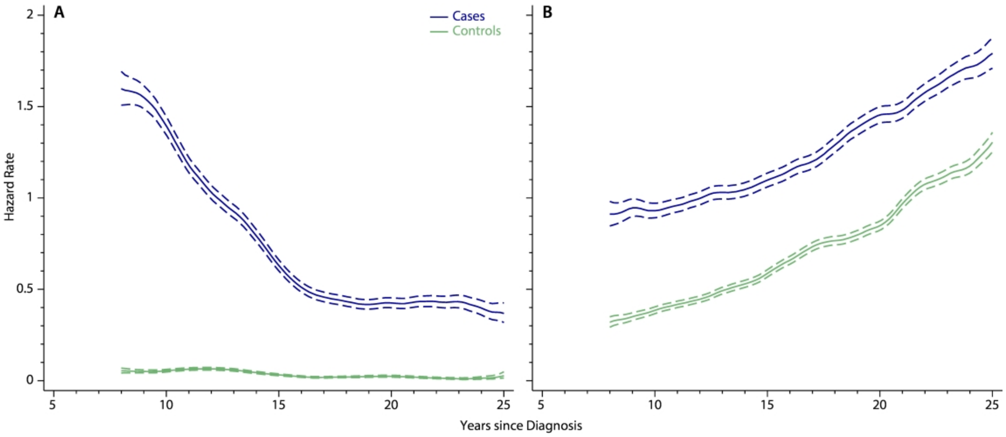

Figure 2: Outpatient activity hazard rates per year and 95% confidence intervals (dotted lines) for childhood (< 15 years) ALL 5-year survivors and their matched controls: A) paediatrics, haematology and oncology, B) all other clinical specialties

715x309mm (236 x 236 DPI)

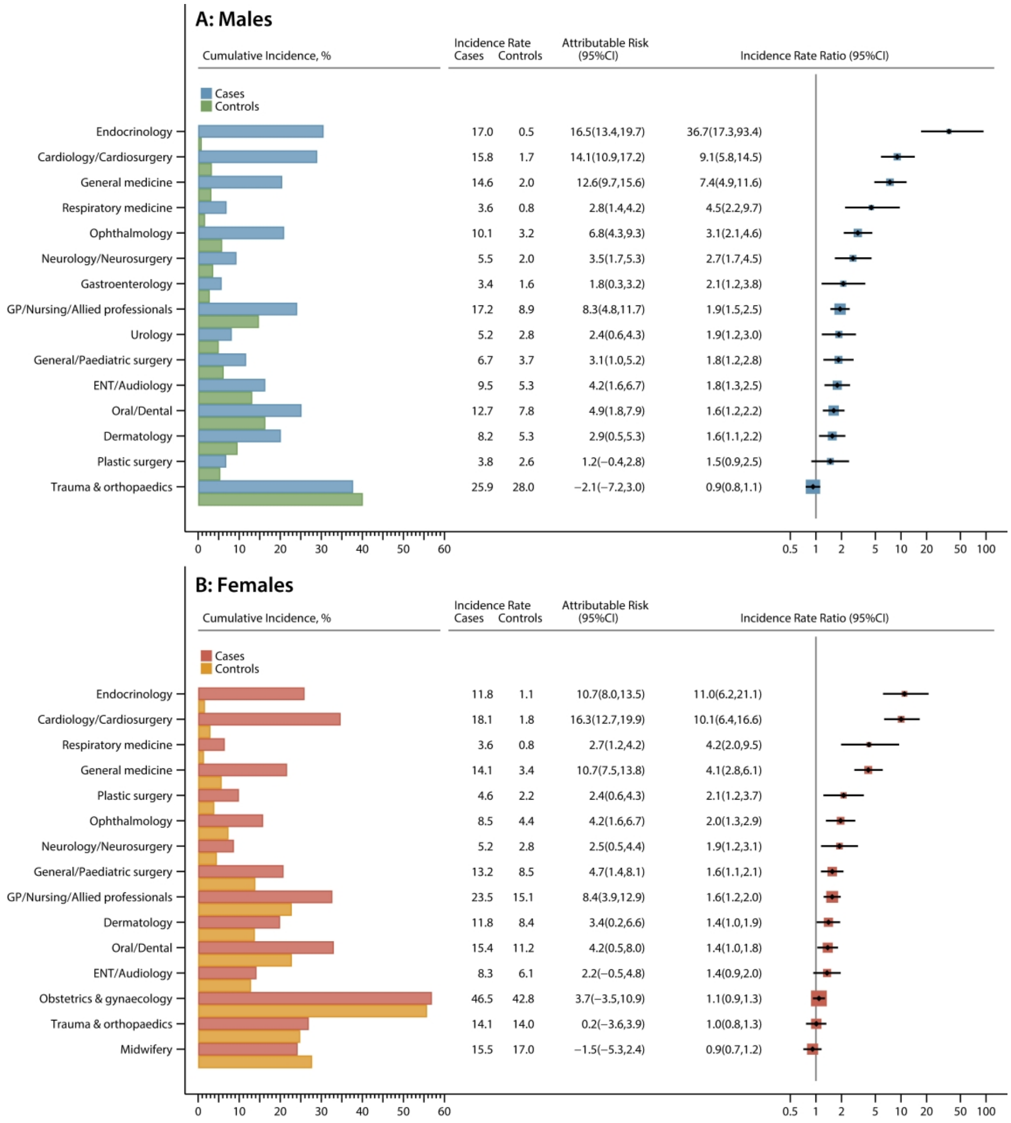

Figure 3: Cumulative incidence (%), incidence rates (per 1000 person-years), attributable risks, and incidence rate ratios for the top 15 outpatient specialties (excluding paediatrics, haematology and oncology) with two of more face-to-face speciality-specific visits in the 5 to 25 years following diagnosis (cases diagnosed <15 years, 1992-96) and their matched population controls

380x423mm (600 x 600 DPI)

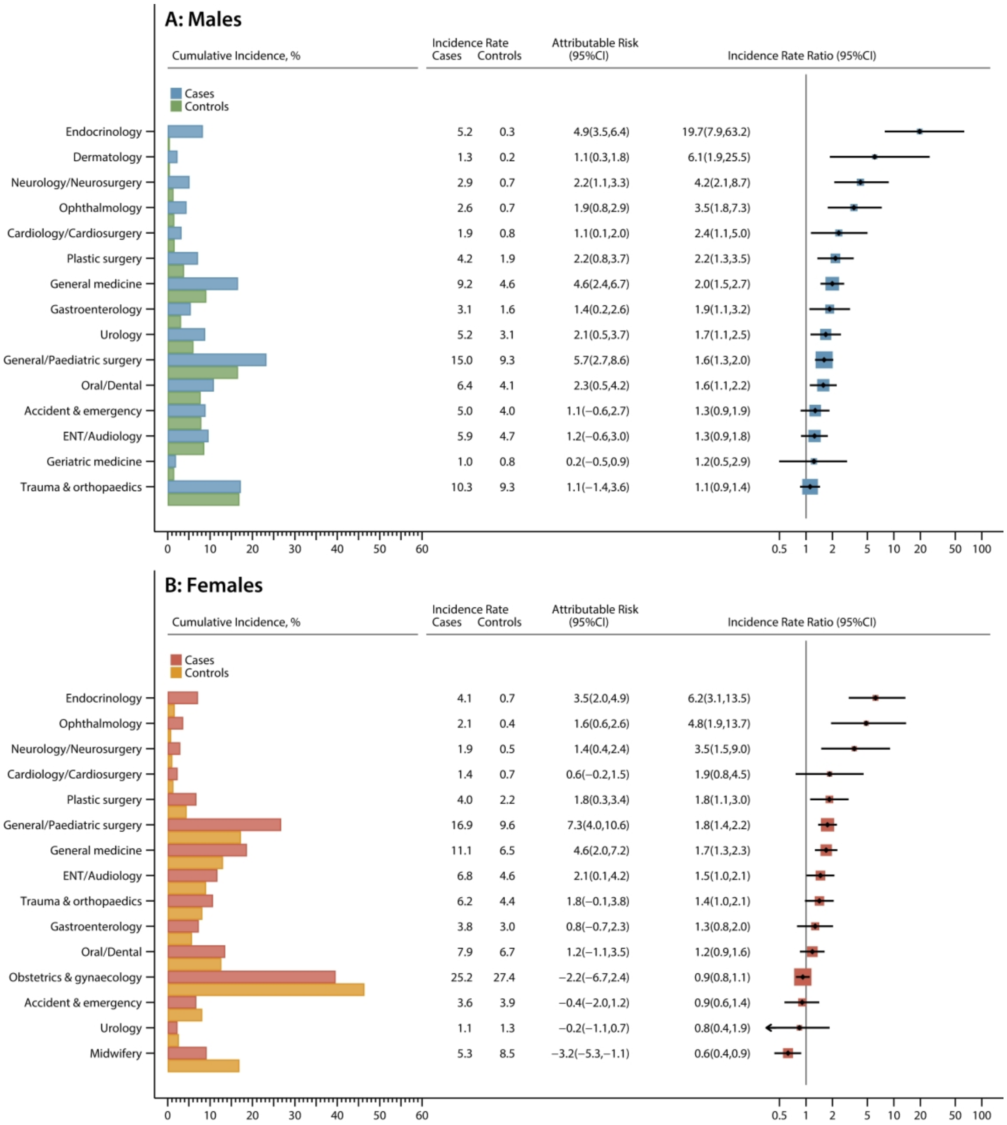

Figure 4: Cumulative incidence (%), incidence rates (per 1000 person-years) and incidence rate ratios for the top 15 inpatient specialties (excluding paediatrics, haematology and oncology) in the 5 to 25 years following diagnosis (cases diagnosed aged<15 years, 1992-96) and their matched population controls.

380x425mm (600 x 600 DPI)

Supplementary Figure 1: Hazard rates per year of activity at paediatrics, haematology and oncology among ALL cases who survived 5 years or more and their matched population controls: UKCCS, ALL diagnosed aged<15 years, 1992-96.

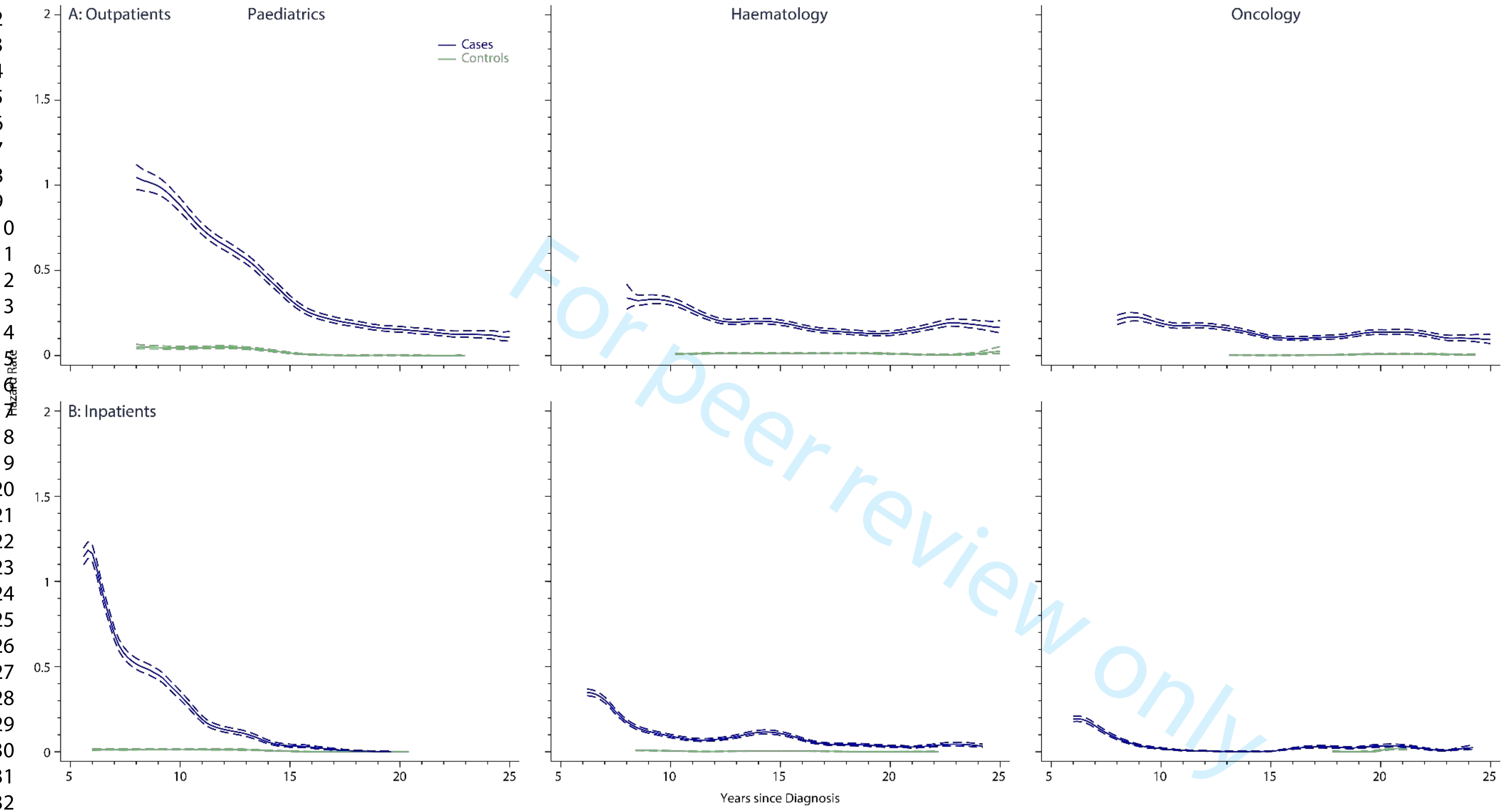

Supplementary Figure 2: Cumulative incidence, rates (per 1000 person-years), attributable risks & incidence rate ratios; top 15 outpatient specialties (excluding paediatrics, haematology & oncology) with ≥2 speciality-specific visits in the 5 to 15 years and 15 to 25 years following diagnosis (cases diagnosed <15 years, 1992-96) and their matched controls

A: Males

£15 Years

	Incidence Rate Cases	Incidence Rate Controls	Attributable Risk (95%CI)	Incidence Rate Ratio (95%CI)
Endocrinology	29.1	0.5	28.6(22.4,34.8)	61.3(20.3,303)
Cardiology/Cardiosurgery	19.0	1.1	17.9(13.0,22.9)	17.1(7.8,44.4)
General medicine	17.5	2.1	15.5(10.7,20.2)	8.5(4.6,16.9)
Ophthalmology	18.6	2.7	15.8(10.8,20.8)	6.8(3.9,12.4)
Respiratory medicine	3.6	0.6	3.0(0.8,5.1)	5.7(1.7,24.1)
Neurology/Neurosurgery	6.4	1.8	4.6(1.7,7.5)	3.6(1.7,8.3)
GP/Nursing/Allied professionals	13.9	4.0	9.8(5.5,14.2)	3.5(2.1,5.9)
Gastroenterology	2.1	0.6	1.4(−0.2,3.1)	3.3(0.8,15.2)
Oral/Dental	21.1	8.6	12.5(6.9,18.2)	2.5(1.7,3.6)
General/Paediatric surgery	8.7	3.6	5.1(1.6,8.7)	2.4(1.3,4.5)
Urology	4.5	2.3	2.3(−0.3,4.9)	2.0(0.9,4.5)
Plastic surgery	4.8	2.4	2.4(−0.3,5.0)	2.0(0.9,4.3)
ENT/Audiology	12.2	6.4	5.8(1.5,10.2)	1.9(1.2,3.1)
Dermatology	11.9	8.2	3.8(−0.6,8.2)	1.5(0.9,2.3)
Trauma & orthopaedics	38.0	38.7	−0.7(−9.6,8.1)	1.0(0.8,1.2)

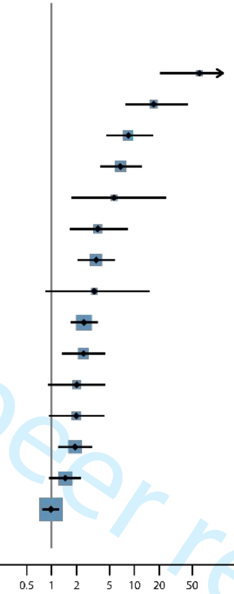

>15–25 Years

	Incidence Rate Cases	Incidence Rate Controls	Attributable Risk (95%CI)	Incidence Rate Ratio (95%CI)
Endocrinology	7.5	0.5	7.0(4.2,9.8)	16.4(5.7,64.5)
General medicine	12.1	1.9	10.2(6.5,13.8)	6.4(3.5,12.1)
Cardiology/Cardiosurgery	12.9	2.2	10.7(6.8,14.7)	5.8(3.3,10.6)
Respiratory medicine	3.5	0.9	2.6(0.8,4.5)	3.9(1.6,10.5)
Neurology/Neurosurgery	4.9	2.2	2.7(0.4,5.0)	2.2(1.1,4.3)
Gastroenterology	4.3	2.3	2.0(−0.1,4.1)	1.9(1.0,3.7)
Urology	5.8	3.2	2.6(0.0,5.1)	1.8(1.0,3.2)
Dermatology	5.3	3.1	2.3(−0.3,4.8)	1.7(0.9,3.2)
ENT/Audiology	7.4	4.5	2.8(−0.2,5.9)	1.6(1.0,2.7)
GP/Nursing/Allied professionals	20.2	12.9	7.3(2.0,12.7)	1.6(1.1,2.1)
General/Paediatric surgery	5.2	3.7	1.5(−1.0,4.1)	1.4(0.8,2.5)
Plastic surgery	3.1	2.7	0.4(−1.6,2.3)	1.1(0.5,2.3)
Ophthalmology	3.7	3.6	0.1(−2.2,2.3)	1.0(0.5,2.0)
Trauma & orthopaedics	15.1	18.0	−2.9(−8.4,2.6)	0.8(0.6,1.2)
Oral/Dental	6.1	7.3	−1.2(−4.3,1.9)	0.8(0.5,1.4)

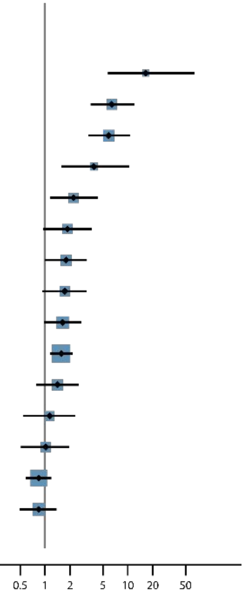

B: Females

£15 Years

	Incidence Rate Cases	Incidence Rate Controls	Attributable Risk (95%CI)	Incidence Rate Ratio (95%CI)
Endocrinology	20.0	0.7	19.2(13.7,24.7)	26.7(9.8,102)
Cardiology/Cardiosurgery	20.3	1.9	18.5(12.9,24.1)	10.8(5.4,23.8)
Respiratory medicine	5.0	0.6	4.5(1.8,7.2)	8.9(2.5,48.5)
General medicine	16.1	2.6	13.5(8.5,18.5)	6.1(3.3,12.1)
Plastic surgery	6.1	2.3	3.8(0.6,7.0)	2.7(1.2,6.1)
Dermatology	17.4	7.4	10.0(4.5,15.6)	2.4(1.5,3.7)
General/Paediatric surgery	12.8	5.7	7.1(2.4,11.8)	2.2(1.3,3.8)
GP/Nursing/Allied professionals	13.4	6.3	7.1(2.3,11.9)	2.1(1.3,3.5)
Neurology/Neurosurgery	5.0	2.6	2.3(−0.6,5.3)	1.9(0.8,4.3)
Ophthalmology	10.8	5.9	4.8(0.4,9.3)	1.8(1.1,3.1)
ENT/Audiology	10.2	6.0	4.2(−0.1,8.5)	1.7(1.0,2.9)
Oral/Dental	22.6	13.9	8.7(2.1,15.4)	1.6(1.1,2.3)
Trauma & orthopaedics	20.0	16.4	3.6(−2.9,10.1)	1.2(0.8,1.7)
Obstetrics & gynaecology	27.9	28.5	−0.6(−8.5,7.3)	1.0(0.7,1.3)
Midwifery	3.1	5.1	−1.9(−4.7,0.9)	0.6(0.3,1.4)

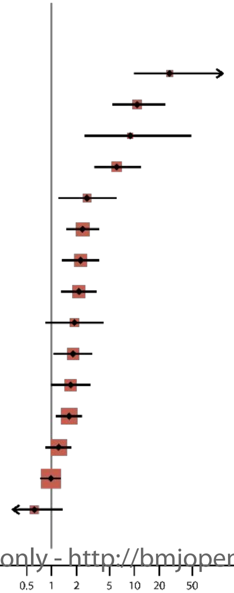

>15–25 Years

	Incidence Rate Cases	Incidence Rate Controls	Attributable Risk (95%CI)	Incidence Rate Ratio (95%CI)
Cardiology/Cardiosurgery	16.1	1.7	14.4(9.7,19.0)	9.3(4.9,18.7)
Endocrinology	5.9	1.3	4.6(1.9,7.2)	4.5(2.0,10.7)
General medicine	12.4	4.0	8.4(4.3,12.4)	3.1(1.9,5.2)
Respiratory medicine	2.5	1.0	1.5(−0.2,3.2)	2.4(0.9,7.0)
Ophthalmology	6.9	3.2	3.7(0.7,6.6)	2.1(1.2,3.9)
Neurology/Neurosurgery	5.4	2.8	2.5(−0.1,5.2)	1.9(1.0,3.7)
Plastic surgery	3.6	2.1	1.5(−0.7,3.6)	1.7(0.8,3.7)
GP/Nursing/Allied professionals	32.6	22.3	10.3(2.9,17.6)	1.5(1.1,1.9)
General/Paediatric surgery	13.5	10.7	2.8(−1.8,7.5)	1.3(0.9,1.9)
Obstetrics & gynaecology	66.6	58.0	8.6(−3.7,21.0)	1.1(0.9,1.4)
ENT/Audiology	6.9	6.2	0.6(−2.6,3.9)	1.1(0.6,1.8)
Oral/Dental	9.8	9.1	0.7(−3.4,4.9)	1.1(0.7,1.7)
Midwifery	25.3	26.6	−1.3(−7.8,5.2)	1.0(0.7,1.2)
Dermatology	7.4	9.1	−1.8(−5.4,1.9)	0.8(0.5,1.3)
Trauma & orthopaedics	9.6	12.1	−2.5(−6.8,1.8)	0.8(0.5,1.2)

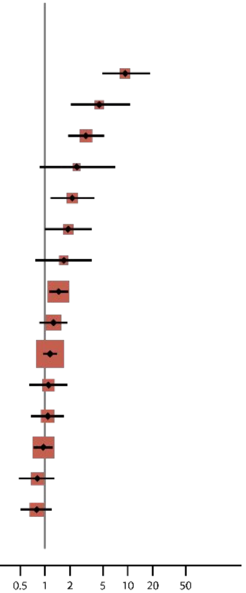

For peer review only

STROBE Statement—Checklist of items that should be included in reports of ***cohort studies***

	Item No	Recommendation	Page No
**Title and abstract**	1	(*a*) Indicate the study's design with a commonly used term in the title or the abstract	2&3
		(*b*) Provide in the abstract an informative and balanced summary of what was done and what was found	2
**Introduction**			
Background/rationale	2	Explain the scientific background and rationale for the investigation being reported	3
Objectives	3	State specific objectives, including any prespecified hypotheses	3
**Methods**			
Study design	4	Present key elements of study design early in the paper	3-4
Setting	5	Describe the setting, locations, and relevant dates, including periods of recruitment, exposure, follow-up, and data collection	3-4
Participants	6	(*a*) Give the eligibility criteria, and the sources and methods of selection of participants. Describe methods of follow-up	3-4
		(*b*) For matched studies, give matching criteria and number of exposed and unexposed	3-4; Fig 1
Variables	7	Clearly define all outcomes, exposures, predictors, potential confounders, and effect modifiers. Give diagnostic criteria, if applicable	3-5
Data sources/ measurement	8*	For each variable of interest, give sources of data and details of methods of assessment (measurement). Describe comparability of assessment methods if there is more than one group	4-5
Bias	9	Describe any efforts to address potential sources of bias	3-4
Study size	10	Explain how the study size was arrived at	3-4
Quantitative variables	11	Explain how quantitative variables were handled in the analyses. If applicable, describe which groupings were chosen and why	3-4
Statistical methods	12	(*a*) Describe all statistical methods, including those used to control for confounding	3-4
		(*b*) Describe any methods used to examine subgroups and interactions	NA
		(*c*) Explain how missing data were addressed	Fig 1
		(*d*) If applicable, explain how loss to follow-up was addressed	Fig 1
		(*e*) Describe any sensitivity analyses	NA
**Results**			
Participants	13*	(a) Report numbers of individuals at each stage of study—eg numbers potentially eligible, examined for eligibility, confirmed eligible, included in the study, completing follow-up, and analysed	Tab 1
		(b) Give reasons for non-participation at each stage	3
		(c) Consider use of a flow diagram	Fig 1
Descriptive data	14*	(a) Give characteristics of study participants (eg demographic, clinical, social) and information on exposures and potential confounders	Tab 1
		(b) Indicate number of participants with missing data for each variable of interest	3-4; Fig 1
		(c) Summarise follow-up time (eg, average and total amount)	Tab 1
Outcome data	15*	Report numbers of outcome events or summary measures over time	5-6; Tab 2 Fig 1

For peer review only - http://bmjopen.bmj.com/site/about/guidelines.xhtml

Main results	16	(*a*) Give unadjusted estimates and, if applicable, confounder-adjusted estimates and their precision (eg, 95% confidence interval). Make clear which confounders were adjusted for and why they were included	5-6
		(*b*) Report category boundaries when continuous variables were categorized	5-6
		(*c*) If relevant, consider translating estimates of relative risk into absolute risk for a meaningful time period	Figs 3-4
Other analyses	17	Report other analyses done—eg analyses of subgroups and interactions, and sensitivity analyses	6
**Discussion**			
Key results	18	Summarise key results with reference to study objectives	6-7
Limitations	19	Discuss limitations of the study, taking into account sources of potential bias or imprecision. Discuss both direction and magnitude of any potential bias	6-7
Interpretation	20	Give a cautious overall interpretation of results considering objectives, limitations, multiplicity of analyses, results from similar studies, and other relevant evidence	2; 6-7
Generalisability	21	Discuss the generalisability (external validity) of the study results	7
**Other information**			
Funding	22	Give the source of funding and the role of the funders for the present study and, if applicable, for the original study on which the present article is based	7

*Give information separately for exposed and unexposed groups.

Note: An Explanation and Elaboration article discusses each checklist item and gives methodological background and published examples of transparent reporting. The STROBE checklist is best used in conjunction with this article (freely available on the Web sites of PLoS Medicine at http://www.plosmedicine.org/, Annals of Internal Medicine at http://www.annals.org/, and Epidemiology at http://www.epidem.com/). Information on the STROBE Initiative is available at http://www.strobe-statement.org.

For peer review only

For peer review only - http://bmjopen.bmj.com/site/about/guidelines.xhtml

BMJ Open

Excess morbidity and mortality among survivors of childhood acute lymphoblastic leukaemia: 25 years of follow-up from the United Kingdom Childhood Cancer Study (UKCCS) population-based matched cohort

Journal:	*BMJ Open*
Manuscript ID	bmjopen-2021-056216.R2
Article Type:	Original research
Date Submitted by the Author:	31-Jan-2022
Complete List of Authors:	Kane, Eleanor; University of York,<br>Kinsey, Sally; Leeds Teaching Hospitals NHS Trust<br>Bonaventure, Audrey; INSERM, Epidemiology of childhood and adolescent cancers team<br>Johnston, Tom; University of York, Department of Health Sciences<br>Simpson, Jill; University of York, Department of Health Sciences<br>Howell, Debra; University of York, Health Sciences<br>Smith, Alexandra; University of York, Health Sciences<br>Roman, Eve; University of York, Health Sciences
**Primary Subject Heading**:	Epidemiology
Secondary Subject Heading:	Public health
Keywords:	Leukaemia < HAEMATOLOGY, Paediatric oncology < ONCOLOGY, Epidemiology < ONCOLOGY, PUBLIC HEALTH

SCHOLARONE™
Manuscripts

I, the Submitting Author has the right to grant and does grant on behalf of all authors of the Work (as defined in the below author licence), an exclusive licence and/or a non-exclusive licence for contributions from authors who are: i) UK Crown employees; ii) where BMJ has agreed a CC-BY licence shall apply, and/or iii) in accordance with the terms applicable for US Federal Government officers or employees acting as part of their official duties; on a worldwide, perpetual, irrevocable, royalty-free basis to BMJ Publishing Group Ltd ("BMJ") its licensees and where the relevant Journal is co-owned by BMJ to the co-owners of the Journal, to publish the Work in this journal and any other BMJ products and to exploit all rights, as set out in our licence.

The Submitting Author accepts and understands that any supply made under these terms is made by BMJ to the Submitting Author unless you are acting as an employee on behalf of your employer or a postgraduate student of an affiliated institution which is paying any applicable article publishing charge ("APC") for Open Access articles. Where the Submitting Author wishes to make the Work available on an Open Access basis (and intends to pay the relevant APC), the terms of reuse of such Open Access shall be governed by a Creative Commons licence – details of these licences and which Creative Commons licence will apply to this Work are set out in our licence referred to above.

Other than as permitted in any relevant BMJ Author's Self Archiving Policies, I confirm this Work has not been accepted for publication elsewhere, is not being considered for publication elsewhere and does not duplicate material already published. I confirm all authors consent to publication of this Work and authorise the granting of this licence.

For peer review only

Excess morbidity and mortality among survivors of childhood acute lymphoblastic leukaemia: 25 years of follow-up from the United Kingdom Childhood Cancer Study (UKCCS) population-based matched cohort

Eleanor Kane[1], Sally Kinsey [1,2], Audrey Bonaventure[3], Tom Johnston[1], Jill Simpson[1], Debra Howell [1] Alexandra Smith[1], Eve Roman [1,4]

[1] Department of Health Sciences, University of York, UK

[2] Department of Paediatric Haematology, Leeds Teaching Hospital's NHS Trust, Leeds, UK

[3] CRESS, Université de Paris, INSERM UMR-1153, Epidemiology of childhood and adolescent cancers team, Villejuif, France

[4] Correspondence to: eve.roman@york. ac.uk, Epidemiology and Cancer Statistics Group, Department of Health Sciences, University of York, York, YO10 5DD, UK

Authors' names and positions

Eleanor Kane (0000-0002-7438-9982), Senior research fellow

Sally Kinsey, (0000-0002-5777-3333), Professor of paediatric haematology

Audrey Bonaventure (0000-0001-6665-8145), Cancer Researcher/Senior Visiting Research Fellow

Tom Johnston (0000-0002-5204-0966), Research fellow

Jill Simpson, (0000-0003-3156-8822), Visiting Research fellow

Debra Howell (0000-0002-7521-7402), Senior research fellow

Alexandra Smith (0000-0002-1111-966X), Professor in cancer epidemiology

Eve Roman (0000-0001-7603-3704), Professor of epidemiology

Word Count (main text) = 3235

For peer review only - http://bmjopen.bmj.com/site/about/guidelines.xhtml

Abstract

Objectives

To examine morbidity and mortality among teenagers and young adults (TYAs) previously diagnosed with acute lymphoblastic leukaemia (ALL) in childhood, and compare to the general TYA population.

Design

National population-based sex- and age-matched case-control study converted into a matched-cohort, with follow-up linkage to administrative healthcare databases.

Setting

The study population comprised all children (0-14 years) registered for primary care with the National Health Service (NHS) in England 1992-1996.

Participants

1082 5-year survivors of ALL diagnosed <15 years (1992-1996) and 2018 unaffected individuals; followed-up to 15/03/2020.

Main outcome measures

Associations with hospital activity, cancer, and mortality were assessed using incidence rate ratios (IRR) and differences.

Results

Mortality in the ALL-cohort was 20 times higher than in the comparison-cohort (Rate Ratio 21.3, 95% Confidence Interval 11.2-45.6), and cancer incidence 10 times higher (IRR 9.9 95% CI 4.1-29.1). Hospital activity was increased for many clinical specialties, the strongest associations being for endocrinology; outpatient IRR 36.7, 95% CI 17.3-93.4 and inpatient 19.7, 95% CI 7.9-63.2 for males, and 11.0, 95% CI 6.2-21.1 and 6.2 95% CI 3.1-13.5 respectively for females. Notable excesses were also evident for cardiology, neurology, ophthalmology, respiratory medicine and general medicine. Males were also more likely to attend gastroenterology, ENT (ear, nose and throat), urology, and dermatology; while females were more likely to be seen in plastic surgery and less likely in midwifery.

Conclusions

Adding to excess risks of death and cancer, survivors of childhood ALL experience excess outpatient and inpatient activity across their TYA years; which is not related to routine follow-up monitoring. Involving most clinical specialties, associations are striking; showing no signs of diminishing over time. Recognising that all survivors are potentially at risk of late treatment-associated effects, our findings underscore the need to take prior ALL diagnosis into account when interpreting seemingly unrelated symptoms later in life.

For peer review only

ARTICLE SUMMARY

Strengths and limitations of this study

- With minimal selection bias and loss to follow-up, findings from this national study are based on linked hospital data, not self-report
- Ensuring homogeneity of treatment, diagnostic dates are contemporaneous with the national UKALL XI RCT, within which 90.5% of those who survived were enrolled
- The individually sex- and age-matched general population controls had a similar deprivation distribution to cases, enabling robust baseline effect measures to be calculated across the 25-year follow-up period
- Analyses are constrained by the fact that hospital episodes statistics are primarily collected for administrative and clinical purposes, and not for research

Funding

This work was supported by Blood Cancer UK (grant number 15037) and Cancer Research UK (grant number 29685). AB is supported by a fellowship from the Fondation ARC pour la Recherche sur le Cancer (PDF20190508759). The funders had no role in considering the study design or in the collection, analysis, interpretation of data, writing of the manuscript, or the decision to submit the article for publication.

Competing interests

None of the authors have any competing interests

Data sharing agreement

No additional data available

For peer review only

For peer review only - http://bmjopen.bmj.com/site/about/guidelines.xhtml

Introduction

Comprising around 40% of all cancers diagnosed in children before 15 years of age, acute lymphoblastic leukaemia (ALL) is the commonest paediatric malignancy in high income countries. Epitomizing one of the major therapeutic success stories of the last 50 years, five-year overall survival for childhood ALL is now around 90% in high-income settings. (1) As a result, the global prevalence of survivors is increasing year-on-year; and will continue to do so as treatments improve, populations age, costs fall, and new clinical collaborations between high- and low-income countries are forged. (2)

In contrast to many other childhood cancers, ALL treatment is prolonged, with current protocols typically administering chemotherapy in phases extending over a 2- to 3-year period. (3) That children who survive ALL tend to have higher mortality and morbidity in later life, including subsequent cancers and a wide range of other therapy-related conditions (Table 1), is established. (4–7) With respect to the latter, however, much of the information on late effects among survivors has come either from analyses of hospitalizations or from questionnaires completed by consenting individuals.(8–17) As is evident from the studies summarized in Table 1 (which includes those that examined all leukaemias combined and those that also included young adults), less attention has been paid to chronic conditions that do not require hospital admission, (7,18–20) and to morbidity patterns that change over time. (7,12,21)

The longitudinal data examined in this report contain information on hospital outpatient attendances, as well as inpatient episodes, cancer registrations, and deaths. Adding significantly to the existing knowledge base, a large cohort of children (<15 years) diagnosed with ALL in England during 1992-6 and their individually sex- and age-matched population controls have been tracked for up to 25 years through their teenage and young adult (TYA) years. Increasing the power to detect effects should they exist, this diagnostic time period (1992-6) coincides with the UKALL XI randomised controlled trial (RCT) (22); the last very intensive UK RCT prior to the era of clinical investigations enabling reduction in therapy for patients with rapid early response to treatment. (23)

Methods

Data are from the United Kingdom Childhood Cancer Study (UKCCS); a national population-based case-control study established in the 1990s to investigate potential causes of childhood cancer that has now been converted to a population-based cohort study to examine survivorship. (24) Full details of the UKCCS's original methods and ethical permissions have been published. (25–27) Briefly, the study population comprised all children (0-14 years) registered for primary care with the National Health Service (NHS) 1992-1996 (England and Wales) and 1991-1995 (Scotland). All children newly diagnosed with cancer (cases) were ascertained via proactive notification systems established in all treatment centres across England, Scotland and Wales; and each child whose parents agreed to be interviewed (87%; all cancers combined) was individually matched on sex, date of birth and region of residence to up to 10 randomly selected controls. The general practitioners (GPs) of the first two controls identified ("first-choice controls") were approached and, with their permission, the parents of the children were contacted and asked to participate in the study. Overall, 72% of first-choice control families participated; but if the GP refused permission to contact

For peer review only - http://bmjopen.bmj.com/site/about/guidelines.xhtml

the parents, or the parents themselves declined, the next control on the list was selected, and so on until two control families agreed. (25–27) For methodological purposes (26,27), details of all non-participating cases and controls were retained in the UKCCS registration database.

With a view to examining childhood cancer sequelae, the UKCCS in England now operates on a legal basis that permits information to be obtained from NHS administrative healthcare records without explicit consent; and all registered cases and first-choice controls, along with replacement controls whose parents participated, are now being prospectively tracked by NHS Digital (https://digital.nhs.uk/) via linkage to nationwide (England) information on deaths, cancer registrations, and Hospital Episode Statistics (HES; inpatient day and overnight admissions, and outpatient appointments and visits). Mortality, cancer incidence, and secondary healthcare utilization of children diagnosed with ALL who survived for 5 years or more is compared here to that of individually sex- and age-matched general-population controls using a matched population-based cohort design.

Data and statistical analysis

The number of ALL cases diagnosed in the original study and their corresponding first-choice controls, as well as subsequent deaths and losses to follow-up occurring in England within 5 years of ALL diagnosis/pseudo-diagnosis are shown in Figure 1. Overall, 1574/1580 (99.6%) cases and 2918/2923 (99.8%) of their first-choice controls were successfully traced and linked to national administrative databases. During the study period (1992 to 1996), 1372/1580 children were diagnosed with ALL in England, and the parents of 92% (1262/1372) of these agreed to participate, each of whom had two sex-and age-matched first-choice controls selected (n=2524).

In the original case-control study, each unaffected control child was assigned a pseudo-diagnosis date that corresponded to their matched case's exact age at diagnosis; and the dates of diagnosis/pseudo-diagnosis now mark the start of follow-up for the matched cohorts. Of the 1104 ALL cases (80.5%) diagnosed in England who survived for 5 years or more, 1004 (90.9%) had B-ALL and 70 (6.3%) T-ALL. The cell lineage of the remaining eight ALL survivors is unknown; they were not entered into a clinical trial and the parents did not participate in the original study (hence no controls were selected). The 22 surviving case children with Down syndrome (no first-choice control had Down syndrome) and their matched controls were excluded from the survivorship analysis because Down syndrome is a well-established risk factor for ALL, and it is also associated with a range of other morbidities.

For all of the 1082 cases and 2018 controls included in this report (Figure 1), linked healthcare data were available on cancer registrations and deaths up to March 2020, inpatient HES from April 1997 to March 2019, and outpatient HES from April 2003 to March 2019. Follow-up ended either at the date of death, 25 years post-diagnosis, or end date for hospital data. For controls, follow-up could also end when their matched case died. Migrations out of England and any subsequent returns were accounted for, and since there is no risk of a hospital visit during an admission, days spent in hospital were also excluded from the follow-up time.

Although the International Classification of Diseases (ICD) is used to code deaths and cancers, it is not routinely used in outpatient HES, and the ICD codes in inpatient HES are not easily assigned to specific events. Each HES episode (inpatient and outpatient) is, however, assigned to an individual consultant whose clinical specialty categorization is recorded; and these categorizations were used in the HES analyses presented here. Firstly, since childhood ALL survivors are routinely monitored via outpatient visits to paediatrics, haematology and/or oncology in the UK, HES attendances to these clinical specialties were separated from those to "other specialties" at the outset (Figure 2).

For peer review only - http://bmjopen.bmj.com/site/about/guidelines.xhtml

For peer review only

All subsequent specialty-specific analyses presented in this report were restricted to the "other specialties" dataset. As in previous reports (28,29), a single inpatient admission and/or two or more specialty-specific face-to-face outpatient attendances were used to indicate potential health issues. Exact time-to-event methods were used to plot estimates of the hospital visit hazard rates before calculating cumulative incidence (considering death as a competing risk), and risks (rate ratios and attributable risks) associated with specific clinical specialties. Hospital visit hazard rates (all visits) are estimated as number of visits per person-year while incidence rates of ever visiting specific clinical specialties are calculated per 1000 person-years; for the latter, follow-up time was truncated at the first hospital visit to the specialty. All specialties were analysed, and findings for the 15 specialties with the highest cumulative incidences among the cases are presented. To analyse this prospective matched cohort study which included virtually all cases nationally, methods analogous to those employed in other studies of a similar design were used (9,28,29). Importantly, cases and their randomly sampled controls were identical with respect to sex, age and socioeconomic status at baseline (27), and remained so 5-years from diagnosis. With age, sex and socioeconomic status effectively controlled for in the study design, adjusting in the analyses will not change the risk estimates, and as such, rates, rate ratios and attributable risks derived using exact methods are presented without any adjustments. All analyses were conducted using Stata 16.1.

Patient Public Involvement and Engagement (PPIE)

The original case-control study was established over 30 years ago, and did not benefit from formal links with patient/user groups. Members of the CCLG (Children's Cancer and Leukaemia Group, previously the UK Children's Cancer Study Group) were, however, fully involved throughout. More recently with respect to the present research, the acceptability of using patient data without explicit consent (as is the case in this report) has been positively discussed with patient groups and clinicians; and the study website (www.UKCCS.org) contains information about ongoing research activities and fair processing. It is our intention to establish a PPIE group to input into future research when the COVID pandemic permits.

Results

The characteristics of individuals in the original case-control study and in the 5-year survivor cohorts are shown in Table 2. As expected, the 290 (21%) children with ALL who died within five years of diagnosis were more likely to have had T-cell disease (χ^2=44.0, p<0.001), have been infants or older children (χ^2=100.9, p<0.001), and were less likely to have participated in a clinical trial (χ^2=11.6, p=0.001). Nevertheless the 5-year survivors and their controls remained similar with respect to sex (χ^2=0.0002, p=0.99) and age (χ^2=0.35, p=0.95) as well as deprivation (IMD - Index of Multiple Deprivation; χ^2=0.32, p=0.57). Tracking children from 5 years after ALL diagnosis to death or 25 years from diagnosis, this report's primary focus is teenage and young adult (TYA) survivors; as they were aged around 9 years at the start of follow-up (median age 9.4 years, InterQuartile Range 8.0-12.1 years) and almost 30 by the end (median attained age 29.6 years, IQR 27.3-32.1). Between these ages, again as expected, the 5-year ALL survivors continued to experience more deaths (cumulative mortality 10.7%, 95% Confidence Interval 9.0-12.8) than their general population counterparts (cumulative mortality 0.6%, 95% CI 0.3-1.1), yielding a mortality rate ratio of 21.3 (95% CI 11.2-45.6); and were more likely to have a subsequent cancer registration (non-ALL malignancy Incidence Rate Ratio 9.9, 95% CI 4.1-29.1), although the numbers are small (32 *versus* 6).

Childhood ALL survivors are regularly monitored throughout their TYA years either in paediatrics, haematology or oncology, as is evidenced in Figure 2A which shows the outpatient activity of 5-year ALL survivors and their corresponding controls for these three clinical specialties combined

For peer review only - http://bmjopen.bmj.com/site/about/guidelines.xhtml

(specialty-specific outpatient and inpatient data are shown in Supplementary Figure 1). At around one visit in 20 years, hospital activity among population controls is very low, and varies little over time. By contrast, for ALL survivors, 8 years after diagnosis outpatient activity is around 35 times higher than the background rate, falling 10 years later to around 0.4 visits per case per year (Figure 2A). In all other clinical specialties combined, outpatient activity among childhood ALL survivors are also consistently higher than expected; hazard rates in both cohorts increasing over time at the same steady rate (Figure 2B).

More information on hospital activity is presented for the top 15 specialties (excluding paediatrics, haematology and oncology) for males and females separately in Figure 3 (outpatients) and Figure 4 (inpatients). In all figures, cumulative incidence frequencies are on the left and incidence rates on the right; with the ordering of specialties determined by the magnitude of the incidence rate ratio (IRR). Most specialties are associated with high relative and attributable risks (inpatient and outpatient). Furthermore, although the magnitude of the estimates often varies between males and females, the overall pattern is broadly similar. In both sexes, for example, the strongest effects are seen for endocrinology; the IRRs for outpatients and inpatients being 36.7 (95% CI 17.3-93.4) and 19.7 (95% CI 7.9-63.2) respectively for males, and 11.0 (95% CI 6.2-21.1) and 6.2 (95% CI 3.1-13.5) for females. Notable excesses (relative and attributable) are also evident for cardiology, neurology, ophthalmology, respiratory medicine and general medicine, for both genders. Male survivors were also more likely than their peers to attend gastroenterology, ENT (ear, nose and throat), urology, and dermatology; while female survivors were twice as likely as their controls to be referred to plastic surgery. Interestingly, no case-control differences are evident for trauma and orthopaedics, which has the highest background cumulative incidence in males. Likewise, among females, no differences were detected for obstetrics and gynaecology; however, it is notable that activity is lower than expected in midwifery (outpatient IRR 0.9, 95% CI 0.7-1.2; inpatient IRR 0.6, 95% CI 0.4-0.9).

Detailed inspection of the HES data revealed no evidence of specialty-specific changes in either relative or attributable risks over the 20-year time frame (Supplementary Figure 2); possible exceptions being falls in ranking for ophthalmology among males and dermatology among females. Furthermore, no differences between B- and T-cell survivors with respect to any of the outcomes were evident, although the number with T-cell disease was comparatively small (n=70).

For peer review only

Discussion

Including data on more than 1000 survivors of childhood ALL and twice as many age- and sex-matched general population controls, this national record-linkage study found that survivors continued to experience large excesses in mortality and morbidity throughout their teenage and young adult years. With hospital activity being higher than expected in specialties covering most organ and tissue systems, survivors were more than twice as likely to fall under the care of endocrinology, cardiology, respiratory medicine, ophthalmology, neurology and/or gastroenterology specialists. Moreover, aside from a gradual decline in visits to clinical specialties involved in follow-up monitoring (paediatrics, haematology and oncology), there was little indication of activity returning to general population levels up to 25 years after the initial ALL diagnosis. Among females, it is also notable that midwifery outpatient attendances were not increased, with admissions being lower than expected.

Our study, which uses linked rather than self-reported data and population controls, is one of only two (18) to separate secondary care activity associated with cancer follow-up from activity

For peer review only - http://bmjopen.bmj.com/site/about/guidelines.xhtml

associated with other morbidities; finding that survivors not only experienced higher rates of admission but also higher rates of outpatient visits, neither of which abated over their TYA years. Reported less often are the types of conditions underlying these associations, with four studies assessing risks by organ system (9,13,15,18). Survivors in these studies were diagnosed over a wider period than the study reported here, and had median follow-up times that were shorter; 9 to 17 years from diagnosis, compared to 25 years here. These studies found that survivors were at increased risk of being hospitalised with endocrine or infectious diseases, as well as diseases involving the nervous, circulatory, skin, digestive, genitourinary, respiratory or musculoskeletal systems. With the information source mostly being inpatient records, reports using data from other healthcare settings are sparse (7,18) and not specific to survivors of ALL (19,20). Importantly, while our findings are broadly consistent with these studies, our methods also highlight excesses in ophthalmology, ENT (ear, nose and throat), and oral/dental specialties, as well as decreases in midwifery.

Ensuring homogeneity of treatment, the original study's diagnostic dates were contemporaneous with the national UKALL XI randomized controlled trial (RCT), within which 90.5% of UKCCS 5-year survivors were enrolled (22,30). This comparatively high-intensity RCT was the last UK childhood ALL trial prior to the stratification of patient treatment based on early bone marrow response to induction treatment (measured by percentage of leukaemia cells in the marrow) (23,31). Information on UKALL XI's scheduling of drugs is provided in the report by Hann and colleagues (22), several of which have been implicated, either singly or combined, in therapy-related effects (Table 1). It is, however, important to note that several of the UKALL XI drugs are no longer used (e.g. etoposide, 6-thioguanine) or are used more sparingly (e.g. daunorubicin, cyclophosphamide, corticosteroids), and that cranial irradiation is no longer routinely administered, being reserved for infrequent particular indications (32). Hence, the nature of the UKCCS cohort means that it is well placed to provide baseline data against which to evaluate the potential impact of treatment de-escalation and other protocol modifications that occurred in later years. Other major strengths include its national coverage, completeness of case ascertainment, and large in-built sex- and age-matched general population comparator that is similarly distributed with respect to region and area-based deprivation distributions (IMD - Index of Multiple Deprivation) (25–27); the latter providing the most appropriate comparator against which to evaluate the health of the ALL cohort, and obviating the need to rely on published national rates, as has mostly been the case in the UK thus far (10,15,33–35).

With regard to weaknesses, the lack of data from other parts of the NHS, including primary care and adolescent psychosocial services, is an obvious deficiency that currently affects all UK record-linkage studies of the type described here. The relative paucity of information on psychological morbidities is particularly relevant to childhood cancer survivors, who are known to be at increased risk of a number of such disorders (4,36,37). Missing information within the HES datasets is also an issue; diagnostic data, for example, is largely absent from outpatient HES. Furthermore, although steps to mitigate surveillance bias were taken by requiring at least two face-to-face outpatient visits to a given specialty, we cannot rule out the possibility that childhood cancer survivors were more likely to be referred to secondary care than their peers. With virtually complete linkage to existing national databases established, our future research will, however, continue to monitor the health of cancer survivors across their life-span. As the cohort matures and the number of events increase, this will include more detailed examination of second malignancies, as well as factors potentially associated with ALL survival and/or outcome, such as socioeconomic status and treatment information (e.g. risk strata, and relapse and/or transplant therapy).

For peer review only - http://bmjopen.bmj.com/site/about/guidelines.xhtml

Examining data in the broader context of symptomology based on on-going medical referrals, this report focuses on the health of individuals who survived five years or more following a diagnosis of ALL as a child in the early 1990s; presenting an overview for all specialists who may come across unusual symptoms, signs and issues in those having had treatment for ALL at a young age. Future work to specifically address longer-term health in relation to intensities and modifications of treatment including: central nervous system radiation (and dose), numbers of intensification blocks, particular drugs (e.g. corticosteroids, cyclophosphamide, anthracyclines, etoposide, thioguanine) as well as early relapse and haematopoietic stem cell transplantation conditioning is planned. Thus far, adding to a large excess risk for death and cancer, we found that survivors experienced excess outpatient and inpatient activity across their teenage and young adult years, which was not related to routine follow-up monitoring. Involving most clinical specialties, the observed associations are striking; showing no signs of diminishing over the first 25 years of follow-up, the main exception is the comparatively low midwifery contact seen among female ALL survivors. All survivors of ALL are at increased risk of late effects from treatment, and our findings underscore the need to take prior ALL diagnosis into account when interpreting seemingly unrelated symptoms in later life, which currently might not be recognized by specialties outside haemato-oncology. As importantly, there is also a need for provision of information to survivors themselves to encourage them to declare previous treatment for ALL as a child when they access healthcare.

Ethical approval: The United Kingdom Childhood Cancer Study (UKCCS) has ethics approval from the Yorkshire & The Humber-Leeds West Research Ethics Committee (reference 18/YH/0135) and exemption from Section 251 of the Health & Social Care Act (reference 18/CAG/0066).

Contributors: ER, SK, and JS contributed to the design of the original study; and ER and EK instigated the present investigation and prepared the report. EK conducted the analyses, and all authors (ER, EK, SK, AB, TJ, JS, DH, AS) were involved in the development, reviewing, and approving the final manuscript. ER is the principal investigator and guarantor. The corresponding author (ER) attests that all listed authors meet the authorship criteria and that no others meeting the criteria have been omitted.

For peer review only

For peer review only - http://bmjopen.bmj.com/site/about/guidelines.xhtml

References

1. Bonaventure A, Harewood R, Stiller CA, Gatta G, Clavel J, Stefan DC, et al. Worldwide comparison of survival from childhood leukaemia for 1995–2009, by subtype, age, and sex (CONCORD-2): a population-based study of individual data for 89 828 children from 198 registries in 53 countries. The Lancet Haematology. 2017 May 1;4(5):e202–17.

2. The European Society for Paediatric Oncology. The SIOPE Strategic Plan. A European Cancer Plan for Children and Adolescents. [Internet]. SIOP Europe; 2015 Sep. Available from: https://siope.eu/european-strategic-plan/

3. Teachey DT, Hunger SP, Loh ML. Optimizing therapy in the modern age: differences in length of maintenance therapy in acute lymphoblastic leukemia. Blood. 2021 Jan 14;137(2):168–77.

4. Chow EJ, Ness KK, Armstrong GT, Bhakta N, Yeh JM, Bhatia S, et al. Current and coming challenges in the management of the survivorship population. Semin Oncol. 2020;47(1):23–39.

5. Landier W, Skinner R, Wallace WH, Hjorth L, Mulder RL, Wong FL, et al. Surveillance for Late Effects in Childhood Cancer Survivors. J Clin Oncol. 2018 Jul 20;36(21):2216–22.

6. Norsker FN, Pedersen C, Armstrong GT, Robison LL, McBride ML, Hawkins M, et al. Late Effects in Childhood Cancer Survivors: Early Studies, Survivor Cohorts, and Significant Contributions to the Field of Late Effects. Pediatric Clinics of North America. 2020 Dec 1;67(6):1033–49.

7. Jensen KS, Klug Albertsen B, Schrøder H, Zalounina Falborg A, Schmiegelow K, Rosthøj S, et al. Health care utilisation following childhood acute lymphoblastic leukaemia: a population-based matched cohort study. BMJ Open. 2021 Nov 22;11(11):e049847.

8. Dixon SB, Chen Y, Yasui Y, Pui C-H, Hunger SP, Silverman LB, et al. Reduced Morbidity and Mortality in Survivors of Childhood Acute Lymphoblastic Leukemia: A Report From the Childhood Cancer Survivor Study. JCO. 2020 Jul 24;JCO.20.00493.

9. Sørensen GV, Winther JF, de Fine Licht S, Andersen KK, Holmqvist AS, Madanat-Harjuoja L, et al. Long-term risk of hospitalization among five-year survivors of childhood leukemia in the Nordic countries. J Natl Cancer Inst [Internet]. 2019 Feb 11; Available from: https://www.ncbi.nlm.nih.gov/pmc/articles/PMC6748786/pdf/djz016.pdf

10. Reulen RC, Guha J, Bright CJ, Henson KE, Feltbower RG, Hall M, et al. Risk of cerebrovascular disease among 13 457 five-year survivors of childhood cancer: A population-based cohort study. International Journal of Cancer. 2021;148(3):572–83.

11. Streefkerk N, Tissing WJE, Korevaar JC, van Dulmen-den Broeder E, Bresters D, van der Heiden-van der Loo M, et al. A detailed insight in the high risks of hospitalizations in long-term childhood cancer survivors-A Dutch LATER linkage study. PLoS ONE. 2020;15(5):e0232708.

12. Mulrooney DA, Hyun G, Ness KK, Bhakta N, Pui C-H, Ehrhardt MJ, et al. The changing burden of long-term health outcomes in survivors of childhood acute lymphoblastic leukaemia: a retrospective analysis of the St Jude Lifetime Cohort Study. Lancet Haematol. 2019 Jun;6(6):e306–16.

13. Mueller BA, Doody DR, Weiss NS, Chow EJ. Hospitalization and mortality among pediatric cancer survivors: a population-based study. Cancer Causes Control. 2018 Nov;29(11):1047–57.

For peer review only - http://bmjopen.bmj.com/site/about/guidelines.xhtml

14. Ou JY, Smits-Seemann RR, Kaul S, Fluchel MN, Sweeney C, Kirchhoff AC. Risk of hospitalization among survivors of childhood and adolescent acute lymphoblastic leukemia compared to siblings and a general population sample. Cancer Epidemiol. 2017;49:216–24.

15. Brewster DH, Clark D, Hopkins L, Bauer J, Wild SH, Edgar AB, et al. Subsequent hospitalisation experience of 5-year survivors of childhood, adolescent, and young adult cancer in Scotland: a population based, retrospective cohort study. Br J Cancer. 2014 Mar 4;110(5):1342–50.

16. Oudin C, Berbis J, Bertrand Y, Vercasson C, Thomas F, Chastagner P, et al. Prevalence and characteristics of metabolic syndrome in adults from the French childhood leukemia survivors' cohort: a comparison with controls from the French population. Haematologica. 2018 Apr;103(4):645–54.

17. Hau EM, Caccia JN, Kasteler R, Spycher B, Suter T, Ammann RA, et al. Cardiovascular disease after childhood acute lymphoblastic leukaemia: a cohort study. Swiss Medical Weekly [Internet]. 2019 Mar 10 [cited 2020 Oct 17];149(0910). Available from: https://smw.ch/article/doi/smw.2019.20012

18. Holmqvist AS, Moëll C, Hjorth L, Lindgren A, Garwicz S, Wiebe T, et al. Increased health care utilization by survivors of childhood lymphoblastic leukemia is confined to those treated with cranial or total body irradiation: a case cohort study. BMC Cancer. 2014 Jun 10;14:419.

19. McBride ML, Lorenzi MF, Page J, Broemeling A-M, Spinelli JJ, Goddard K, et al. Patterns of physician follow-up among young cancer survivors: report of the Childhood, Adolescent, and Young Adult Cancer Survivors (CAYACS) research program. Can Fam Physician. 2011 Dec;57(12):e482-490.

20. Chang WH, Katsoulis M, Tan YY, Mueller SH, Green K, Lai AG. Late effects of cancer in children, teenagers and young adults: Population-based study on the burden of 183 conditions, in-patient and critical care admissions and years of life lost. The Lancet Regional Health - Europe. 2021 Nov 14;100248.

21. Gibson TM, Mostoufi-Moab S, Stratton KL, Leisenring WM, Barnea D, Chow EJ, et al. Temporal patterns in the risk of chronic health conditions in survivors of childhood cancer diagnosed 1970-99: a report from the Childhood Cancer Survivor Study cohort. Lancet Oncol. 2018;19(12):1590–601.

22. Hann I, Vora A, Richards S, Hill F, Gibson B, Lilleyman J, et al. Benefit of intensified treatment for all children with acute lymphoblastic leukaemia: results from MRC UKALL XI and MRC ALL97 randomised trials. UK Medical Research Council's Working Party on Childhood Leukaemia. Leukemia. 2000 Mar;14(3):356–63.

23. Mitchell C, Payne J, Wade R, Vora A, Kinsey S, Richards S, et al. The impact of risk stratification by early bone-marrow response in childhood lymphoblastic leukaemia: results from the United Kingdom Medical Research Council trial ALL97 and ALL97/99. British Journal of Haematology. 2009;146(4):424–36.

24. Berrington de González A, Morton LM. Converting epidemiologic studies of cancer etiology to survivorship studies: approaches and challenges. Cancer Epidemiol Biomarkers Prev. 2012 Jun;21(6):875–80.

25. Johnston WT, Lightfoot TJ, Simpson J, Roman E. Childhood cancer survival: a report from the United Kingdom Childhood Cancer Study. Cancer Epidemiol. 2010 Dec;34(6):659–66.

For peer review only - http://bmjopen.bmj.com/site/about/guidelines.xhtml

26. UK Childhood Cancer Study Investigators. The United Kingdom Childhood Cancer Study: objectives, materials and methods. Br J Cancer. 2000 Feb 11;82(5):1073–102.

27. Smith A, Roman E, Simpson J, Ansell P, Fear NT, Eden T. Childhood leukaemia and socioeconomic status: fact or artefact? A report from the United Kingdom childhood cancer study (UKCCS). International Journal of Epidemiology. 2006 Dec 1;35(6):1504–13.

28. Kane E, Painter D, Smith A, Crouch S, Oliver S, Patmore R, et al. The impact of rheumatological disorders on lymphomas and myeloma: a report on risk and survival from the UK's population-based Haematological Malignancy Research Network. Cancer Epidemiology. 2019 Apr 1;59:236–43.

29. Lamb MJ, Smith A, Painter D, Kane E, Bagguley T, Newton R, et al. Health impact of monoclonal gammopathy of undetermined significance (MGUS) and monoclonal B-cell lymphocytosis (MBL): findings from a UK population-based cohort. BMJ Open. 2021 Feb 22;11(2):e041296.

30. Chessells JM, Harrison CJ, Watson SL, Vora AJ, Richards SM. Treatment of infants with lymphoblastic leukaemia: results of the UK Infant Protocols 1987–1999. British Journal of Haematology. 2002;117(2):306–14.

31. Inaba H, Pui C-H. Advances in the Diagnosis and Treatment of Pediatric Acute Lymphoblastic Leukemia. Journal of Clinical Medicine. 2021 Jan;10(9):1926.

32. Brown P, Inaba H, Annesley C, Beck J, Colace S, Dallas M, et al. Pediatric Acute Lymphoblastic Leukemia, Version 2.2020, NCCN Clinical Practice Guidelines in Oncology. J Natl Compr Canc Netw. 2020 Jan;18(1):81–112.

33. Smith L, Glaser AW, Peckham D, Greenwood DC, Feltbower RG. Respiratory morbidity in young people surviving cancer: Population-based study of hospital admissions, treatment-related risk factors and subsequent mortality. Int J Cancer. 2019 01;145(1):20–8.

34. Reulen RC, Frobisher C, Winter DL, Kelly J, Lancashire ER, Stiller CA, et al. Long-term risks of subsequent primary neoplasms among survivors of childhood cancer. JAMA. 2011 Jun 8;305(22):2311–9.

35. Fidler MM, Reulen RC, Winter DL, Kelly J, Jenkinson HC, Skinner R, et al. Long term cause specific mortality among 34 489 five year survivors of childhood cancer in Great Britain: population based cohort study. BMJ. 2016 Sep 1;354:i4351.

36. Andrés-Jensen L, Skipper MT, Mielke Christensen K, Hedegaard Johnsen P, Aagaard Myhr K, Kaj Fridh M, et al. National, clinical cohort study of late effects among survivors of acute lymphoblastic leukaemia: the ALL-STAR study protocol. BMJ Open [Internet]. 2021 Feb 9 [cited 2021 May 4];11(2). Available from: https://www.ncbi.nlm.nih.gov/pmc/articles/PMC7875271/

37. van Erp LME, Maurice-Stam H, Kremer LCM, Tissing WJE, van der Pal HJH, de Vries ACH, et al. A vulnerable age group: the impact of cancer on the psychosocial well-being of young adult childhood cancer survivors. Support Care Cancer. 2021 Aug;29(8):4751–61.

For peer review only

For peer review only - http://bmjopen.bmj.com/site/about/guidelines.xhtml

Legends

Table 1: Studies reporting morbidity among 5-year survivors of leukaemia diagnosed in children, teenagers, and young adults; both acute lymphoblastic leukaemia (ALL) alone, and all leukaemias combined

Table 2: Characteristics of acute lymphoblastic leukaemia (ALL) cases diagnosed <15 years in England 1992-96 and their age- and sex-matched first-choice controls[1]: total registered in the original case-control study, and 5-year survivors included in the present study

Figure 1: Flowchart showing subjects targeted in the original United Kingdom Childhood Cancer case-control study and those included in the present cohort

Figure 2: Outpatient activity hazard rates per year and 95% confidence intervals (dotted lines) for childhood (< 15 years) ALL 5-year survivors and their matched controls: A) paediatrics, haematology and oncology, B) all other clinical specialties

Figure 3: Cumulative incidence (%), incidence rates (per 1000 person-years), attributable risks, and incidence rate ratios for the top 15 outpatient specialties (excluding paediatrics, haematology and oncology) with two or more face-to-face speciality-specific visits in the 5 to 25 years following diagnosis (cases diagnosed <15 years, 1992-96) and their matched population controls

Figure 4: Cumulative incidence (%), incidence rates (per 1000 person-years), attributable risks, and incidence rate ratios for the top 15 inpatient specialties (excluding paediatrics, haematology and oncology) with one or more specialty-specific admissions in the 5 to 25 years following diagnosis (cases diagnosed aged<15 years, 1992-96) and their matched population controls.

Supplementary Figure 1: Hazard rates per year of activity at paediatrics, haematology and oncology among ALL cases who survived 5 years or more and their matched population controls: UKCCS, ALL diagnosed aged<15 years, 1992-96.

Supplementary Figure 2: Cumulative incidence (%), incidence rates (per 1000 person-years), attributable risks and incidence rate ratios for the top 15 outpatient specialties (excluding paediatrics, haematology and oncology) with two of more face-to-face speciality-specific visits in the 5 to 15 years and 15 to 25 years following diagnosis (cases diagnosed <15 years, 1992-96) and their matched population controls.

For peer review only - http://bmjopen.bmj.com/site/about/guidelines.xhtml

Table 1: Studies reporting morbidity among 5-year survivors of leukaemia diagnosed in children, teenagers, and young adults; both acute lymphoblastic leukaemia (ALL) alone, and all leukaemias combined

Study	5-year survivors: Setting, Timeframe	5-year survivors: Numbers, Completeness	Comparator Source	Follow-up[1] Source, Timing post-diagnosis	Outcome Measures	Main Findings
**Acute lymphoblastic leukaemia**						
Childhood Cancer Survivor Study, CCSS (8)	USA & Canada: 31 centres<br><br>1970-1999	6148 <21yrs<br><br>~72% consented at baseline	5051 controls<br><br>Closest aged siblings in random sample of all childhood cancer survivors	Biannual questionnaires 2000-2017<br><br>5-46yrs post-diagnosis (median 22yrs)	Conditions considered severe by the respondents, distributed by treatment era and treatment group	11% of survivors diagnosed in the 1990s receiving standard treatment reported at least one severe chronic condition in the 20yr after diagnosis; Rate Ratio 1.9 (95%CI 1.5-2.3) compared to controls. RRs>2 for stroke, major joint replacement, and diabetes
Adult Life after Childhood Cancer in Scandinavia, ALiCCS[2] (9)	Denmark, Sweden, Iceland & Finland: National cancer registries<br><br>1970-2008[2]	3391 <20yrs<br><br>100% traced	129 828 controls matched on sex, age, and country<br><br>National population registers	National hospital registers 1975-2012<br><br>5-42yrs post-diagnosis (median 16yrs)	First inpatient admission for 120 conditions, total and by organ system	Survivors were nearly twice (Rate Ratio=1.95, 95%CI 1.83-2.07) as likely to be hospitalised for ≥1 of 120 conditions than controls. RRs>2 for diseases of the endocrine, nervous, and circulatory systems; infections, blood/blood organs, second cancers, and benign neoplasms
St Jude Lifetime Cohort, SJLIFE (12)	USA: St Jude Children's Research Hospital<br><br>1963-2003	980 <18yrs alive 10 years after diagnosis<br><br>~67% consented	272 controls<br><br>Friends, non-first-degree relatives of hospital patients, hospital employees and volunteers	In-person interview 2015<br><br>12-52yrs post-diagnosis (median 25yrs)	Conditions considered by respondents to be moderate/severe, overall, and by organ system	By age 30, survivors reported 3.2 (95%CI 2.9-3.4) chronic conditions compared with 1.2 (95%CI 1.0-1.4) among controls. Conditions varied by treatment era, with endocrine and musculoskeletal conditions contributing more to the cumulative burden in the 1990s and 2000s than in earlier decades
Nordic Society of Paediatric Haematology and Oncology ALL registry, NOPHO (7)	Denmark: Acute lymphoblastic leukaemia national registry<br><br>1994-2015	675 aged 1-<18yrs alive 2.5 years after diagnosis<br><br>~96.4% traced	6750 controls matched on age, sex and region<br><br>National population register	National primary care register 1997-2018 and national hospital registers 1997-2017<br><br>2.5-24yrs post diagnosis (median 13.5yrs)	Rates of primary care and hospital (in/outpatient) contacts, by year since diagnosis (2.5-17.5yrs post-diagnosis)	At 2-3yrs post diagnosis, survivors had 14.2 hospital contacts (95%CI 13.4-15.1), 30 times more than controls (Incidence Rate Ratio=31.5, 95%CI 28.3-35.1); contacts decreased over time but remained above baseline through to end of follow-up (IRRs≥2). For primary care, survivors started with 4.75 (95%CI 4.41-5.11) daytime contacts, nearly twice that among controls (IRR=1.85, 95%CI 1.71-2.00); contacts remained at 3-5 times/yr but IRRs declined

For peer review only - http://bmjopen.bmj.com/site/about/guidelines.xhtml

Study	5-year survivors		Comparator Source	Follow-up[1] Source, Timing post-diagnosis	Outcome Measures	Main Findings
	Setting, Timeframe	Numbers, Completeness				
						and were not statistically significant >12yrs post-diagnosis.
Swiss Childhood Cancer Survivor Study, SCCSS (17)	Switzerland: Swiss Childhood Cancer Registry<br><br>1976-2005	511 <16yrs, alive in 2007 and aged≥16yrs at questionnaire<br><br>72% participated	702 siblings	Questionnaire 2007-2012<br><br>5- 36yrs post-diagnosis	Self-reported cardiovascular diseases	14% of survivors reported having at least one cardiovascular disease, nearly twice that of their siblings (Odds Ratio=1.9, 95%CI 1.3-2.8)
Childhood, adolescent and young adult cancer survivors, CAYACS (19)	Canada: British Columbia cancer registry[4]<br><br>1970-1992	242 <20yrs alive at 31 December 2000<br><br>~64% traced	11 570 controls frequency-matched on age and sex<br><br>Health insurance plan	Health insurance claims 1998-2000<br><br>8-20yrs post-diagnosis	Physician visits over three-year period excluding oncology, overall and by specialty	Survivors were nearly twice as likely as controls to visit a doctor at least once (Rate Ratio=1.95, 95%CI 1.8-2.1). RRs≥2 were found for ≥1 visit to neurology, ophthalmology, otolaryngology, internal medicine, surgery, urology, psychiatry, and dermatology
Childhood Cancer Register of Southern Sweden, BORISS (18)	Sweden: Regional cancer registry<br><br>1970-1999	213 <18yrs<br><br>~100% traced	10 650 controls matched on sex, year of birth and county<br><br>National population register	National hospital registers inpatient: 1975-2009, outpatient: 2001-2009<br><br>5-33yrs post-diagnosis (median 16yrs)	Hospital contacts (in/outpatient), overall and for 20 disease categories	Survivors were over twice as likely to have ≥1 hospital contact than controls (Odds Ratio=2.5, 95%CI 1.5-4.3), and were more than twice as likely to consult for disorders of the blood, endocrine system, nervous system, skin, infections or benign neoplasms
University of Utah-Intermountain Healthcare (14)	USA: Primary Children's Hospital, Salt Lake City<br><br>1998-2008	176 <22yrs<br><br>~79% traced	503 controls matched on sex and year of birth<br><br>State population register	State hospital registers 2003-2013<br><br>5-10yrs post-diagnosis (median 9yrs)	Rate of inpatient admissions over a maximum of five years	In the 5 to 10 years after diagnosis, the hospitalisation rate of survivors was 3.76 per 100 person-years (95%CI 2.22-6.36), twice the rate among controls (Relative Hospitalisation Rate=2.01, 95%CI 1.00-4.03)
**<u>All leukaemias combined</u>**						
British Childhood Cancer Survivor Study, BCCSS (10)	UK: National Registry of Childhood Tumours<br><br>1940-1991	3544 <15yrs, alive in April 1997<br><br>~87% being followed	Hospitalisation rates for cerebrovascular disease<br><br>National population and hospital registers	National hospital registers 1997-2012<br><br>5-72yrs post-diagnosis	Cerebrovascular disease recorded in hospital discharge summaries	Survivors were almost five times more likely to have been hospitalised for cerebrovascular disease than expected (Age Standardised Hospitalisation Ratio=4.7, 95%CI 3.6-6.1)

For peer review only - http://bmjopen.bmj.com/site/about/guidelines.xhtml

Study	5-year survivors: Setting, Timeframe	5-year survivors: Numbers, Completeness	Comparator Source	Follow-up[1] Source, Timing post-diagnosis	Outcome Measures	Main Findings
Dutch Childhood Oncology Group-LATER Study (11)	Netherlands: Paediatric oncology or haematology centres<br><br>1963-2001	1900 <18yrs, alive in January 1995<br><br>~92% traced	38 000 controls matched on sex and year of birth<br><br>National population register	National hospital registers 1995-2015<br><br>5-52yrs post-diagnosis	Rate of inpatient admissions over a maximum of ten years	The hospitalisation rate of survivors was almost twice that of controls (Relative Hospitalisation Rate=1.87, 95%CI 1.65-2.12)
Leucémies de l'Enfant et l'Adolescent Cohort, LEA (16)	France: 16 cancer centres<br><br>1980-2012	1025 <18yrs, alive in 2007 and aged≥18yrs at last LEA medical examination<br><br>72% participated	3203 controls matched on sex and age<br><br>Volunteers for free medical examinations	Medical examinations at 2yr intervals until 10 years post-diagnosis (or age 20), thereafter every 4yrs<br><br>2-34yrs post-diagnosis (mean 16yrs)	Metabolic syndrome found at medical examination	Prevalence of metabolic syndrome among survivors was 10.3%, more than twice that among controls (Odds Ratio=2.49, 95%CI 1.91-2.35)
Scottish Cancer Registry (15)	UK: Scottish cancer registry<br><br>1981-2003	884 <25yrs<br><br>~100% traced	Hospitalisation rates<br><br>National population and hospital registers	National hospital registers 1986-2009<br><br>5-28yrs post-diagnosis (mean 16yrs)	First inpatient admission for particular diseases, overall, and for selected disease categories	Survivors were almost four times more likely to have been hospitalised than expected (Standardised Hospitalisation Ratio=3.9, 95%CI 3.6-4.3). SHR≥2 for diseases of the endocrine, nervous, circulatory, respiratory and digestive systems; infections and neoplasms
Washington State Cancer Registry (13)	USA: Washington State cancer registry<br><br>1982-2008	815 <20yrs<br><br>Not evaluable	8150 controls matched on sex and year of birth and alive at case's diagnosis<br><br>State birth records	State hospital registers 1987-2013<br><br>5-27yrs post-diagnosis (median 9yrs)	Rates of inpatient admissions, and hospitalisation or death for selected disease categories	The survivors' hospitalisation rate was almost three times that of controls (Hazard Ratio=2.8, 95%CI 2.2-3.5). HRs≥2 were observed for hospitalisation or death from endocrine, nervous, circulatory, respiratory, digestive, genitourinary, skin and musculoskeletal systems; blood diseases infections and cancers
Clinical Practice Research Datalink (20)	UK: Clinical Practice Research Datalink<br><br>1998-2020	403 ≤25 years<br><br>Not evaluable	13517 controls matched to all cancers on sex, year of birth, socioeconomic status and general practice<br><br>Primary care registers	Primary care electronic health records linked to national hospital registers from age 18yrs to 2020<br><br>5-22yrs post-diagnosis	Mean cumulative counts of primary care visits and hospital admissions for 183 conditions between ages of 18 and 35 or 45, overall and by organ system	Survivors' mean count of primary care visits was higher than among controls between ages of 18-35yrs (23.5, 95%CI 19.8-29.3 compared to 4.0, 95%CI 3.9-4.1), and 18-45yrs (29.8, 95%CI 24.7-36.0 compared to 7.2, 95%CI 7.1-7.4); as were their mean counts of admissions (18-35yrs: 6.0, 95%CI 5.6-6.3 vs 1.1, 95%CI 1.0-1.1; 18-45yrs: 7.9, 95%CI 7.0-8.0 vs 1.8, 95%CI 1.8-1.9). Survivors' cumulative burdens

For peer review only - http://bmjopen.bmj.com/site/about/guidelines.xhtml

Study	5-year survivors		Comparator Source	Follow-up[1] Source, Timing post-diagnosis	Outcome Measures	Main Findings
	Setting, Timeframe	Numbers, Completeness				
						of cardiovascular conditions and endocrinopathies were higher than controls'.

[1]Minimum to maximum possible follow-up relative to diagnosis date.[2]Finland 1969-2012; Denmark 1977-2010; Sweden 1987-2009 with some regions starting in 1964-1987; Iceland 1999-2008. [3]Completeness could not be evaluated in the Washington State Cancer Registry Study as study subjects were not linked to a population register. [4]CAYACS reported outcomes for all childhood cancer survivors, of which 20% had AL

For peer review only

For peer review only - http://bmjopen.bmj.com/site/about/guidelines.xhtml

Table 2: Characteristics of acute lymphoblastic leukaemia (ALL) cases diagnosed <15 years in England 1992-96 and their age- and sex-matched first-choice controls[1]: total registered in the original case-control study, and 5-year survivors included in the present study

	Original study		5-year survivors	
	**Cases** N (%)	**Controls[1]** N (%)	**Cases** N (%)	**Controls[1]** N (%)
**Total**	1372 (100)	2524 (100)	1082 (100)	2018 (100)
**Sex**				
Male	763 (55.6)	1410 (55.9)	589 (54.4)	1099 (54.5)
Female	609 (44.4)	1114 (44.1)	493 (45.6)	919 (45.5)
**Lineage[1]**	-	-		
B-ALL	1234 (89.9)	-	1004 (92.8)	-
T-ALL	120 (8.7)	-	70 (6.5)	-
**Age (years) at:**				
Diagnosis				
<1	52 (3.8)	86 (3.4)	22 (2.0)	41(2.0)
1-4	839 (61.2)	1578 (62.5)	703 (65.0)	1331 (66.0)
5-9	267 (19.5)	484 (19.2)	220 (20.3)	402 (19.9)
10-14	214 (15.6)	376 (14.9)	137 (12.7)	244 (12.1)
Median (IQR)	4.5 (3.0-7.6)	4.4 (3.0-7.5)	4.4 (3.0-7.1)	4.3 (3.0-7.0)
Beginning of follow-up				
Median (IQR)	-	-	9.4 (8.0-12.1)	9.3 (8.0-12.0)
End of follow-up[2]				
Median (IQR)	-	-	29.6 (27.3-32.1)	29.5 (27.0-31.9)
**IMD[3] quintile at diagnosis**				
Least deprived (1-3)	834 (60.8)	1533 (60.7)	675 (62.4)	1237 (61.3)
Most deprived (4-5)	520 (37.9)	975 (38.6)	400 (37.0)	766 (38.0)
**Trial entry**				
Yes	1215 (88.6)	-	979 (90.5)	-
No	157 (11.4)	-	103 (9.5)	-
**Person-years in follow-up[2]**	-	-	19425.6	35966.0
**Deaths in follow-up**	-	-	115	10
Cumulative mortality, % (95%CI)			10.7 (9.0-12.8)	0.6 (0.3-1.1)
Mortality Rate Ratio (95%CI)			21.3 (11.2-45.6)	
**Cancer[4] registrations in follow-up**	-	-		
Total			60	23
Cumulative incidence, % (95%CI)			5.8 (4.5-7.4)	1.4 (0.9-2.0)
Incidence Rate Ratio (95%CI)			4.9 (3.0-8.9)	
Malignancies only			32	6
Cumulative incidence, % (95%CI)			3.1 (2.2-4.3)	0.4 (0.1-0.8)
Incidence Rate Ratio (95%CI)			9.9 (4.1-29.1)	

[1]Lineage was not confirmed for 18 cases, 8 of whom were alive 5 years after diagnosis.
[2]Follow-up time among controls was censored at the matched survivor's death if the survivor died during follow-up. IQR: interquartile range.
[3]IMD: Index of Multiple Deprivation income domain, IMD was missing for 18 cases and 16 controls.
[4]Total cancer, including tumours that were registered as benign, in situ and of uncertain behaviour: ICD-10 codes C00-C90, C92-C97 and D00-D48. Malignancies only: C00-C43, C45-C90, C92-C97 (NB registrations for lymphoid leukaemia (C91) were excluded). Cumulative incidence of cancer was calculated considering death as a competing risk.

For peer review only - http://bmjopen.bmj.com/site/about/guidelines.xhtml

For peer review only

Figure 1

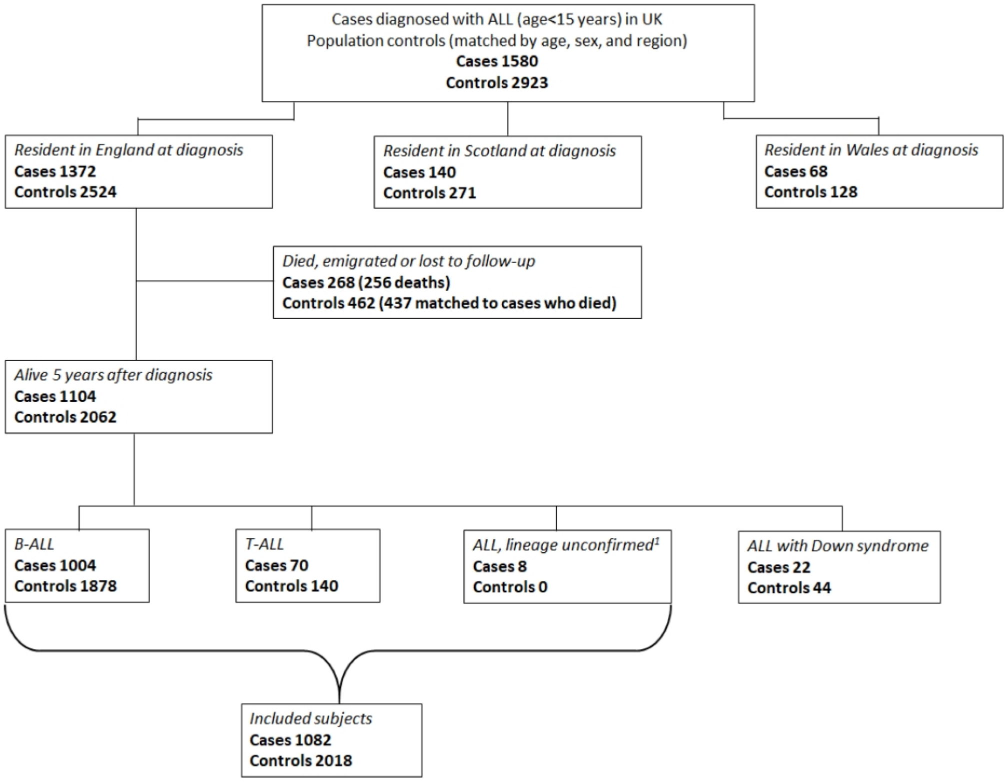

Figure 1: Flowchart showing subjects targeted in the original United Kingdom Childhood Cancer case-control study and those included in the present cohort

720x598mm (236 x 236 DPI)

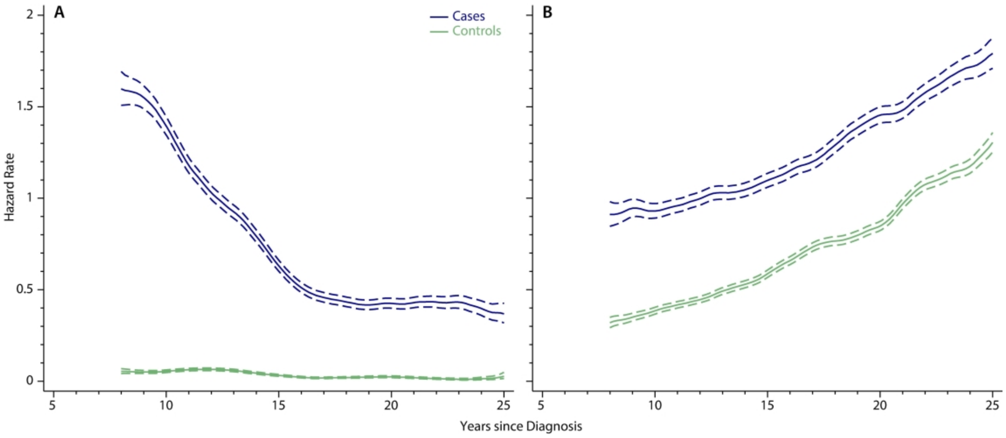

Figure 2: Outpatient activity hazard rates per year and 95% confidence intervals (dotted lines) for childhood (< 15 years) ALL 5-year survivors and their matched controls: A) paediatrics, haematology andoncology, B) all other clinical specialties

281x121mm (300 x 300 DPI)

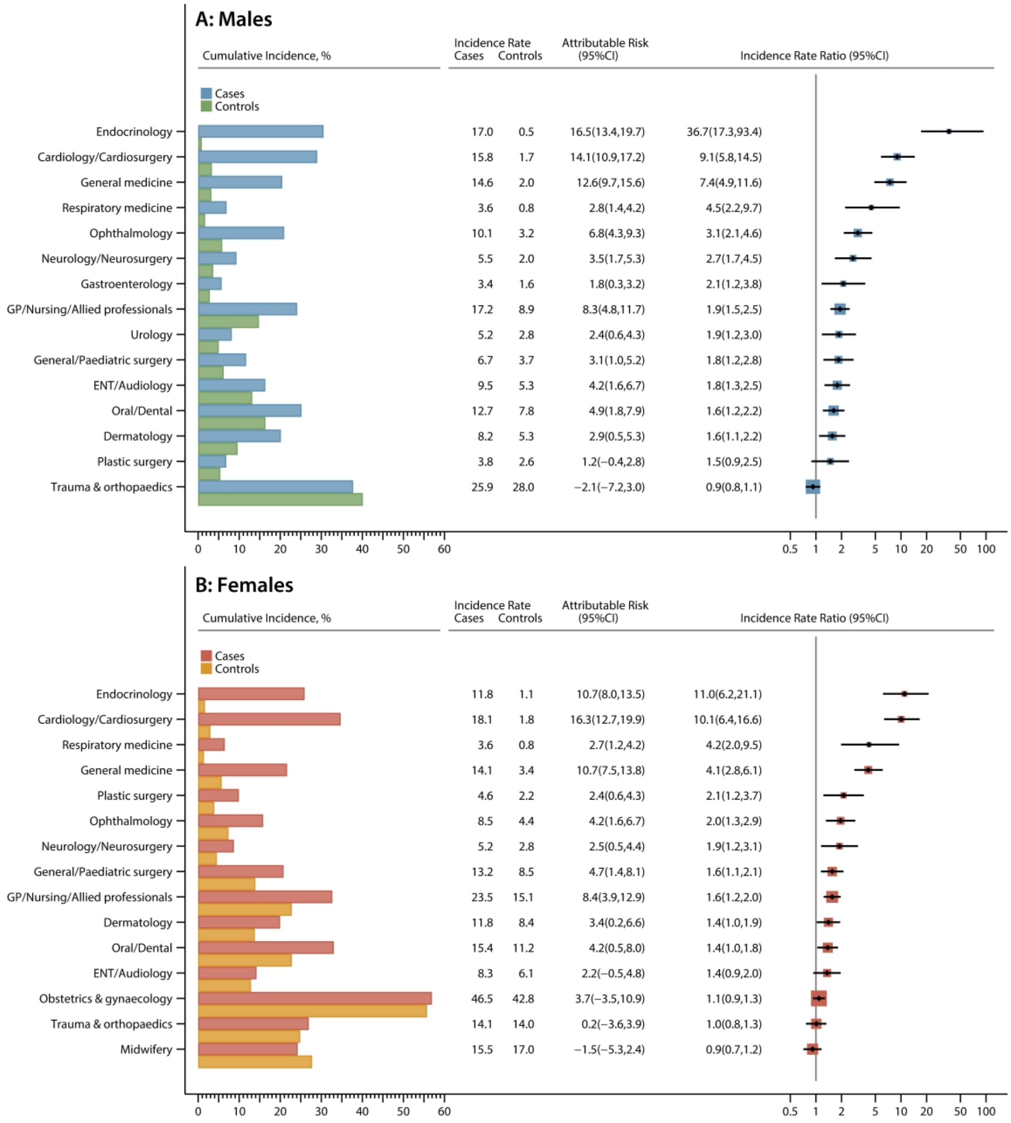

Figure 3: Cumulative incidence (%), incidence rates (per 1000 person-years), attributable risks, and incidence rate ratios for the top 15 outpatient specialties (excluding paediatrics, haematology and oncology) with two of more face-to-face speciality-specific visits in the 5 to 25 years following diagnosis (cases diagnosed <15 years, 1992-96) and their matched population controls

380x423mm (600 x 600 DPI)

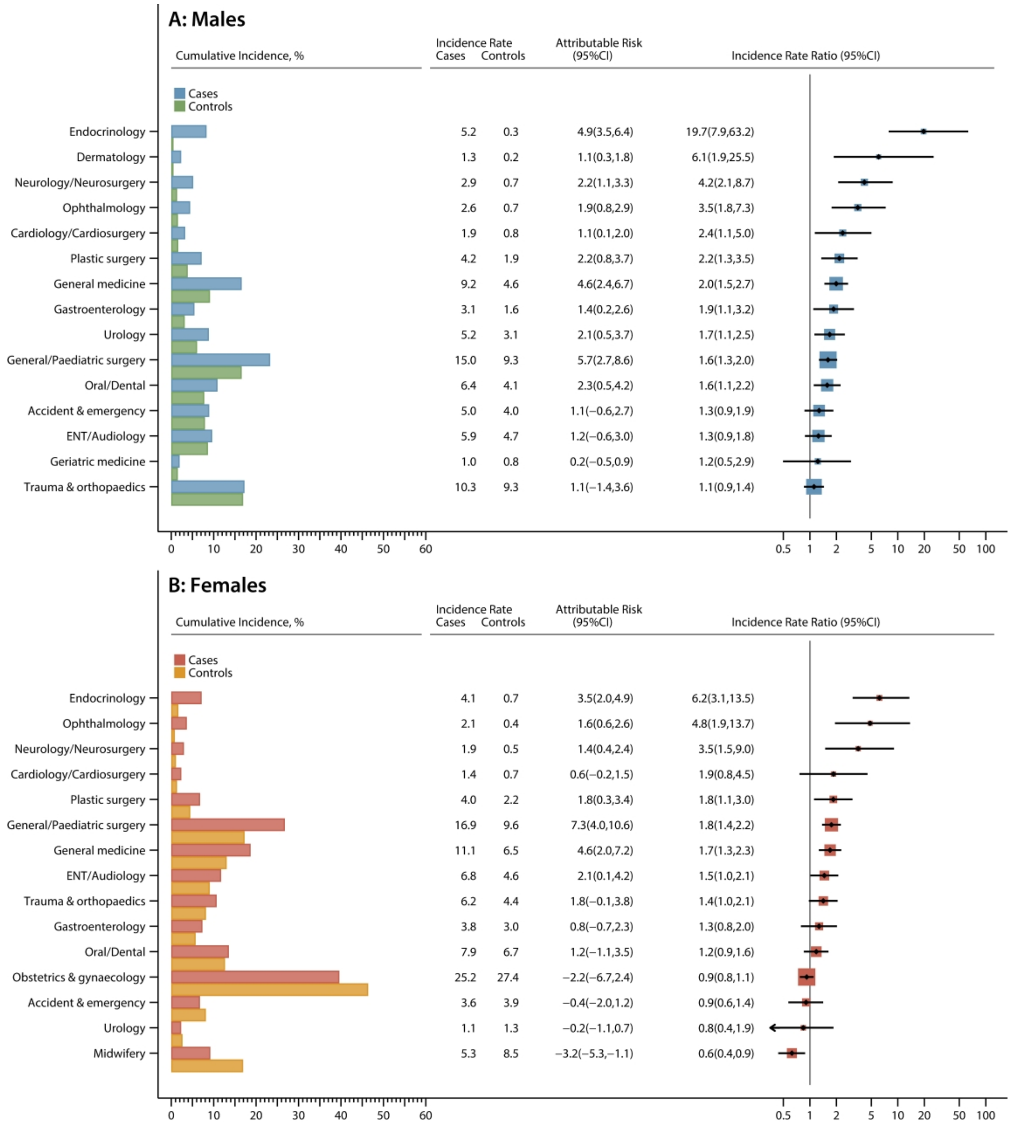

Figure 4: Cumulative incidence (%), incidence rates (per 1000 person-years) and incidence rate ratios for the top 15 inpatient specialties (excluding paediatrics, haematology and oncology) in the 5 to 25 years following diagnosis (cases diagnosed aged<15 years, 1992-96) and their matched population controls.

380x425mm (600 x 600 DPI)

Supplementary Figure 1: Hazard rates per year of activity at paediatrics, haematology and oncology among ALL cases who survived 5 years or more and their matched population controls: UKCCS, ALL diagnosed aged<15 years, 1992-96.

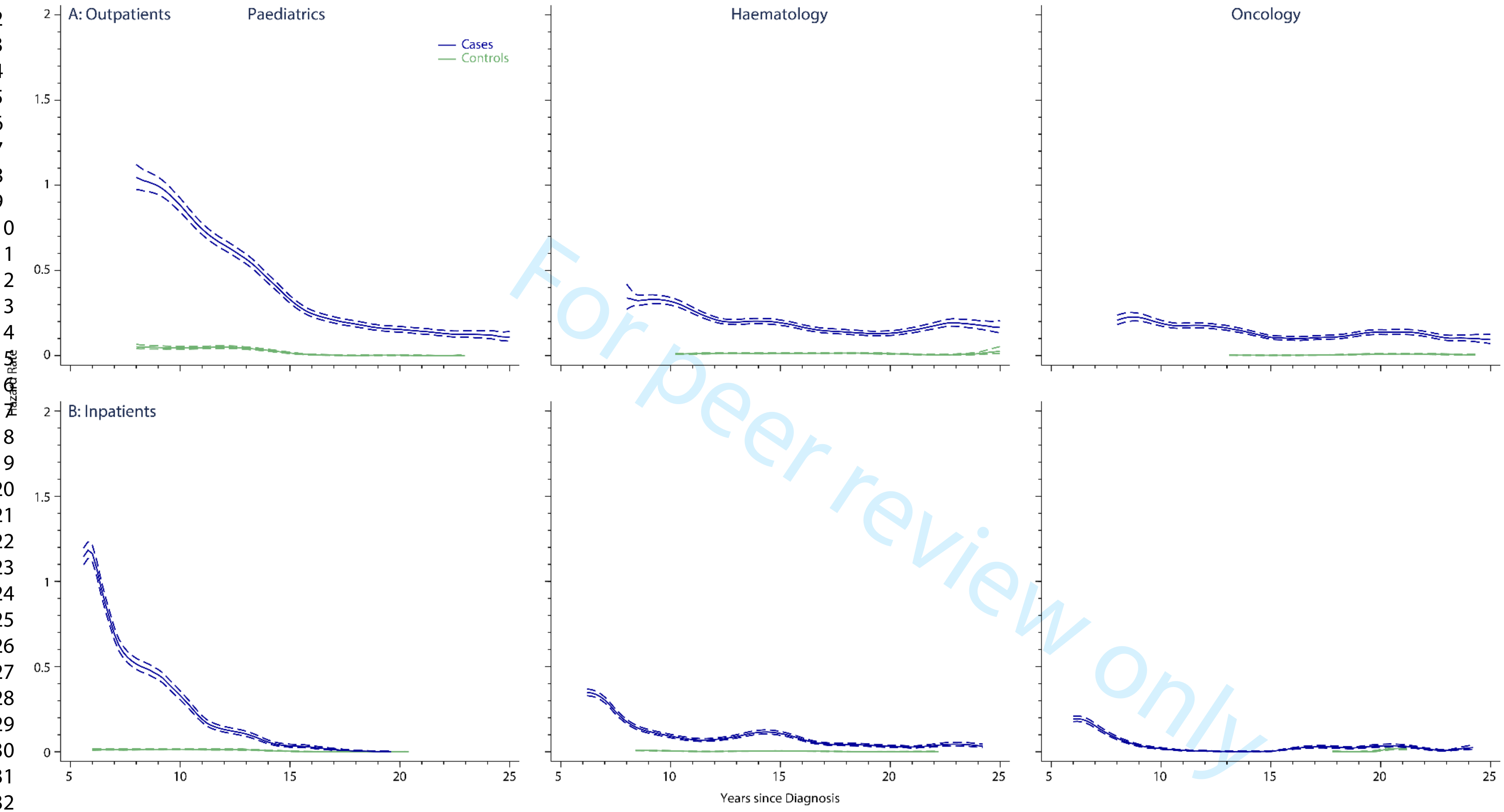

For peer review only

Supplementary Figure 2: Cumulative incidence, rates (per 1000 person-years), attributable risks & incidence rate ratios; top 15 outpatient specialties (excluding paediatrics, haematology & oncology) with ≥2 speciality-specific visits in the 5 to 15 years and 15 to 25 years following diagnosis (cases diagnosed <15 years, 1992-96) and their matched controls

A: Males

£15 Years

	Incidence Rate Cases	Incidence Rate Controls	Attributable Risk (95%CI)	Incidence Rate Ratio (95%CI)
Endocrinology	29.1	0.5	28.6(22.4,34.8)	61.3(20.3,303)
Cardiology/Cardiosurgery	19.0	1.1	17.9(13.0,22.9)	17.1(7.8,44.4)
General medicine	17.5	2.1	15.5(10.7,20.2)	8.5(4.6,16.9)
Ophthalmology	18.6	2.7	15.8(10.8,20.8)	6.8(3.9,12.4)
Respiratory medicine	3.6	0.6	3.0(0.8,5.1)	5.7(1.7,24.1)
Neurology/Neurosurgery	6.4	1.8	4.6(1.7,7.5)	3.6(1.7,8.3)
GP/Nursing/Allied professionals	13.9	4.0	9.8(5.5,14.2)	3.5(2.1,5.9)
Gastroenterology	2.1	0.6	1.4(−0.2,3.1)	3.3(0.8,15.2)
Oral/Dental	21.1	8.6	12.5(6.9,18.2)	2.5(1.7,3.6)
General/Paediatric surgery	8.7	3.6	5.1(1.6,8.7)	2.4(1.3,4.5)
Urology	4.5	2.3	2.3(−0.3,4.9)	2.0(0.9,4.5)
Plastic surgery	4.8	2.4	2.4(−0.3,5.0)	2.0(0.9,4.3)
ENT/Audiology	12.2	6.4	5.8(1.5,10.2)	1.9(1.2,3.1)
Dermatology	11.9	8.2	3.8(−0.6,8.2)	1.5(0.9,2.3)
Trauma & orthopaedics	38.0	38.7	−0.7(−9.6,8.1)	1.0(0.8,1.2)

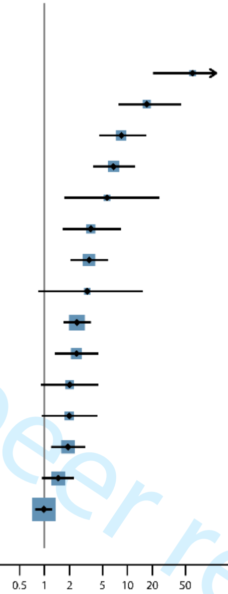

>15–25 Years

	Incidence Rate Cases	Incidence Rate Controls	Attributable Risk (95%CI)	Incidence Rate Ratio (95%CI)
Endocrinology	7.5	0.5	7.0(4.2,9.8)	16.4(5.7,64.5)
General medicine	12.1	1.9	10.2(6.5,13.8)	6.4(3.5,12.1)
Cardiology/Cardiosurgery	12.9	2.2	10.7(6.8,14.7)	5.8(3.3,10.6)
Respiratory medicine	3.5	0.9	2.6(0.8,4.5)	3.9(1.6,10.5)
Neurology/Neurosurgery	4.9	2.2	2.7(0.4,5.0)	2.2(1.1,4.3)
Gastroenterology	4.3	2.3	2.0(−0.1,4.1)	1.9(1.0,3.7)
Urology	5.8	3.2	2.6(0.0,5.1)	1.8(1.0,3.2)
Dermatology	5.3	3.1	2.3(−0.3,4.8)	1.7(0.9,3.2)
ENT/Audiology	7.4	4.5	2.8(−0.2,5.9)	1.6(1.0,2.7)
GP/Nursing/Allied professionals	20.2	12.9	7.3(2.0,12.7)	1.6(1.1,2.1)
General/Paediatric surgery	5.2	3.7	1.5(−1.0,4.1)	1.4(0.8,2.5)
Plastic surgery	3.1	2.7	0.4(−1.6,2.3)	1.1(0.5,2.3)
Ophthalmology	3.7	3.6	0.1(−2.2,2.3)	1.0(0.5,2.0)
Trauma & orthopaedics	15.1	18.0	−2.9(−8.4,2.6)	0.8(0.6,1.2)
Oral/Dental	6.1	7.3	−1.2(−4.3,1.9)	0.8(0.5,1.4)

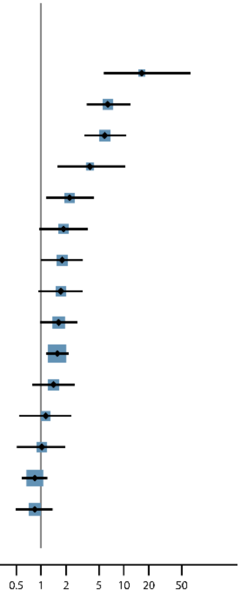

B: Females

£15 Years

	Incidence Rate Cases	Incidence Rate Controls	Attributable Risk (95%CI)	Incidence Rate Ratio (95%CI)
Endocrinology	20.0	0.7	19.2(13.7,24.7)	26.7(9.8,102)
Cardiology/Cardiosurgery	20.3	1.9	18.5(12.9,24.1)	10.8(5.4,23.8)
Respiratory medicine	5.0	0.6	4.5(1.8,7.2)	8.9(2.5,48.5)
General medicine	16.1	2.6	13.5(8.5,18.5)	6.1(3.3,12.1)
Plastic surgery	6.1	2.3	3.8(0.6,7.0)	2.7(1.2,6.1)
Dermatology	17.4	7.4	10.0(4.5,15.6)	2.4(1.5,3.7)
General/Paediatric surgery	12.8	5.7	7.1(2.4,11.8)	2.2(1.3,3.8)
GP/Nursing/Allied professionals	13.4	6.3	7.1(2.3,11.9)	2.1(1.3,3.5)
Neurology/Neurosurgery	5.0	2.6	2.3(−0.6,5.3)	1.9(0.8,4.3)
Ophthalmology	10.8	5.9	4.8(0.4,9.3)	1.8(1.1,3.1)
ENT/Audiology	10.2	6.0	4.2(−0.1,8.5)	1.7(1.0,2.9)
Oral/Dental	22.6	13.9	8.7(2.1,15.4)	1.6(1.1,2.3)
Trauma & orthopaedics	20.0	16.4	3.6(−2.9,10.1)	1.2(0.8,1.7)
Obstetrics & gynaecology	27.9	28.5	−0.6(−8.5,7.3)	1.0(0.7,1.3)
Midwifery	3.1	5.1	−1.9(−4.7,0.9)	0.6(0.3,1.4)

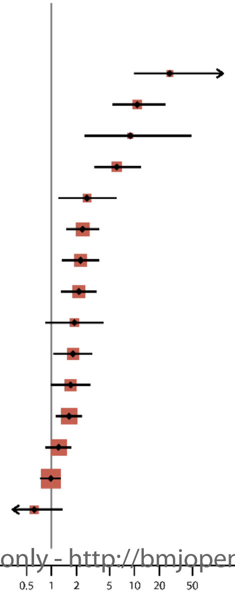

>15–25 Years

	Incidence Rate Cases	Incidence Rate Controls	Attributable Risk (95%CI)	Incidence Rate Ratio (95%CI)
Cardiology/Cardiosurgery	16.1	1.7	14.4(9.7,19.0)	9.3(4.9,18.7)
Endocrinology	5.9	1.3	4.6(1.9,7.2)	4.5(2.0,10.7)
General medicine	12.4	4.0	8.4(4.3,12.4)	3.1(1.9,5.2)
Respiratory medicine	2.5	1.0	1.5(−0.2,3.2)	2.4(0.9,7.0)
Ophthalmology	6.9	3.2	3.7(0.7,6.6)	2.1(1.2,3.9)
Neurology/Neurosurgery	5.4	2.8	2.5(−0.1,5.2)	1.9(1.0,3.7)
Plastic surgery	3.6	2.1	1.5(−0.7,3.6)	1.7(0.8,3.7)
GP/Nursing/Allied professionals	32.6	22.3	10.3(2.9,17.6)	1.5(1.1,1.9)
General/Paediatric surgery	13.5	10.7	2.8(−1.8,7.5)	1.3(0.9,1.9)
Obstetrics & gynaecology	66.6	58.0	8.6(−3.7,21.0)	1.1(0.9,1.4)
ENT/Audiology	6.9	6.2	0.6(−2.6,3.9)	1.1(0.6,1.8)
Oral/Dental	9.8	9.1	0.7(−3.4,4.9)	1.1(0.7,1.7)
Midwifery	25.3	26.6	−1.3(−7.8,5.2)	1.0(0.7,1.2)
Dermatology	7.4	9.1	−1.8(−5.4,1.9)	0.8(0.5,1.3)
Trauma & orthopaedics	9.6	12.1	−2.5(−6.8,1.8)	0.8(0.5,1.2)

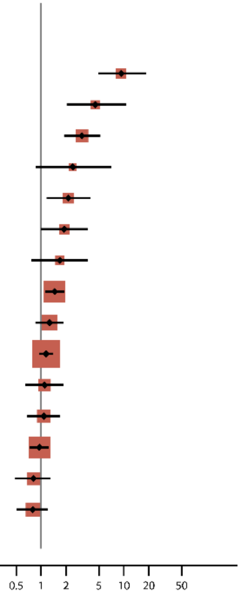

For peer review only

STROBE Statement—Checklist of items that should be included in reports of ***cohort studies***

	Item No	Recommendation	Page No
**Title and abstract**	1	(*a*) Indicate the study's design with a commonly used term in the title or the abstract	2&3
		(*b*) Provide in the abstract an informative and balanced summary of what was done and what was found	2
**Introduction**			
Background/rationale	2	Explain the scientific background and rationale for the investigation being reported	3
Objectives	3	State specific objectives, including any prespecified hypotheses	3
**Methods**			
Study design	4	Present key elements of study design early in the paper	3-4
Setting	5	Describe the setting, locations, and relevant dates, including periods of recruitment, exposure, follow-up, and data collection	3-4
Participants	6	(*a*) Give the eligibility criteria, and the sources and methods of selection of participants. Describe methods of follow-up	3-4
		(*b*) For matched studies, give matching criteria and number of exposed and unexposed	3-4; Fig 1
Variables	7	Clearly define all outcomes, exposures, predictors, potential confounders, and effect modifiers. Give diagnostic criteria, if applicable	3-5
Data sources/ measurement	8*	For each variable of interest, give sources of data and details of methods of assessment (measurement). Describe comparability of assessment methods if there is more than one group	4-5
Bias	9	Describe any efforts to address potential sources of bias	3-4
Study size	10	Explain how the study size was arrived at	3-4
Quantitative variables	11	Explain how quantitative variables were handled in the analyses. If applicable, describe which groupings were chosen and why	3-4
Statistical methods	12	(*a*) Describe all statistical methods, including those used to control for confounding	3-4
		(*b*) Describe any methods used to examine subgroups and interactions	NA
		(*c*) Explain how missing data were addressed	Fig 1
		(*d*) If applicable, explain how loss to follow-up was addressed	Fig 1
		(*e*) Describe any sensitivity analyses	NA
**Results**			
Participants	13*	(a) Report numbers of individuals at each stage of study—eg numbers potentially eligible, examined for eligibility, confirmed eligible, included in the study, completing follow-up, and analysed	Tab 1
		(b) Give reasons for non-participation at each stage	3
		(c) Consider use of a flow diagram	Fig 1
Descriptive data	14*	(a) Give characteristics of study participants (eg demographic, clinical, social) and information on exposures and potential confounders	Tab 1
		(b) Indicate number of participants with missing data for each variable of interest	3-4; Fig 1
		(c) Summarise follow-up time (eg, average and total amount)	Tab 1
Outcome data	15*	Report numbers of outcome events or summary measures over time	5-6; Tab 2 Fig 1

For peer review only - http://bmjopen.bmj.com/site/about/guidelines.xhtml

Main results	16	(*a*) Give unadjusted estimates and, if applicable, confounder-adjusted estimates and their precision (eg, 95% confidence interval). Make clear which confounders were adjusted for and why they were included	5-6
		(*b*) Report category boundaries when continuous variables were categorized	5-6
		(*c*) If relevant, consider translating estimates of relative risk into absolute risk for a meaningful time period	Figs 3-4
Other analyses	17	Report other analyses done—eg analyses of subgroups and interactions, and sensitivity analyses	6
**Discussion**			
Key results	18	Summarise key results with reference to study objectives	6-7
Limitations	19	Discuss limitations of the study, taking into account sources of potential bias or imprecision. Discuss both direction and magnitude of any potential bias	6-7
Interpretation	20	Give a cautious overall interpretation of results considering objectives, limitations, multiplicity of analyses, results from similar studies, and other relevant evidence	2; 6-7
Generalisability	21	Discuss the generalisability (external validity) of the study results	7
**Other information**			
Funding	22	Give the source of funding and the role of the funders for the present study and, if applicable, for the original study on which the present article is based	7

*Give information separately for exposed and unexposed groups.

Note: An Explanation and Elaboration article discusses each checklist item and gives methodological background and published examples of transparent reporting. The STROBE checklist is best used in conjunction with this article (freely available on the Web sites of PLoS Medicine at http://www.plosmedicine.org/, Annals of Internal Medicine at http://www.annals.org/, and Epidemiology at http://www.epidem.com/). Information on the STROBE Initiative is available at http://www.strobe-statement.org.

For peer review only
